# Bridging the gap between transition metal- and bio-catalysis via aqueous micellar catalysis

**DOI:** 10.1038/s41467-019-09751-4

**Published:** 2019-05-15

**Authors:** Margery Cortes-Clerget, Nnamdi Akporji, Jianguang Zhou, Feng Gao, Pengfei Guo, Michael Parmentier, Fabrice Gallou, Jean-Yves Berthon, Bruce H. Lipshutz

**Affiliations:** 1 Department of Chemistry and Biochemistry, University of California, Santa Barbara, CA 93106 USA; 2 Chemical and Analytical Development, Suzhou Novartis Pharma Technology Company Limited, 215537 Changshu, Jiangsu China; 3 Chemical & Analytical Development, Novartis Pharma AG, 4056 Basel, Switzerland; 4 GREENTECH, Biopôle Clermont Limagne, 63360 St Beauzire, France

**Keywords:** Homogeneous catalysis, Enzymes, Sustainability

## Abstract

Previous studies have shown that aqueous solutions of designer surfactants enable a wide variety of valuable transformations in synthetic organic chemistry. Since reactions take place within the inner hydrophobic cores of these tailor-made nanoreactors, and products made therein are in dynamic exchange between micelles through the water, opportunities exist to use enzymes to effect secondary processes. Herein we report that ketone-containing products, formed via initial transition metal-catalyzed reactions based on Pd, Cu, Rh, Fe and Au, can be followed in the same pot by enzymatic reductions mediated by alcohol dehydrogenases. Most noteworthy is the finding that nanomicelles present in the water appear to function not only as a medium for both chemo- and bio-catalysis, but as a reservoir for substrates, products, and catalysts, decreasing noncompetitive enzyme inhibition.

## Abstract (web summary)

Chemo-enzymatic tandem processes allow access to more complex non-racemic products. Here, the authors show that non-ionic surfactants, especially the tailor-made surfactant TPGS-750-M, enhance the activity of alcohol dehydrogenase to afford stereopure products in a 1-pot reaction.

## Introduction

Within the toolbox of organic synthesis, bio-catalysis has proven to be an extremely efficient approach to inducing high levels of stereo-, chemo, and regio-selectivities^1–4^. While the very recent Nobel prize for “directed evolution” highlights the scope of accessible organic biotransformations^5,6^, engineering the reaction medium itself can also lead to significant improvements in terms of enzyme−substrate compatibility, ultimately affording higher yielding processes. Moreover, reaching a certain level of productivity and titer is key for practical and industrial applications, which can be fine-tuned with the proper medium. Bäckvall and coworkers described a hybrid system combining the immobilization of a lipase for the acylation of chiral amines and Pd nanoparticles for racemization of the untouched enantiomers, leading to an efficient metalloenzyme for dynamic kinetic resolution^7^. Starting from a nonchiral substrate, multicatalyst processes that combine both a chemo- and bio-catalysis sequence oftentimes require a two-phase system, and while well established^8^, the enzymatic component relies predominantly on the presence of water. Any approach where water only is the reaction medium can be quite limiting, as most reaction partners and catalysts are not soluble in aqueous media. Thus, reactions under such conditions either rely on educts that have some element of water solubility, or are assumed to take place on water. Processes involving equilibria, such as an esterification, are also typically prohibited. Nonaqueous bio-catalysis employing organic solvents has emerged to address these issues, especially focused on lipases^9^. Nevertheless, this change in medium is often associated with reduced reaction rates and inactivation or denaturation of the biocatalyst, in addition to the environmental impact associated with use of dipolar aprotic solvents or other VOCs. Greener alternatives for asymmetric biosynthesis^10^ are flourishing, including reliance on supercritical CO_2_^11^_,_ ionic liquids^12–14^ and deep eutectic solvents^15,16^ as bio-reaction media. Solvent-free processes^17^ are also employed, with their associated advantages and limitations. While the use of surfactants to prevent protein adhesion to glassware or to mimic in vivo conditions in biochemistry is far from new, most of the systems described rely on emulsions^18^ or reverse microemulsions^19^ using an organic or greasy solvent (e.g., hexane, octane) composing the hydrophobic phase of the system. Those methods are adapted most notably when enzymes and substrates have different solubilities, allowing for a larger contact surface, but by definition, do not avoid the use of organic solvents.

**Fig. 1:**
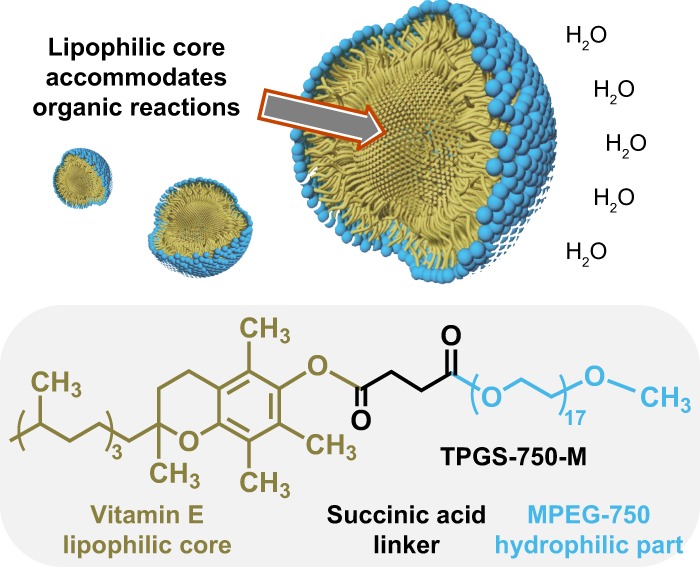
Nanoreactors for organic reactions. The designer surfactant TPGS-750-M forms micelles in water. The hydrophobic core, made of vitamin E, can accommodate organic reactions to take place in water, without the need of additional organic solvent

To begin to address the challenges of routinely performing organic synthesis in water under mild conditions, TPGS-750-M was introduced as a “benign by design” surfactant that assembles into atypically aggregated micelles within micelles^20^, averaging ca. 50 nm in size. This unusual array effectively enables numerous organic reactions with the overarching goal of “getting organic solvents out of organic chemistry” (Fig. 1)^21^. The hydrophobic core is composed of vitamin E that houses lipophilic substrates and transition metal catalysts.

Switching^22^ from an organic to an aqueous micellar medium has begun to shed light on new rules in effect for transition metal-catalyzed synthetic chemistry, and cross-coupling reactions in particular^23^, in water that are not applicable to conventional organic solvent-based reactions, such as the hydrophobic effect that accelerates reactions under mild conditions^24^. In initially testing the compatibility between nanoreactors composed of TPGS-750-M and alcohol dehydrogenase (ADH)^25–32^ in water, it was discovered that not only are these enzymes tolerant of the surfactant but its presence enhances enzymatic activity. One related application of TPGS-750-M to synthetic biotechnology by Balskus describes the acceleration of styrene production by *Escherichia coli* through interaction with the membrane of the whole cell, as well as a one-pot styrene fermentation then cyclopropanation, confirming that this surfactant is biocompatible^33,34^. We have begun to focus on performing chemo-enzymatic tandem processes to access more complex nonracemic products. While realization of multistep, one-pot processes is particularly attractive under green chemistry conditions, precedent for use of transition metal catalysis preceded or followed by enzymatic catalysis is still marginal, at best, mainly due to the incompatibility of reaction conditions (i.e., pH, temperature, solvent, relative concentrations, etc.)^35^. A few examples combining the use of alcohol dehydrogenase and Suzuki-Miyaura^36–40^ or Heck^41,42^ cross-coupling reactions have been reported in water or ionic liquids, but most require the exchange of reaction medium, high temperatures, or specifically designed catalysts. Here we describe an extensive study on the use of nonionic surfactants, and TPGS-750-M in particular, that enable one-pot cascade processes involving both chemo- and bio-conversions in the same aqueous medium.

## Results and discussion

### Aqueous micellar solutions as the reaction medium

**Fig. 2:**
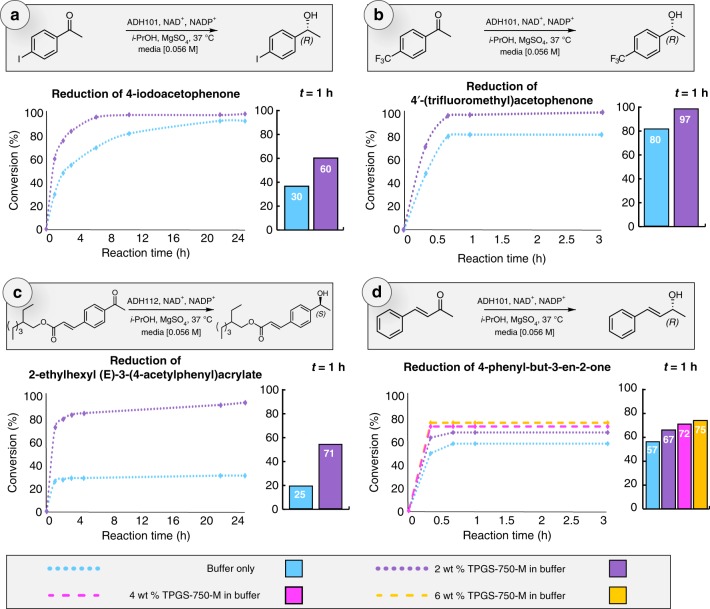
Ketone reductions with/without TPGS-750-M. For the four examples shown, conversions were monitored until plateauing (curves). A comparison at *t* = 1 h is also shown (bars). Reactions in buffer only are displayed in blue. Reactions in 2 wt % TPGS-750-M/buffer are displayed in purple. Reactions in 4 wt % TPGS-750-M/buffer are displayed in pink. Reactions in 6 wt % TPGS-750-M/buffer are displayed in yellow. Plot **a** describes the reduction of 4-iodoacetophenone, plot **b** shows the reduction of 4′-(trifluoromethyl)acetophenone, plot **c** illustrates the reduction of 2-ethylhexyl (*E*)-3-(4-acetylphenyl)acrylate, while plot **d** shows the reduction of 4-phenyl-3-en-2-one. Conversions were monitored by HPLC (case **a**) or ^1^H NMR (cases **b**, **c** and **d**). Source data are provided as Source Data File

To establish the compatibility of alcohol dehydrogenases and micelles derived from TPGS-750-M in water, asymmetric reductions of four different ketones have been evaluated. These educts included 4-iodoacetophenone (Fig. 2a), 4′-(trifluoromethyl)acetophenone (Fig. 2b), the product from a Heck coupling, 2-ethylhexyl (*E*)-3-(4-acetylphenyl)acrylate (Fig. 2c), and 4-phenyl-but-3-en-2-one (Fig. 2d); all reactions were performed in a phosphate buffer (0.2 M) at pH = 7 with and without 2wt % of TPGS-750-M. Remarkably, enzymatic superactivity^43^ is observed as the lipophilicity of the substrate increased. Indeed, the presence of the surfactant positively impacted the outcome of the reaction, leading to faster reaction rates. In the case of Fig. 2a, the reduction went to completion far more rapidly in the presence of micelles. Enantiomeric excesses were excellent (>99.8% ee) in both cases. By slightly increasing the lipophilicity of the substrate (Fig. 2b), the reaction plateaued at 80% conversion in buffer alone, while reaching completion in the corresponding aqueous surfactant solution. The same observation was made in Fig. 2c, with an even greater gap between both media (plateauing at 30 vs. 92%). The slope discontinuity after 1 h in buffer may indicate enzyme saturation. The phenomenon was reduced, or suppressed, at a given substrate concentration (20 mg of enzyme for 0.4 mmol of ketone [0.056 M]), in the presence of TPGS-750-M.

Our rationale for these observations is that under typical conditions, entrance to the enzymatic pocket is eventually hindered by the buildup of water-insoluble substrates and potentially as well due to the more lipophilic products, all looking to gain (re)entry. This leads to incomplete conversions in solely buffered aqueous media, as these data show. In the presence of micelle-forming TPGS-750-M, however, the micelles function both as a desirable solvent (vs. the surrounding water) and as a reservoir, housing and releasing substrates and products as a normal matter of dynamic exchange between micelles^44^. Educts undergoing such a phenomenon find their way to the far less encumbered enzymatic cavity, enabling eventual reduction. Thus, micelles in the buffer help to control both substrate and product concentrations in an aqueous medium, providing a measured supply that does not overwhelm the enzyme, thereby allowing for higher rates of conversion.

To further investigate this hypothesis, the impact of the concentration of TPGS-750-M on reaction rates associated with reduction of both an aryl ketone (Fig. 2c) and an enone (Fig. 2d) were assessed. The former, a keto ester, shows a dramatic difference between the extent of conversion in buffer vs. that in 2 wt % TPGS-750-M, raising the overall conversion from ca. 30 to over 90%. For the enone, also a challenging substrate for this particular enzyme, its reduction stops at 49% conversion under purely buffered conditions. While in-depth structural investigations would be needed to understand why such a dramatic stop to this reaction occurred, it is worth mentioning that, by increasing the amount of TPGS-750-M to 6 wt%, the conversion jumps to 75%. These results confirm that increasing the available volume of solvent in this aqueous medium helps to moderate enzyme saturation, supporting a reservoir effect.

**Fig. 3:**
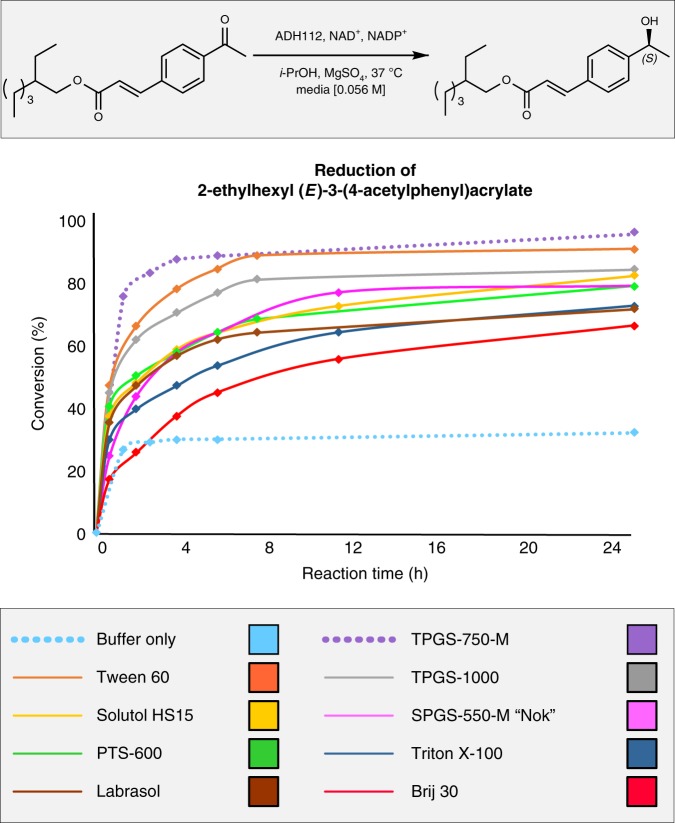
Comparisons of commercially available surfactants. The reduction of 2-ethylhexyl (*E*)-3-(4-acetylphenyl)acrylate by ADH112 was performed with a solution of 2 wt % various commercially available surfactants in a phosphate buffer [0.2 M] at pH = 7 and 37 °C. Conversions were monitored by ^1^H NMR. Source data are provided as Source Data File

Based on these early observations, several other commercially available nonionic surfactants were evaluated under identical conditions (Fig. 3). Reduction by ADH112 of 2-ethylhexyl (*E*)-3-(4-acetylphenyl)acrylate was monitored over time (cf. Fig. 2c). Comparisons between Tween 60 (orange line), TPGS-1000 (light gray line), solutol HS15 (yellow line), SPGS-550-M (aka “Nok”; pink line), PTS-600 (green line), Triton X-100 (dark blue line), labrasol (brown line), and Brij 30 (red line) were compared to the initial results obtained in buffer vs. those from 2 wt % TPGS-750-M/buffer. In line with expectations, given that each surfactant is capable of serving in a similar capacity, all led to superior results compared to that in buffer alone, again lending further credence to the proposed “reservoir” effect. None, however, outperformed use of 2 wt % TPGS-750-M.

### Stability studies

**Fig. 4:**
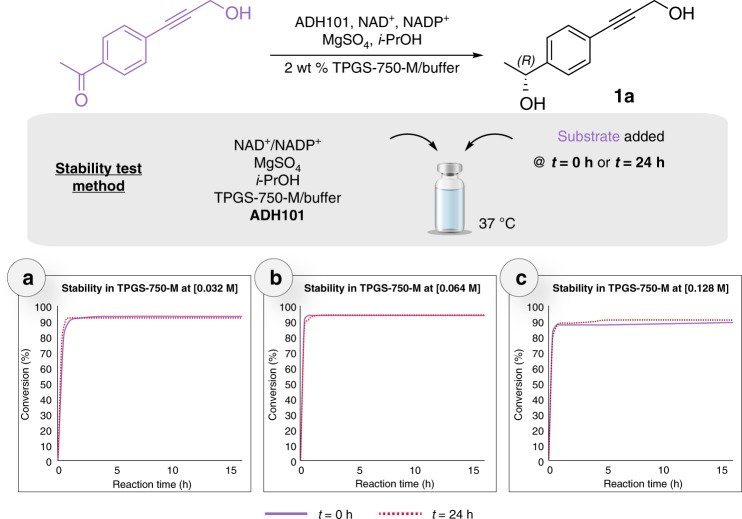
Stability of ADH101 in aq. TPGS-750-M. The three graphs correspond to the monitoring of the reduction of 1-(4-(3-hydroxyprop-1-yn-1-yl)phenyl)ethan-1-one to **1a** at different concentrations relative to substrate (**a** [0.032 M], **b** [0.064 M], **c** [0.124 M]). For each graph, two experiments were run: one with no enzyme incubation (purple line), and one with a 24 h incubation period in the aqueous medium (red dashed line). Conditions: 1-(4-(3-hydroxyprop-1-yn-1-yl)phenyl)ethan-1-one (case **a**: 20 mg, case **b**: 40 mg, case **c**: 80 mg) was added (at *t* = 0 or *t* = 24 h) to a solution of ADH101 (20 mg) MgSO_4_ (0.8 mg), NAD^+^ (2.6 mg), NADP^+^ (2.4 mg) and *i*-PrOH (0.4 mL) in 2 wt % TPGS-750-M/buffer (3.2 mL) at 37 °C for 24 h. Source data are provided as Source Data File

The stability of the enzyme ADH 101 in the presence of TPGS-750-M over time was further evaluated, as surfactants, on occasion, have been reported to denature enzymes^43^. Reduction of 1-(4-(3-hydroxyprop-1-yn-1-yl)phenyl)ethan-1-one at 0.032 M was carried out, and after 24 h of incubation (Fig. 4a), there was no apparent impact on enzymatic activity. Even at higher substrate concentrations (Fig. 4b, c—[0.064 M] and [0.128 M], respectively), where only incomplete conversion is achieved (plateaued at 90%), no loss of activity was observed after 24 h of incubation.

Circular dichroism (CD) analyses further confirmed that the enzyme structure is not compromised in the presence of surfactant (see Supplementary Information—Supplementary Fig. 3). ^1^H NMR spectra, taken with and without surfactant present, overlap perfectly, especially in the amide and CH_3_ regions where no strong interaction is detected. The absence of signal modification in the CH_3_ region tends to exclude the hypothesis that the enzyme is inside the micelle (see Supplementary Information—Supplementary Figs. 4–6). The micelles also remain undisturbed by the presence of the protein as the same average diameter was detected by dynamic light scattering analyses of a solution of TPGS-750-M/buffer in the presence of, or in the absence of, ADH101 (see Supplementary Information—Fig. 7).

### Sequential tandem catalysis

Following optimization of experimental conditions, sequential two-step, one-pot processes were investigated. Cross-coupling reactions, as well as hydration of alkynes, were directly followed by reduction of each ketone-containing product by ADH in the same pot. While some early experimental challenges had to be overcome (vide infra), second-stage enzymatic reductions were found to be compatible with the presence of metals, such as palladium, copper, rhodium, iron and gold, along with various salts that may have been generated from a prior reaction. While most metal-mediated reactions performed in TPGS-750-M/H_2_O involve substrate concentrations of 0.5 M, such conditions are not compatible with the reductases being used, leading to incomplete conversions. Therefore, dilution of the medium was required prior to addition of the alcohol dehydrogenase, from [0.500 M] to [0.056 M] (dilution by ≈10 of the ketone), while the temperature was adjusted to 37 °C. Despite a claimed optimal pH of 7, we observed that the enzyme can tolerate a broader pH range (from 4 to 7). Cross-coupling reactions in water that involve basic conditions, however, are performed at elevated pH and hence, an adjustment to neutral-to-acidic conditions is required prior to the biocatalytic step. Tests where addition of an enzyme at a higher pH followed by pH lowering to 7 led to no reduction.

**Fig. 5:**
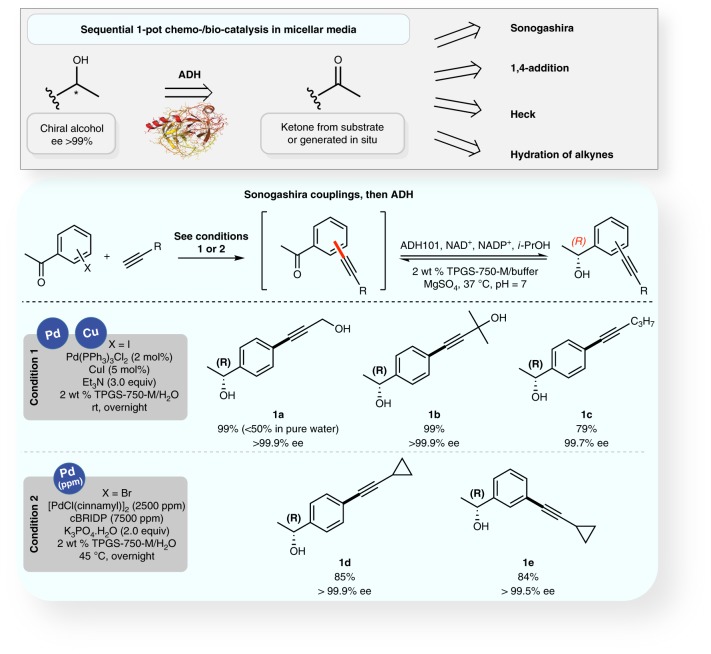
Sequential Sonogashira coupling/ADH ketone reduction. Sonogashira cross-coupling reactions were followed by asymmetric reduction of the ketone present in each product, mediated by ADH, all in a single pot, enabled by TPGS-750-M

The one-pot Pd-catalyzed Sonogashira cross-coupling of an acetoaryl iodide or bromide followed by a biocatalytic ketone reduction was initially studied. Variations in experimental conditions mainly focused on the tolerance of the enzyme to the presence of copper and palladium, although only ppm levels of Pd are required for these reactions (Fig. 5). Upon generation of an arylated product alkyne, the pH was decreased to seven, followed by addition of ADH and the co-factor. Asymmetric reduction of the ketone present in each afforded the corresponding nonracemic alcohols with excellent enantioselectivities (>99.5% ee). The overall yields of the two-step processes are identical to the yield characteristic of the first step alone, attesting to the impressive efficiency of the enzymatic process independent of its modified environment. The background sequence run in the absence of TPGS-750-M leading to product **1a** led to a significantly decreased overall yield (less than 50%), attesting of the importance of the surfactant in the tandem process.

**Fig. 6:**
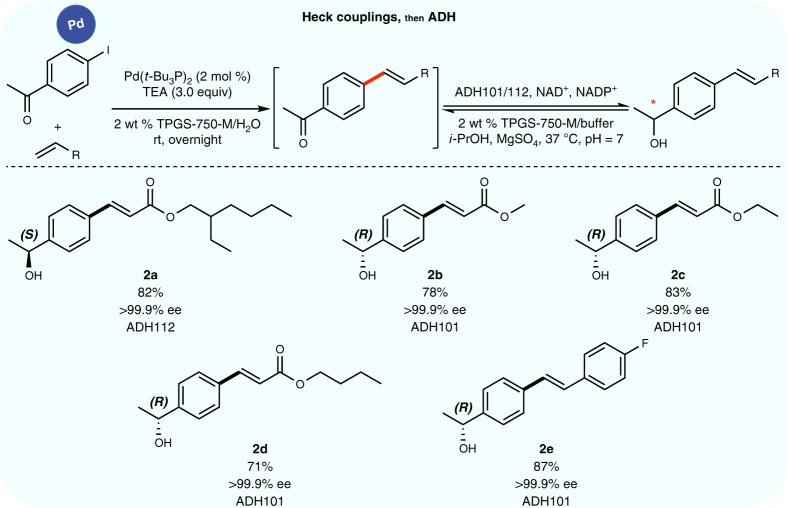
Sequential Heck cross-coupling/ADH ketone reduction. Heck cross-coupling reactions were followed by asymmetric reduction of the ketone present in each product, mediated by ADH, in aqueous TPGS-750-M

**Fig. 7:**
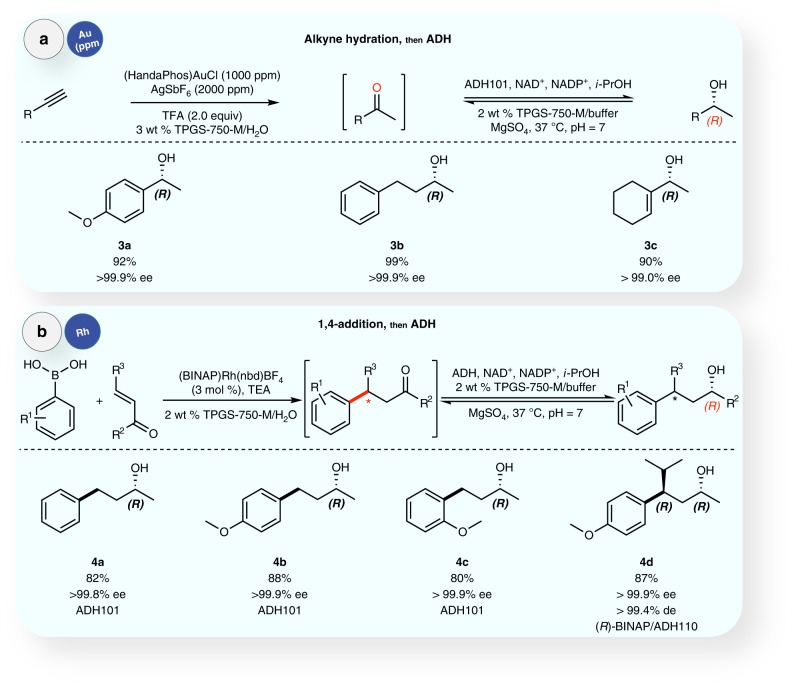
Transition metal-catalyzed reactions followed by ADH ketone reductions. Plot **a** describes alkyne hydration reactions followed by the asymmetric reductions of the ketones generated by ADH101; plot **b** describes 1,4-addition reactions, followed by ADH-mediated asymmetric reductions of the ketone present in each product, enabled by aqueous TPGS-750-M

The enzyme also performed quite well in combination with Heck reactions (Fig. 6)^45^. Both ADH101 and ADH112, being (*R*)- and (*S*)-selective, respectively, towards an acetophenone led to enantiomeric products (**2b**−**e** vs. **2a**) with essentially perfect fidelity (>99.9% ee). Notably, even 2 mol% of Pd (i.e., 20,000 ppm) is well tolerated by the enzyme. Switching to ppm-level gold catalysis, generation of ketones in situ via hydration of alkynes^46^ followed by their reduction to the corresponding nonracemic alcohols led to excellent results as well (Fig. 7a). Lastly, exposure of the same enzymatic system to rhodium, used catalytically to effect an initial 1,4-addition^47^ of a boronic acid to an enone, was of no consequence as ketone reduction took place uneventfully (Fig. 7b). Interestingly, a match/mismatch effect was observed with compound **4d**. Indeed, when the first step is conducted with *rac*-BINAP, ADH110 transformed only the 4(*R)*-enantiomer, leaving the 4(*S)*-product intact. ADH105, (*S*)-selective toward acetophenone, performed the reaction only on the 4(*S*)-enantiomer. While the reaction conducted with (*R)*-BINAP led to 97.5% ee (*R*) for the first step, the final product (in a one-pot process) was obtained with >99.9% ee and >99.4% de, with 2.5% of the opposite enantiomer remaining.

**Fig. 8:**
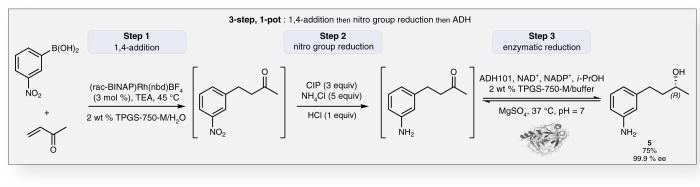
Sequential reactions combining chemo-catalysis and bio-catalysis. A 1,4-addition reaction was followed by a nitro group reduction, and then an asymmetric reduction of the keto-product mediated by ADH101, all in one-pot and enabled by aqueous TPGS-750-M. The enantiomeric excess (ee) was determined by chiral HPLC after *N*-acetylation of **5**

To further illustrate the potential for these tandem events in synthesis, the one-pot process could be smoothly extended to three-steps, the product (overall 75%, 99.9% ee) resulting from a sequential 1,4-addition, followed by a nitro group reduction and ultimately, reduction by ADH101 (Fig. 8). The enzyme fully reduced the ketone in less than 2 h, notwithstanding the presence of Rh, Fe, and other metal salts. The significant robustness having been added to the biocatalytic step by the presence of TPGS-750-M in the medium opens the door to additional, potentially even longer sequences^48^, all in water under very mild conditions, as these enzymes appear to tolerate a mixture of residual catalysts in the pot from previous steps.

### Industrial application

**Fig. 9:**
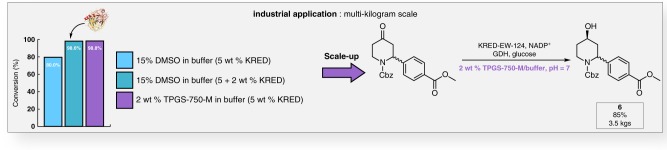
Industrial application on a 8.2 mol scale. Comparison on gram scale of the KRED-EW-124 activity in 15% DMSO vs. TPGS-750-M in buffer, along with scale-up of the synthesis of **6** on a multi-kilogram scale. Source data are provided within the Source Data File

The beneficial impact of TPGS-750-M on enzymatic activity was applied to an industrial scale process with the multi-kilogram synthesis of the functionalized chiral 4-piperidinol **6** (Fig. 9). The reaction, performed in TPGS-750-M/H_2_O, was compared to that run using 15% DMSO/H_2_O, both in a phosphate buffer at 40 °C using 5% KRED-EW-124. In aqueous TPGS-750-M the reaction reached 98% conversion after 25 h, while in DMSO/H_2_O it stopped at 80%; an additional 2 wt % of enzyme was necessary to access the same 98% level of conversion. Extensive optimization also led to an increase of more than 50% in the concentration in compound **6** in the reaction mixture, thus resulting in a substantial increase in productivity. Indeed, compared to an optimized process in DMSO, the approach using TPGS-750-M/H_2_O leads to a reduction of the Process Mass Intensity (PMI) of more than 32%, and a productivity increase of greater than 40%.

Importantly, evaluation of ADH101 on 4-phenylbut-3-en-2-one (Fig. 2d), with 10 v/v % of THF in the reaction mixture, commonly used as cosolvent in industrial applications^49,50^, showed no decrease in activity. Likewise, the presence of high concentrations of salts (e.g., 2 M NaCl)^51^ did not alter the outcome. Additionally, the water-soluble surfactant is not extracted with the product and any residual traces can be easily separated by a filtration through a small pad of silica.

### Conclusions

Aqueous solutions containing micelles derived from the tailor-made surfactant TPGS-750-M are shown to be not only fully compatible with alcohol dehydrogenase, but also responsible for enhancing enzymatic activity, especially towards highly lipophilic substrates. While no direct interactions are detected between both nanoreactors in the aqueous medium (i.e., micelles and enzymes), the former appear to serve as reservoirs for substrates (and presumably catalysts), thereby moderating the degree of enzyme saturation. Chemo- and bio-catalysis can now be combined in a one-pot sequential fashion, both run under mild conditions, leading to virtually stereopure products of greater complexities. Use of the surfactant also clearly increases the productivity associated with large-scale applications. These results suggest that similar outcomes might be anticipated using other types of enzymatic processes based on readily available kits, in addition to sequences that take advantage of both types of reaction media that exist, by definition, under micellar catalysis conditions (e.g., a coupling reaction followed by two successive enzymatic reactions). These approaches are under active investigation in our laboratories and will be reported in due course.

## Methods

### Sonogashira—Condition 1

To a dried 1 dr vial were added, under an argon atmosphere, Pd(PPh_3_)_3_Cl_2_ (2.8 mg, 2 mol%), CuI (1.9 mg, 5 mol%), aryl iodide (1 equiv, 0.2 mmol), alkyne (1.5 equiv, 0.3 mmol) and Et_3_N (84 µL, 3 equiv). The vial was capped with a rubber septum. A 2 wt % TPGS-750-M/H_2_O (0.4 mL) was added. The reaction was stirred at room temperature (rt) under argon until completion. The pH was adjusted to 7 with a solution of HCl (1 M).

### Sonogashira—Condition 2

To a dried 1 dr vial was added, under an argon atmosphere, 100 µL of the stock solution containing [PdCl(cinnamyl)]_2_ (2.6 mg, 2500 ppm) and cBRIDP (10.6 mg, 7500 ppm) in dry THF (2 mL). The vial was capped with a rubber septum. THF was evaporated with a stream of argon. K_3_PO_4_·H_2_O (92.1 mg, 2 equiv, 0.4 mmol), ketone (1 equiv, 0.2 mmol) and alkyne (1.2 equiv, 0.24 mmol) were added to the vial. The vial was capped again. 2 wt % TPGS-750-M/H_2_O (0.4 mL) was added and the reaction was stirred at 45 °C under argon atmosphere for 24 h. The pH was adjusted to 7 with a solution of HCl (1 M).

### Heck

To a dried 1 dr vial were added, under an argon atmosphere, Pd(P(*t*-Bu_3_))_2_ (2.0 mg, 2 mol %), aryl iodide (1 equiv, 0.2 mmol), alkene (2 equiv, 0.4 mmol) and Et_3_N (3 equiv, 84 µL). The vial was capped with a rubber septum. A 2 wt % TPGS-750-M/H_2_O (0.4 mL) was added. The reaction was stirred at rt under argon for 24 h. The pH was adjusted to 7 with a solution of HCl (1 M).

### Hydration

To a dried 1 dr vial was added, under an argon atmosphere, 0.2 mL (1000 ppm) of a gold pre-catalyst solution containing HandaPhos-gold(I) chloride (0.8 mg, 0.001 mmol) and silver(I)hexafluoroantimonate (0.7 mg, 0.002 mmol) in dichloromethane (1 mL). The vial was capped with a rubber septum. Dichloromethane was evaporated under argon. Alkyne (0.2 mmol, 1.0 equiv) was added to the vial, followed by toluene (20 µL), a 3 wt% TPGS-750-M/H_2_O solution (0.2 mL, 1.0 M), and trifluoroacetic acid (46 mg, 0.4 mmol, 2.0 equiv). The resulting mixture was stirred at rt for 24 h. The pH was adjusted to 7 with a solution of NaOH (1 M).

### 1–4 addition

To a capped dried 1 dr vial was added, under an argon atmosphere, 0.2 mL (3 mol %) of the stock solution containing Rh(nbd)_2_BF_4_ (11.2 mg) and BINAP (18.7 mg) in dichloromethane (1 mL). Dichloromethane was evaporated under argon. Boronic acid (0.2 mmol, 1.0 equiv), followed by a 2 wt % TPGS-750-M/H_2_O solution (0.4 mL, 0.5 M) and TEA (84 µL, 0.6 mmol, 3.0 equiv) were added in succession. The reaction was stirred for 15 min until homogeneous. Vinyl ketone was then added (0.2 mmol, 1.0 equiv). The reaction was stirred 12 h at rt. No pH adjustment was required.

### ADH

The concentration was adjusted to [0.056 M] by adding 2.6 mL of a 2 wt % TPGS-750-M/buffer solution (phosphate, [0.23 M], pH = 7). MgSO_4_ (0.8 mg), NAD^+^ (2.6 mg), NADP^+^ (2.4 mg), *i*-PrOH (0.6 mL) and ADH101 or ADH112 (20.0 mg) were added in succession. The reaction was stirred at 37 °C until completion. The reaction was extracted in EtOAc. The organic layer was washed with H_2_O, dried over anhydrous MgSO_4_, filtered and concentrated under vacuum. The product was purified by flash chromatography.

## Supplementary information

Supplementary Information

Source Data

## Data Availability

All data relevant to this study are available from the corresponding author upon reasonable request. The source data underlying Figs. 2a−d, 3, 4a−c, 9 and Supplementary Figs. 1, 2, 3, 7, 9, 10, 11 and 12 are provided as a Source Data file.
