# Supplementary material for: Bridging the gap between transition metal- and bio-catalysis via aqueous micellar catalysis
Source: Nat Commun. 2019 May 15;10:2169. doi: 10.1038/s41467-019-09751-4 (PMC6520378; doi:10.1038/s41467-019-09751-4)
Supplement: Supplementary file 1 — Supplementary Information [file 41467_2019_9751_MOESM1_ESM.pdf]

Supplementary Information

Bridging the gap between transition metal- and bio-catalysis via aqueous micellar catalysis

Cortes-Clerget *et al*.

Table of Contents

Supplementary Figures 4

Supplementary Tables.. 49

Supplementary Methods.. 51

Supplementary Note 1: (R)-1-(4-iodophenyl)ethan-1-ol 61

Supplementary Note 2: (R)-1-(4-(trifluoromethyl)phenyl)ethan-1-ol... 61

Supplementary Note 3: 2-ethylbutyl (S,E)-3-(4-(1-hydroxyethyl)phenyl)acrylate.............. 61

Supplementary Note 4: (R,E)-4-phenylbut-3-en-2-ol.. 61

Supplementary Note 5: (R)-3-(4-(1-hydroxyethyl)phenyl)prop-2-yn-1-ol (1a).................... 62

Supplementary Note 6: (R)-4-(4-(1-hydroxyethyl)phenyl)-2-methylbut-3-yn-2-ol (1b)....... 62

Supplementary Note 7: (R)-1-(4-(pent-1-yn-1-yl)phenyl)ethan-1-ol (1c).......................... 63

Supplementary Note 8: (R)-1-(4-(cyclopropylethynyl)phenyl)ethan-1-ol (1d).................... 63

Supplementary Note 9: (R)-1-(3-(cyclopropylethynyl)phenyl)ethan-1-ol (1e).................... 63

Supplementary Note 10: 2-ethylhexyl (E)-3-(4-((S)-1-hydroxyethyl)phenyl)acrylate (2a)... 64

Supplementary Note 11: methyl (R,E)-3-(4-(1-hydroxyethyl)phenyl)acrylate (2b)............ 64

Supplementary Note 12: ethyl (R,E)-3-(4-(1-hydroxyethyl)phenyl)acrylate (2c)................ 64

Supplementary Note 13: butyl (R,E)-3-(4-(1-hydroxyethyl)phenyl)acrylate (2d)............... 65

Supplementary Note 14: (S,E)-1-(4-(4-fluorostyryl)phenyl)ethan-1-ol (2e)........................ 65

Supplementary Note 15: (R)-1-(4-methoxyphenyl)ethan-1-ol (3a)...................................... 66

Supplementary Note 16: (R)-4-phenylbutan-2-ol (3b).. 66

Supplementary Note 17: (R)-1-(cyclohex-1-en-1-yl)ethan-1-ol (3c).................................. 66

Supplementary Note 18: (R)-4-phenylbutan-2-ol (4a).. 66

Supplementary Note 19: (R)-4-(4-methoxyphenyl)butan-2-ol (4b)...................................... 67

Supplementary Note 20: (R)-4-(2-methoxyphenyl)butan-2-ol (4c)...................................... 67

Supplementary Note 21: (2R,4R)-4-(4-methoxyphenyl)-5-methylhexan-2-ol (4d)............. 67

Supplementary Note 22: Stereogenic center determination (4d) *68*

Supplementary Note 23: (R)-4-(3-aminophenyl)butan-2-ol (5) *68*

Supplementary Note 24: Benzyl-(R)-4-hydroxy-2-(4-(methoxycarbonyl)phenyl)piperidine-1-carboxylate (6) *68*

Supplementary references *70*

Supplementary Figures

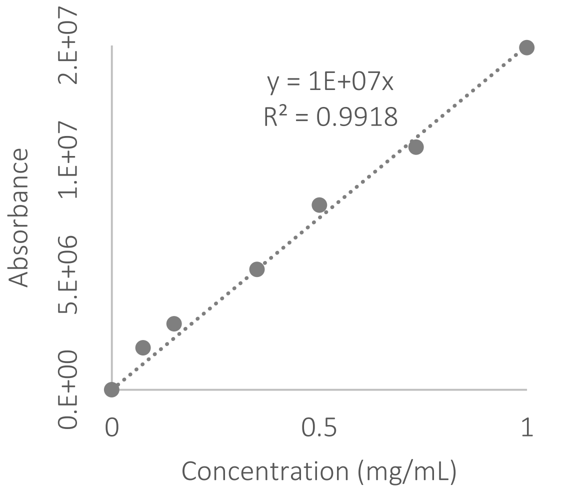

Supplementary figure 1: HPLC calibration of 4-acetophenone at 232 nm. Source data are provided as a Source Data File.

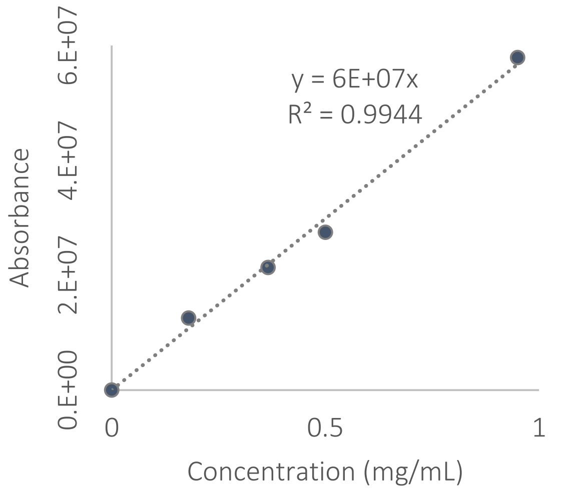

Supplementary figure 2: HPLC calibration of 1-(4-iodophenyl)-1-ethanol at 232 nm. Source data are provided as a Source Data File.

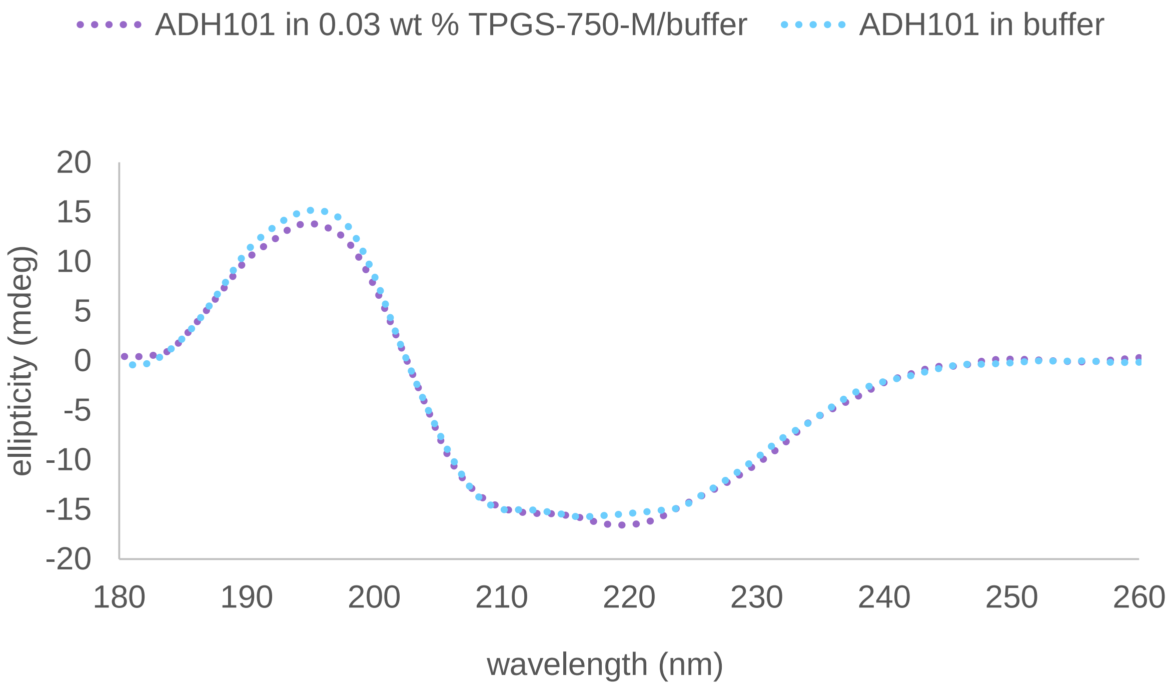

Supplementary figure 3: CD spectra of ADH101. This spectrum shows the secondary structure of ADH101 in aqueous buffer solution (blue line) and 0.03 wt % TPGS-750-M/buffer (purple line). All spectra are characteristic of proteins with both α-helical and β-sheet components, indicating the secondary structure has been conserved in presence of the surfactant. Source data are provided as a Source Data File.

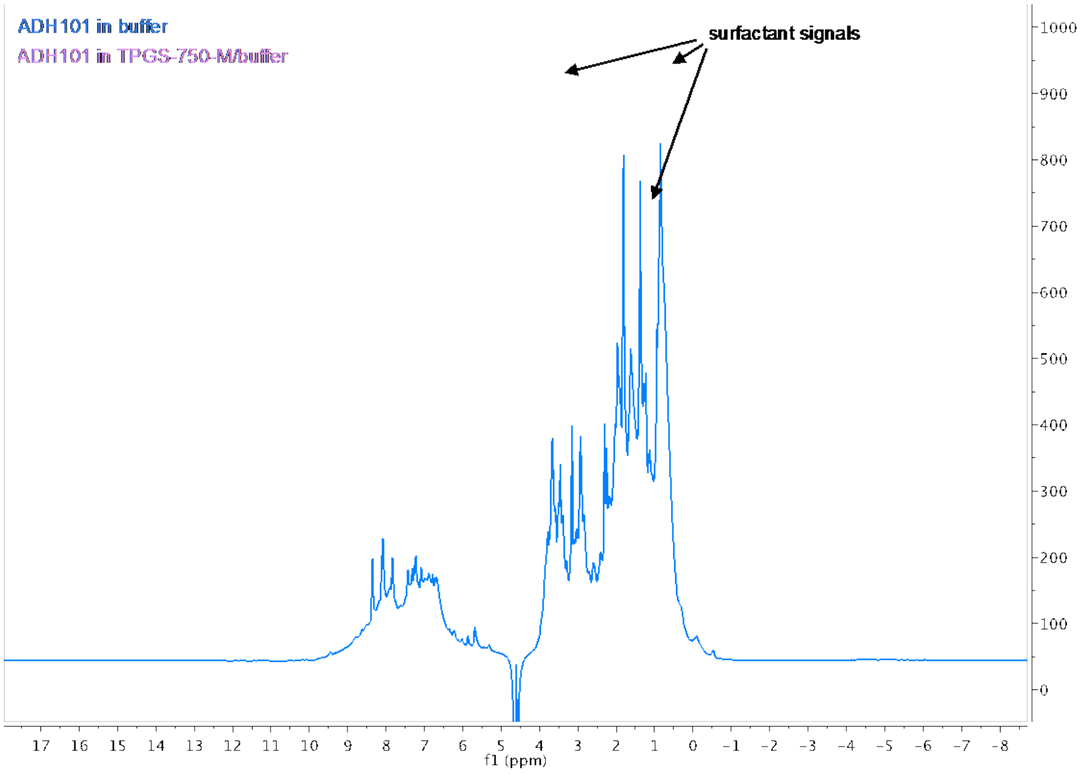

Supplementary figure 4: ^{1}H NMR spectra of ADH101. The ^{1}H NMR spectra of ADH101 has been taken in D_2O (blue line) and TPGS-750-M/D_2O (purple line). This figure shows the full spectra.

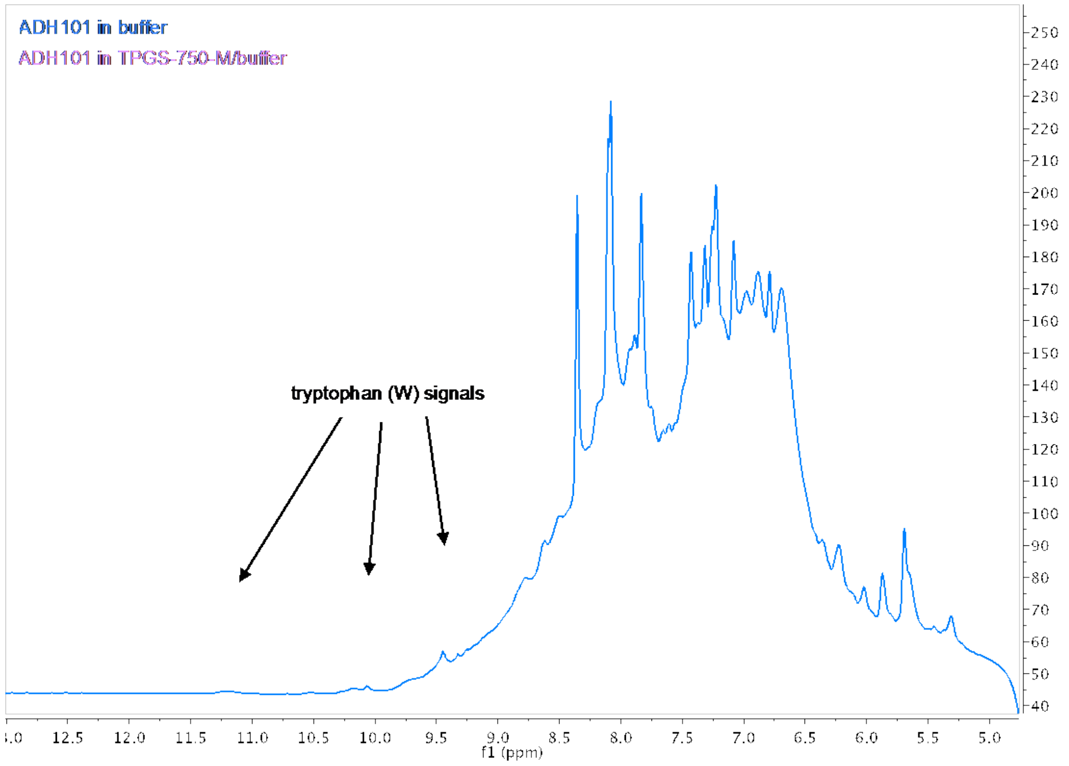

Supplementary figure 5: ^{1}H NMR spectra of ADH101. The ^{1}H NMR spectra of ADH101 has been taken in D_2O (blue line) and TPGS-750-M/D_2O (purple line). This figure shows a zoom on the amide signal section.

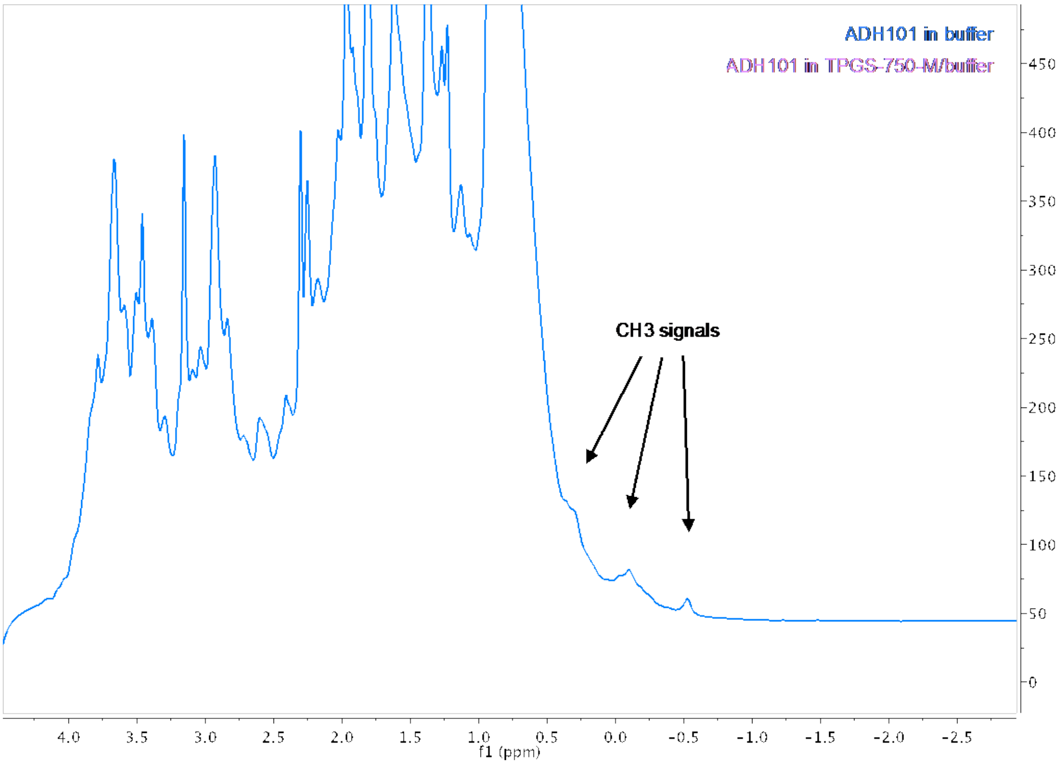

Supplementary figure 6: ^{1}H NMR spectra of ADH101. The ^{1}H NMR spectra of ADH101 has been taken in D_2O (blue line) and TPGS-750-M/D_2O (purple line). This figure shows a zoom on the hydrophobic signal section.

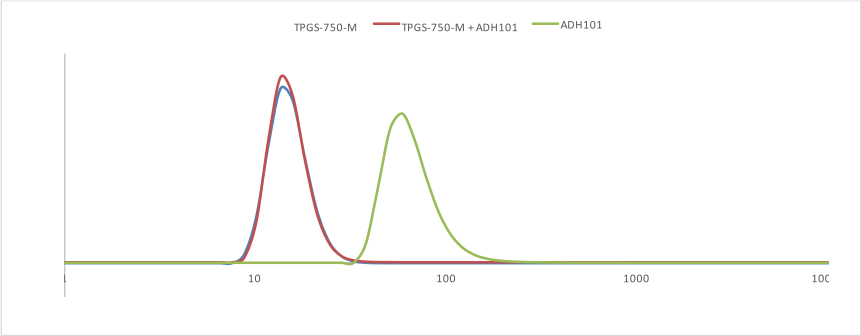

Supplementary figure 7: Dynamic Light Scattering measurement of ADH101. DLS measurements have been taken for TPGS-750-M/buffer (blue), ADH101 (green) and ADH101 in TPGS-750-M (red). Source data are provided as a Source Data File.

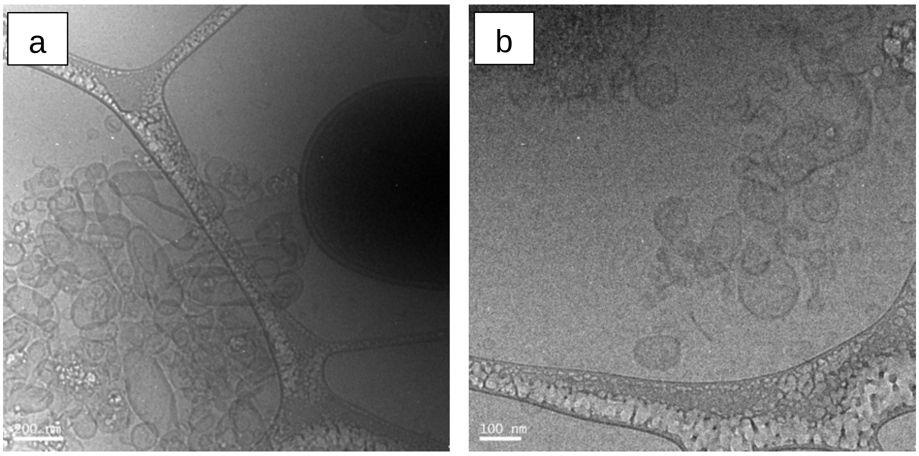

Supplementary figure 8: CryoTEM images of ADH101. The cryoTEM image has been taken with a magnification at 11.5kX (a) and 19 kX (b). The scale bar corresponds to 100 nm.

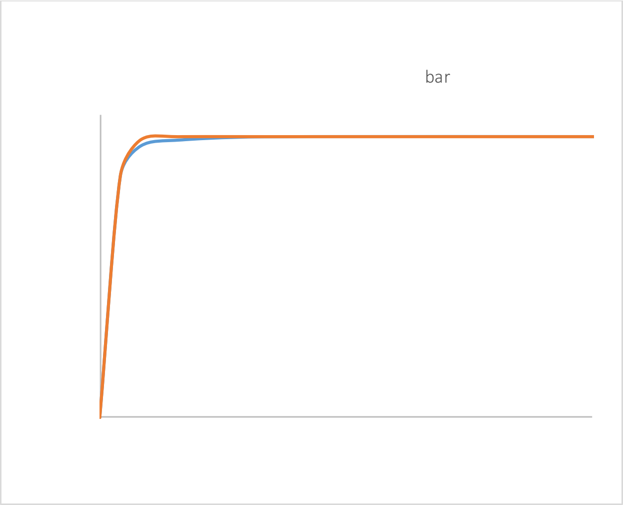

Supplementary figure. 9: Impact of the stirring method on the conversion rate. The same reaction has been performed with a shaker or a magnetic stir bar and monitored over time. The conversion has been determined by ^{1}H NMR. Source data are provided as a Source Data File.

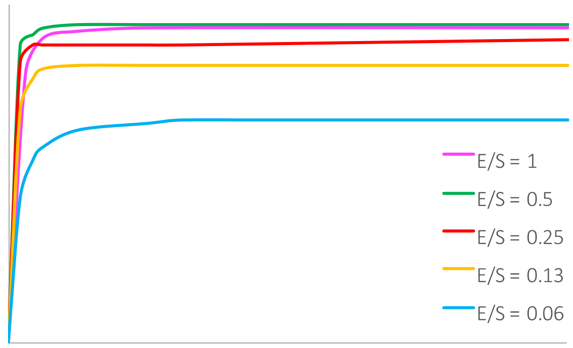

Supplementary figure 10: monitoring of the reduction reaction at different ratio enzyme/substrate. The amount of substrate has been increased and the different reactions have been monitored over time. The conversion has been determined by ^{1}H NMR. Source data are provided as a Source Data File.

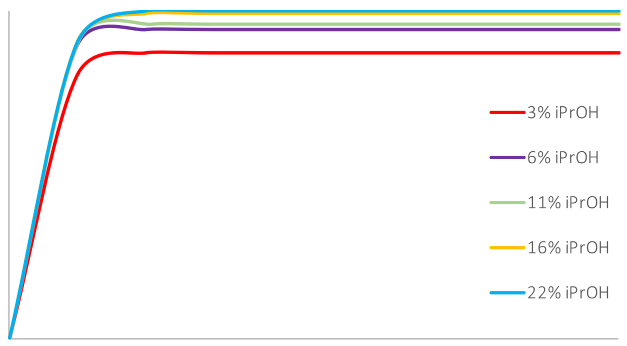

Supplementary figure 11: impact of the amount of *i*-PrOH on the enzyme activity. The volume of *i*-PrOH has been increased and the reactions have been monitored over time. The conversion has been determined by ^{1}H NMR. Source data are provided as a Source Data File.

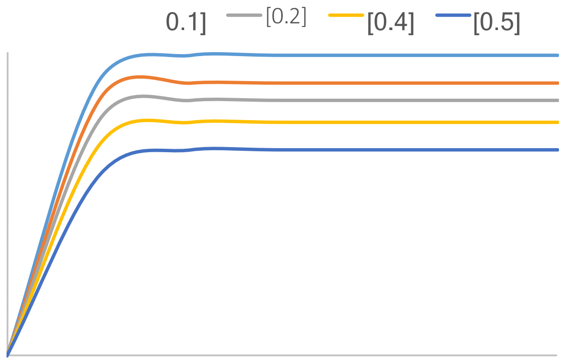

Supplementary figure 12: substrate concentration effect on the activity of ADH101. The volume of solvent has been reduced to increase the concentration and the reactions have been monitored over time. The conversion has been determined by ^{1}H NMR. Source data are provided as a Source Data File.

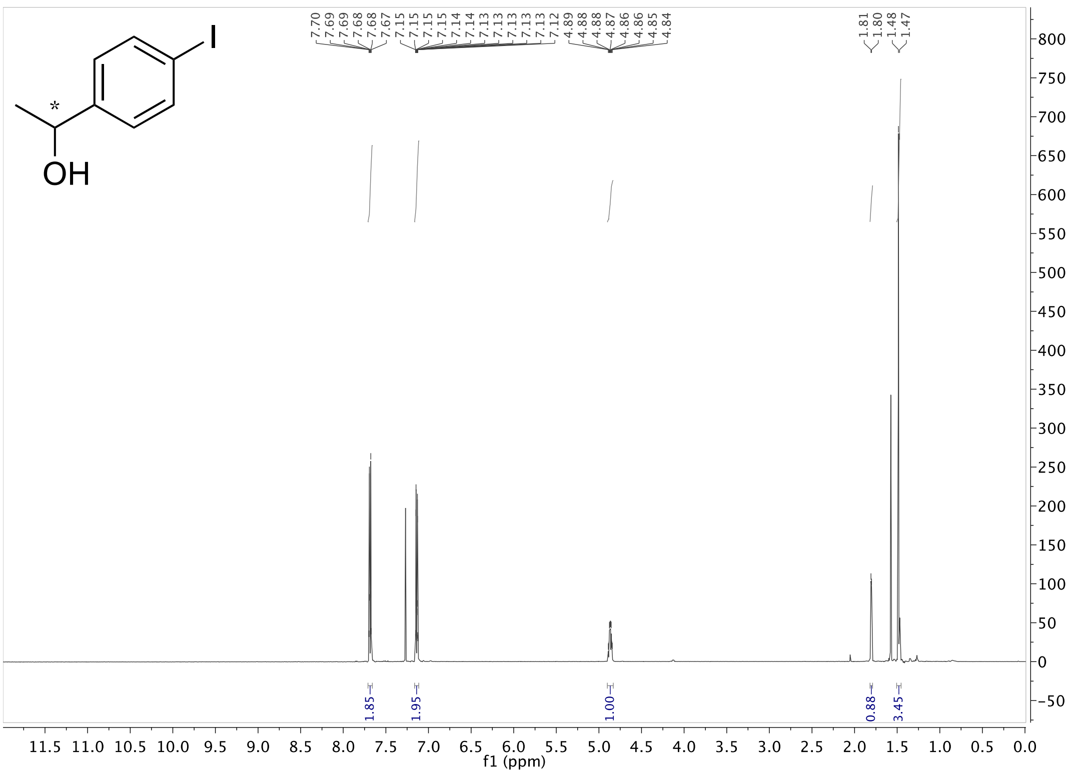

Supplementary figure 13: ^{1}H NMR spectra of 1-(4-iodophenyl)ethan-1-ol

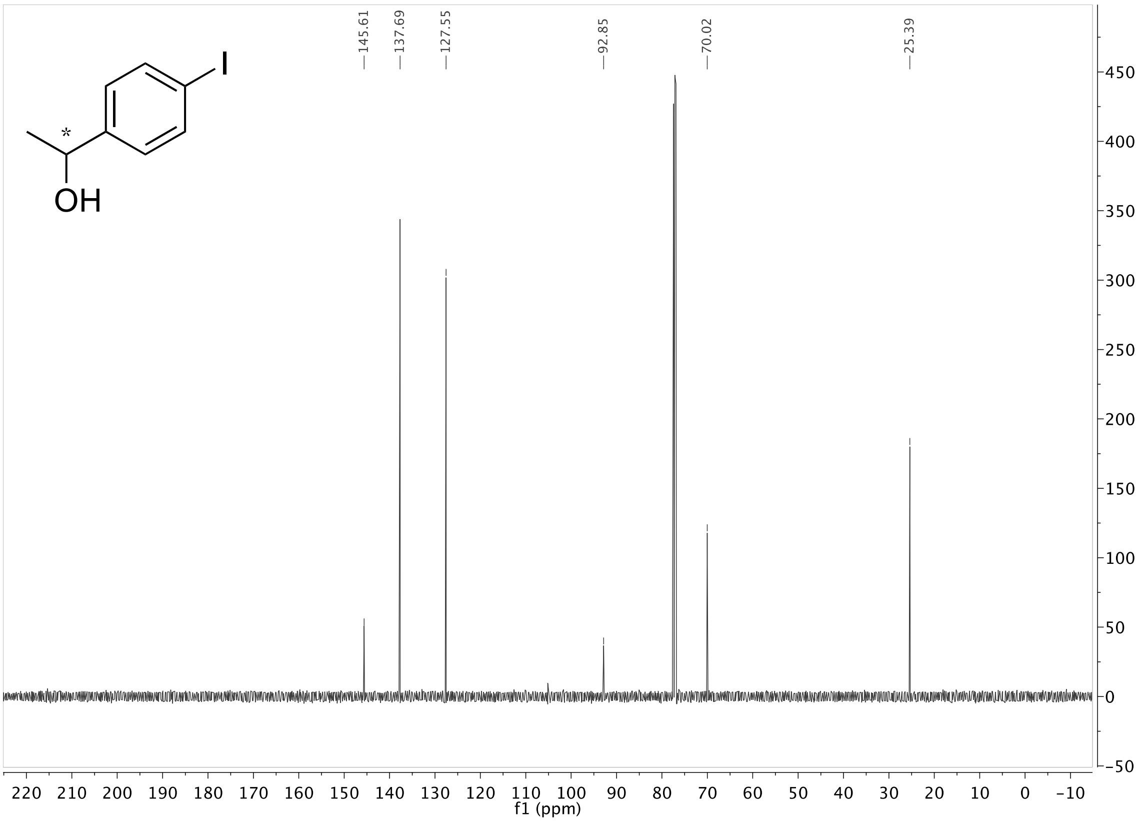

Supplementary figure 14: ^{13}C NMR spectra of 1-(4-iodophenyl)ethan-1-ol

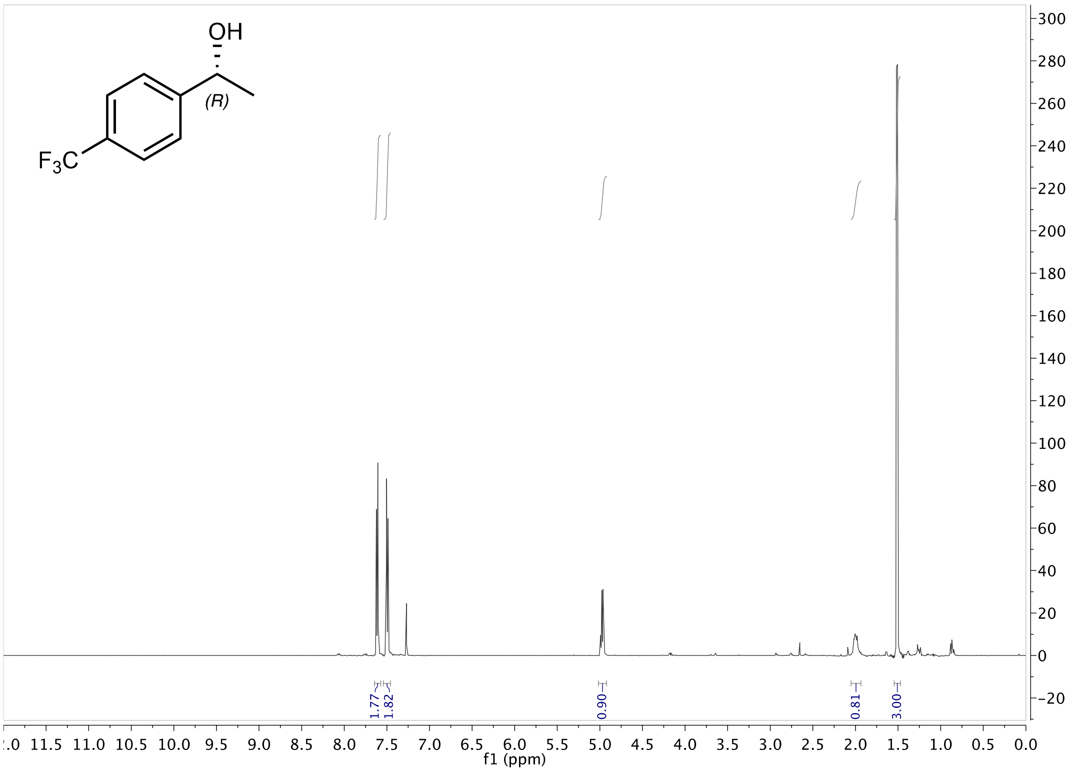

Supplementary figure 15: ^{1}H NMR spectra of (R)-1-(4-(trifluoromethyl)phenyl)ethan-1-ol

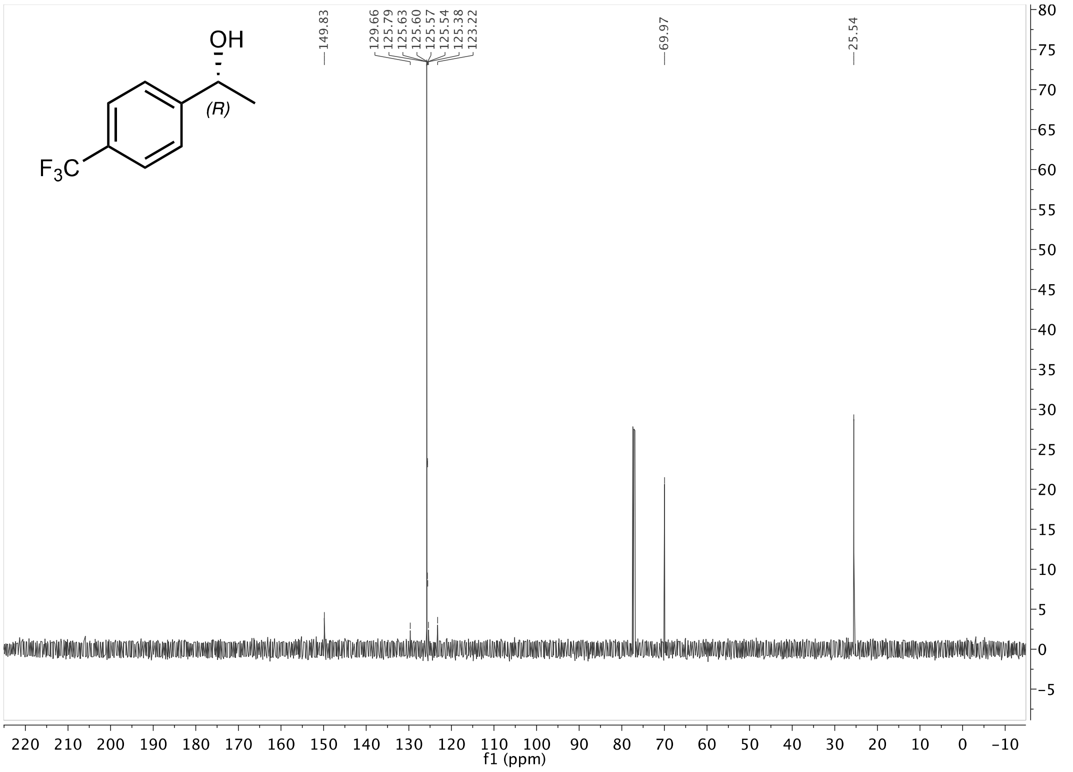

Supplementary figure 16: ^{13}C NMR spectra of (R)-1-(4-(trifluoromethyl)phenyl)ethan-1-ol

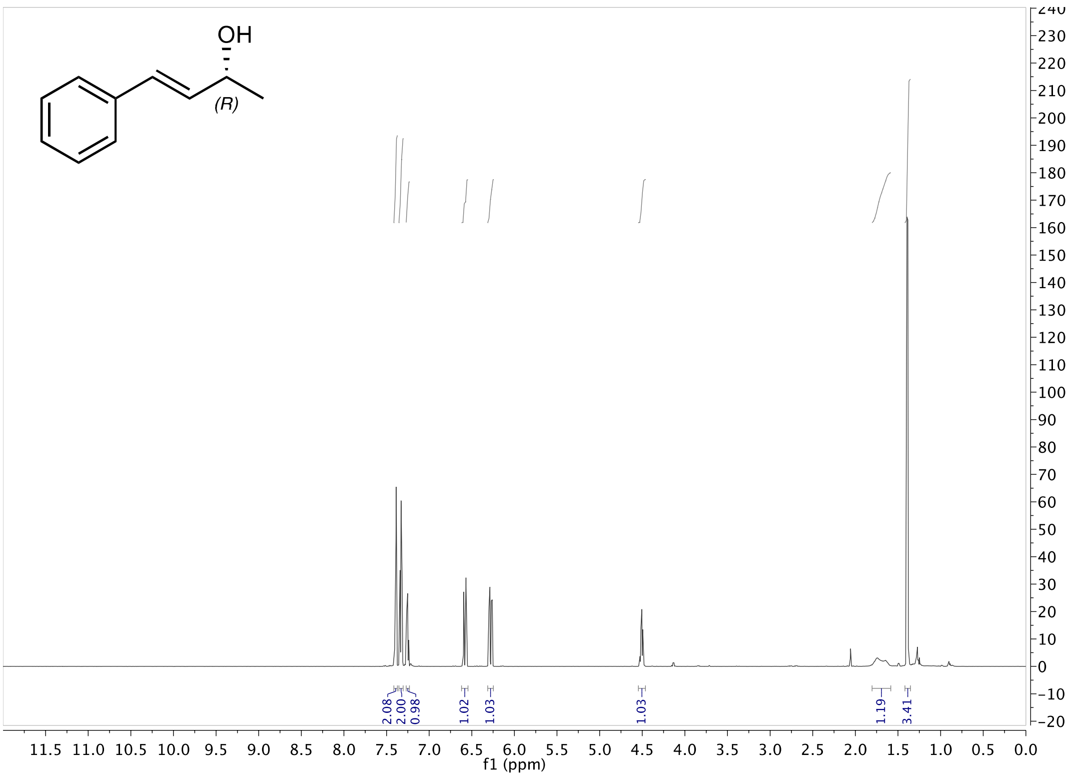

Supplementary figure 17: ^{1}H NMR spectra of (R)-4-phenyl-but-3en-2-ol

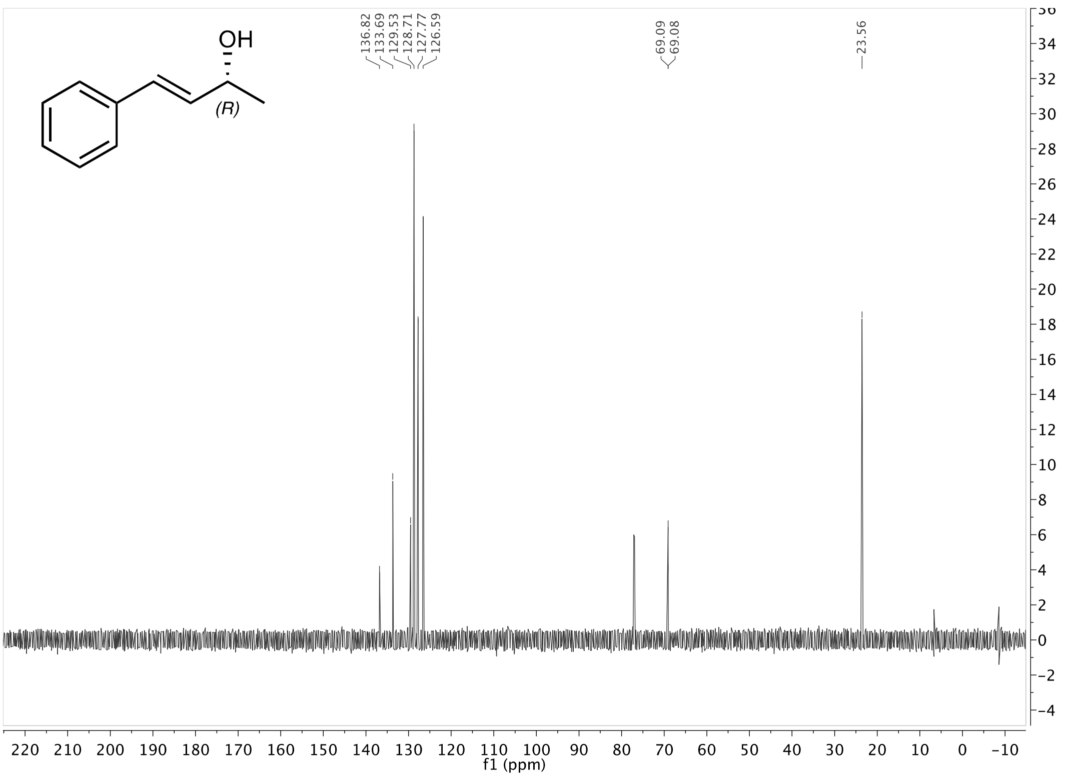

Supplementary figure 18: ^{13}C NMR spectra of (R)-4-phenyl-but-3en-2-ol

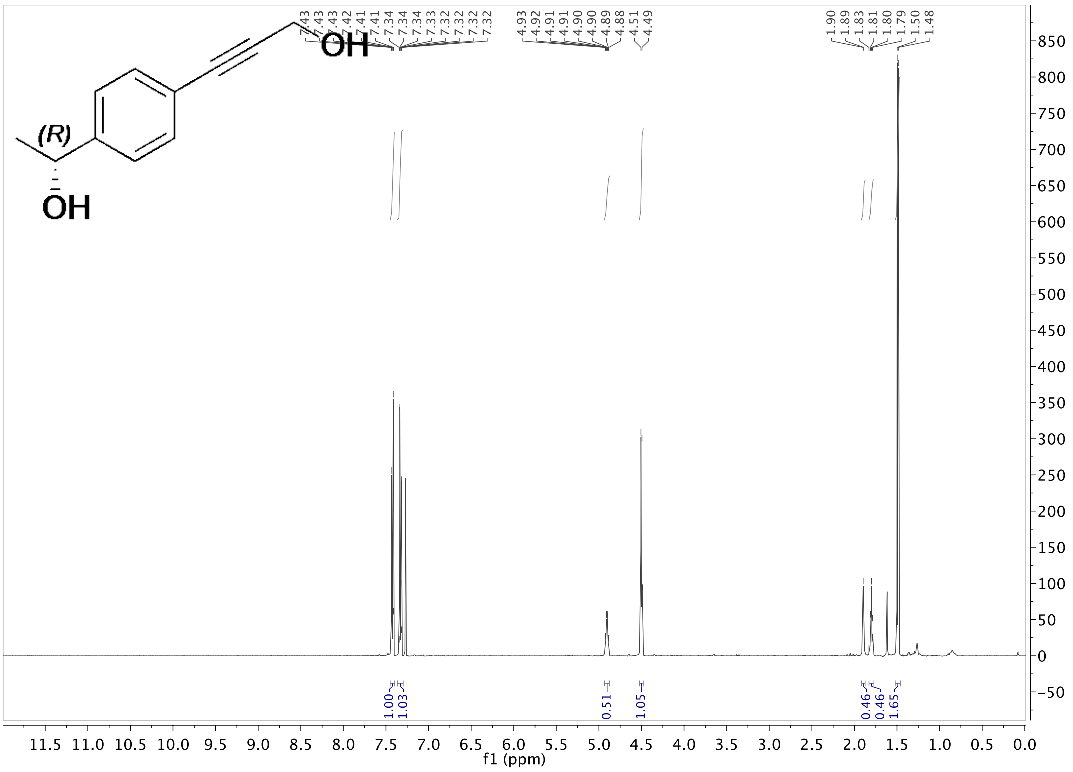

Supplementary figure 19: ^{1}H NMR spectra of 1a

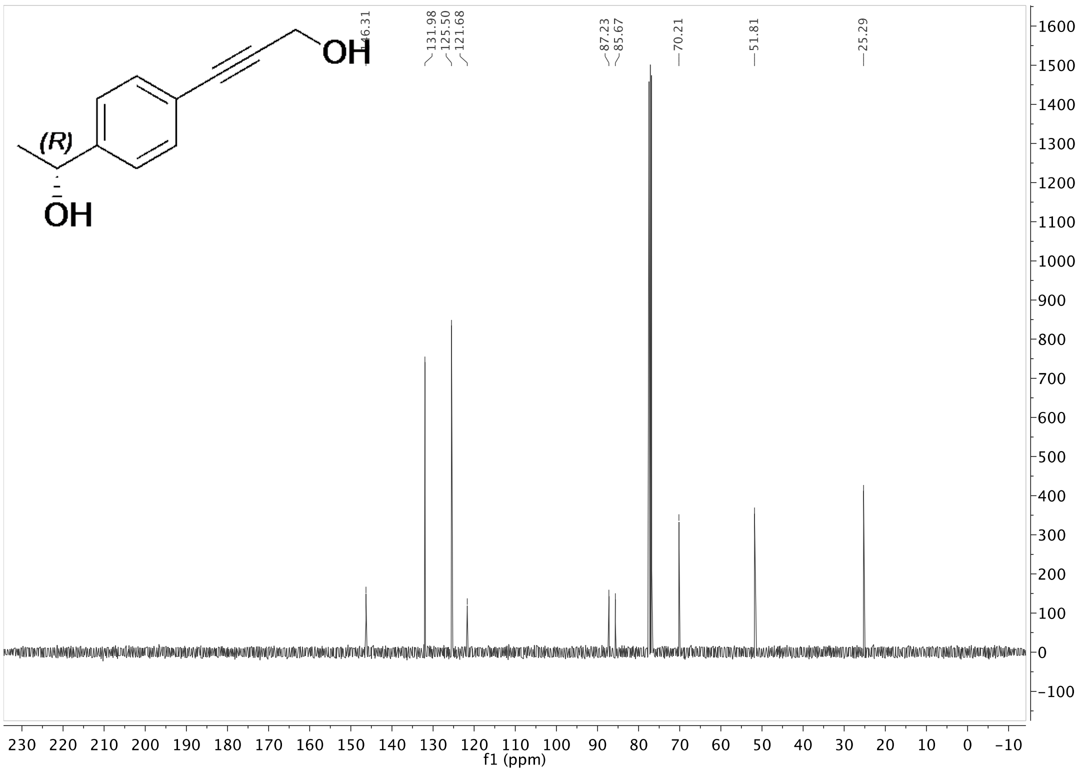

Supplementary figure 20: ^{13}C NMR spectra of 1a

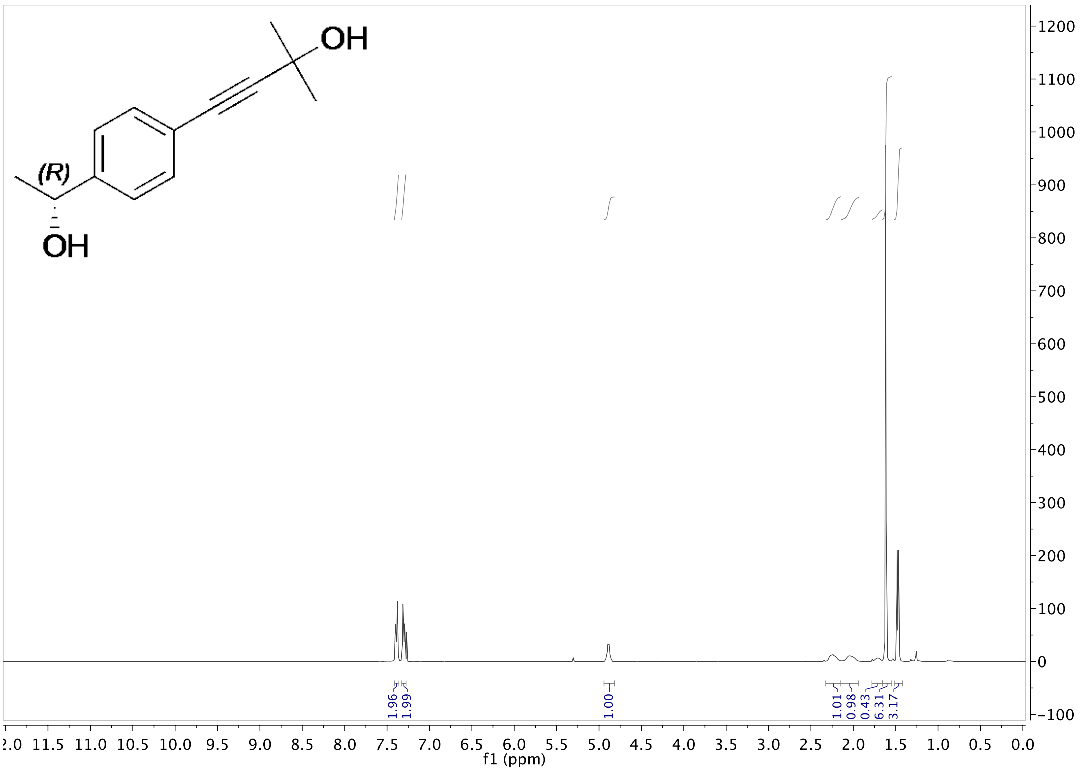

Supplementary figure 21: ^{1}H NMR spectra of 1b

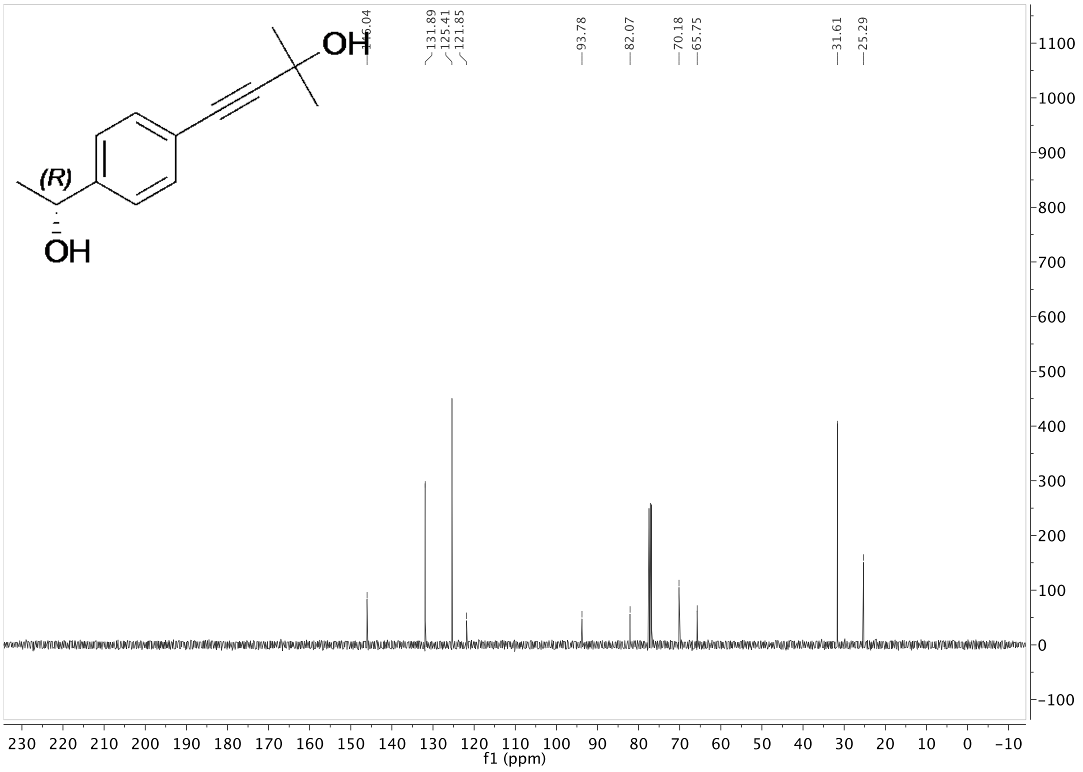

Supplementary figure 22: ^{13}C NMR spectra of 1b

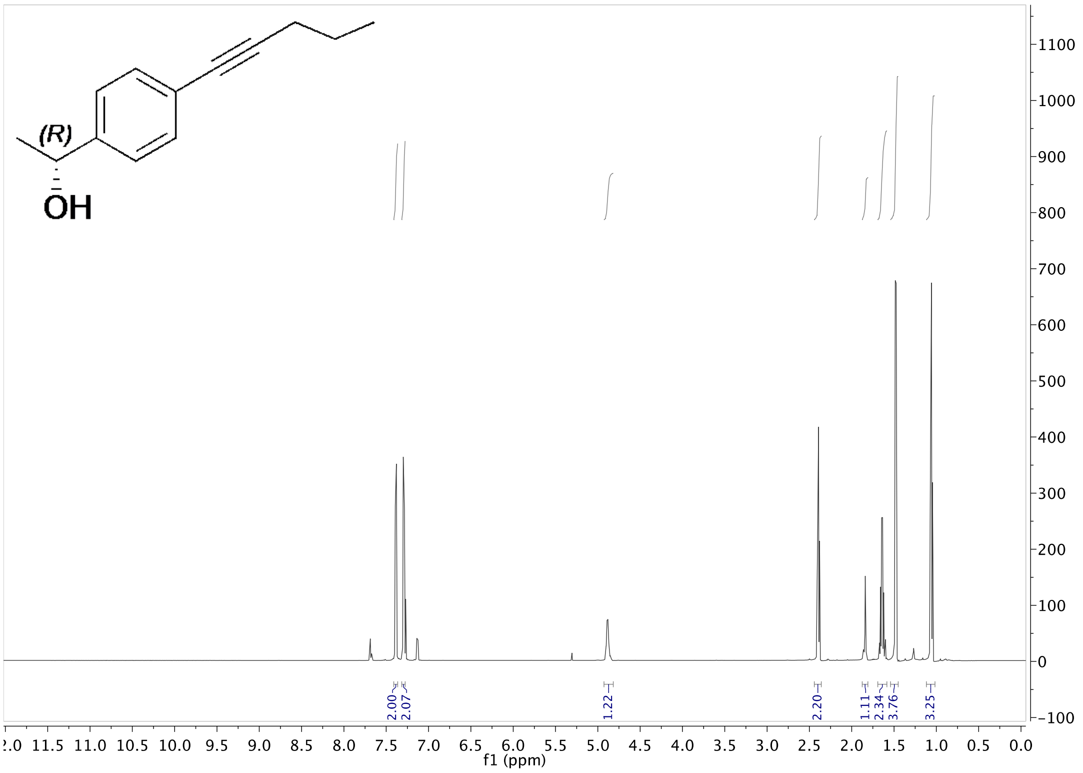

Supplementary figure 23: ^{1}H NMR spectra of 1c

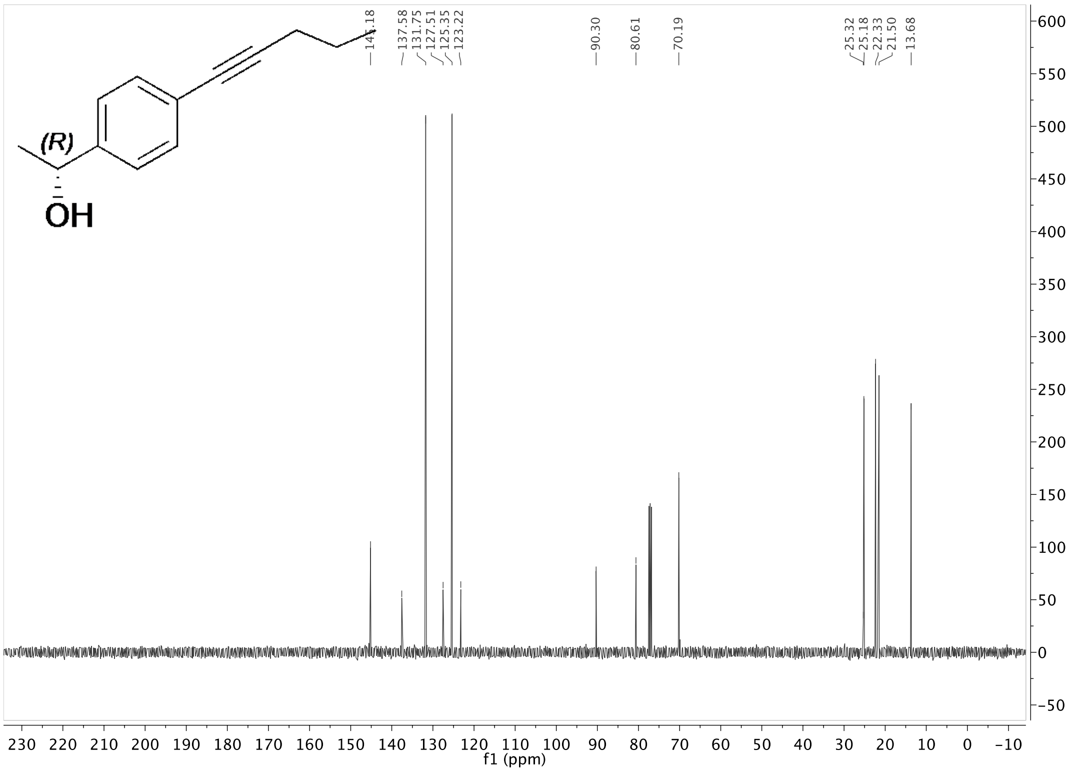

Supplementary figure 24: ^{13}C NMR spectra of 1c

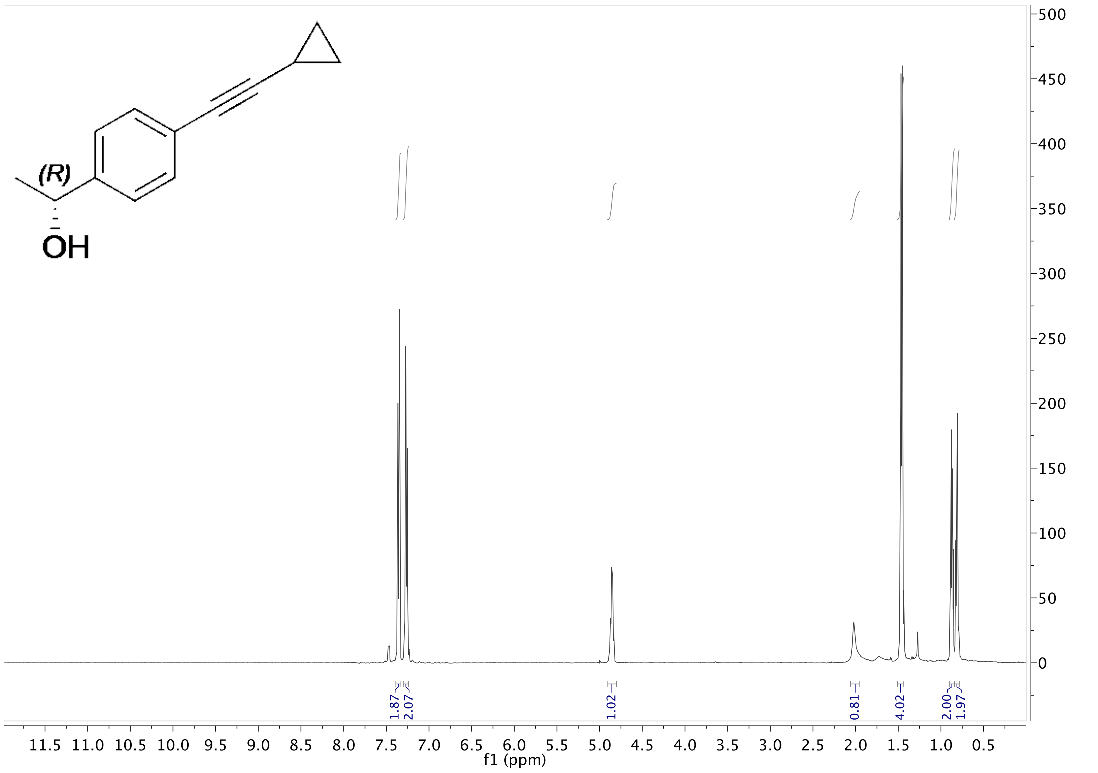

Supplementary figure 25: ^{1}H NMR spectra of 1d

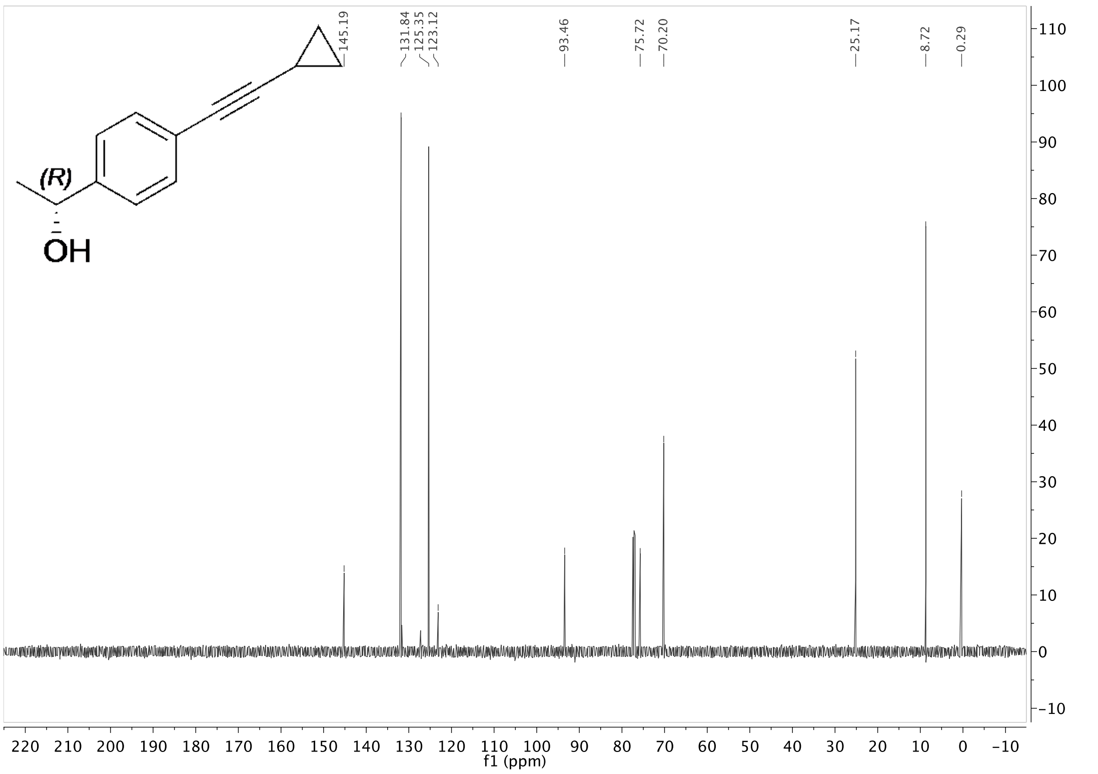

Supplementary figure 26: ^{13}C NMR spectra of 1d

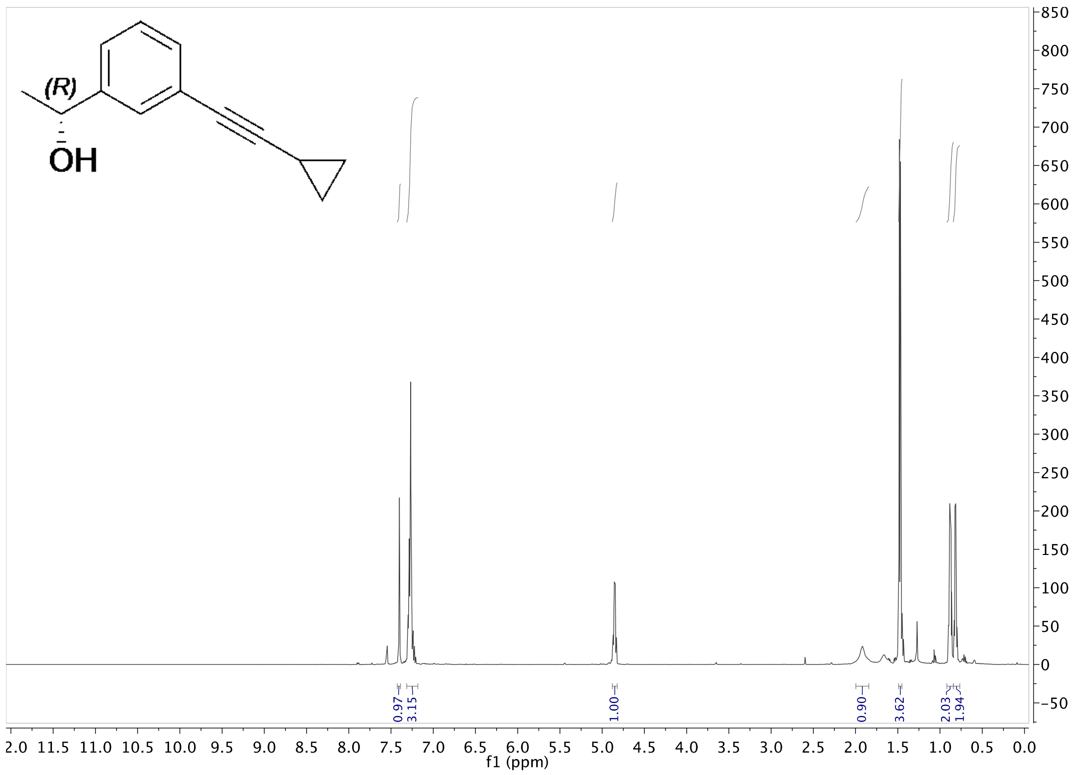

Supplementary figure 27: ^{1}H NMR spectra of 1e

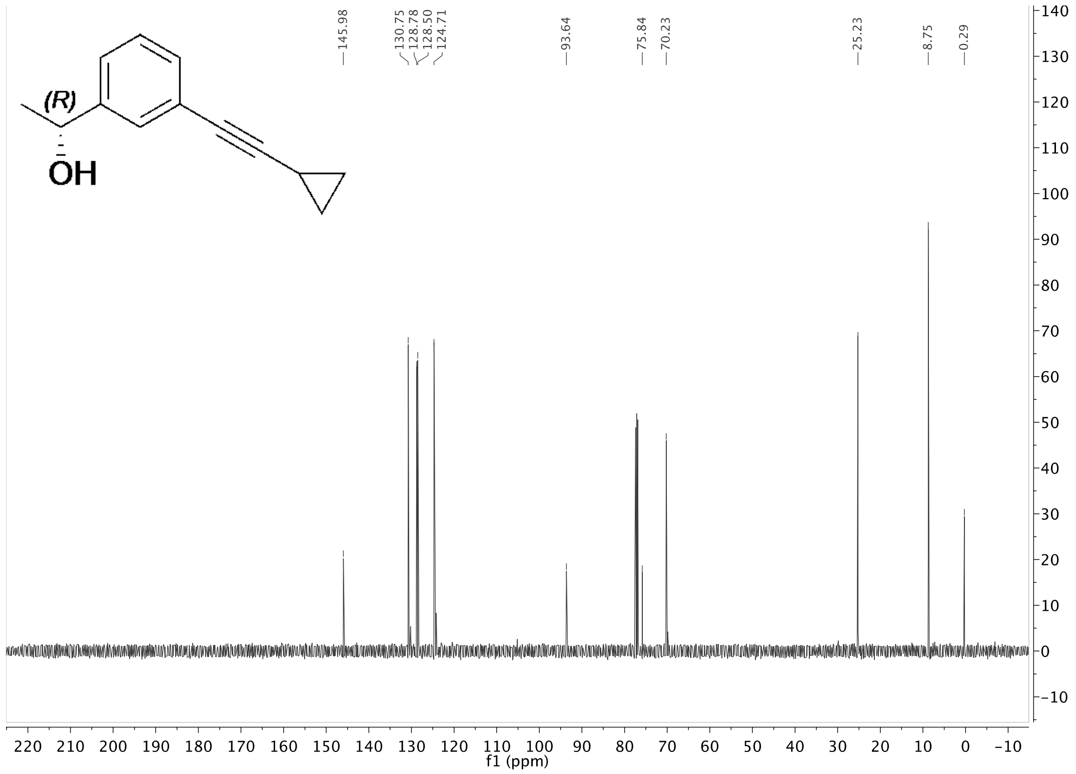

Supplementary figure 28: ^{13}C NMR spectra of 1e

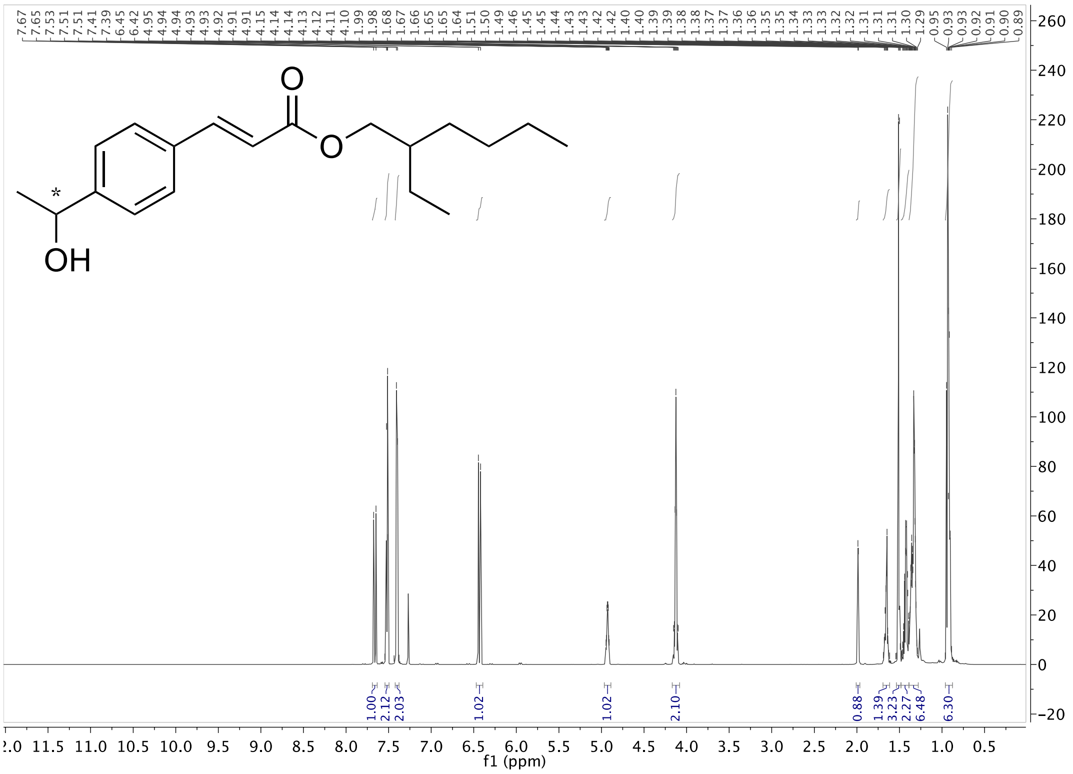

Supplementary figure 29: ^{1}H NMR spectra of 2a

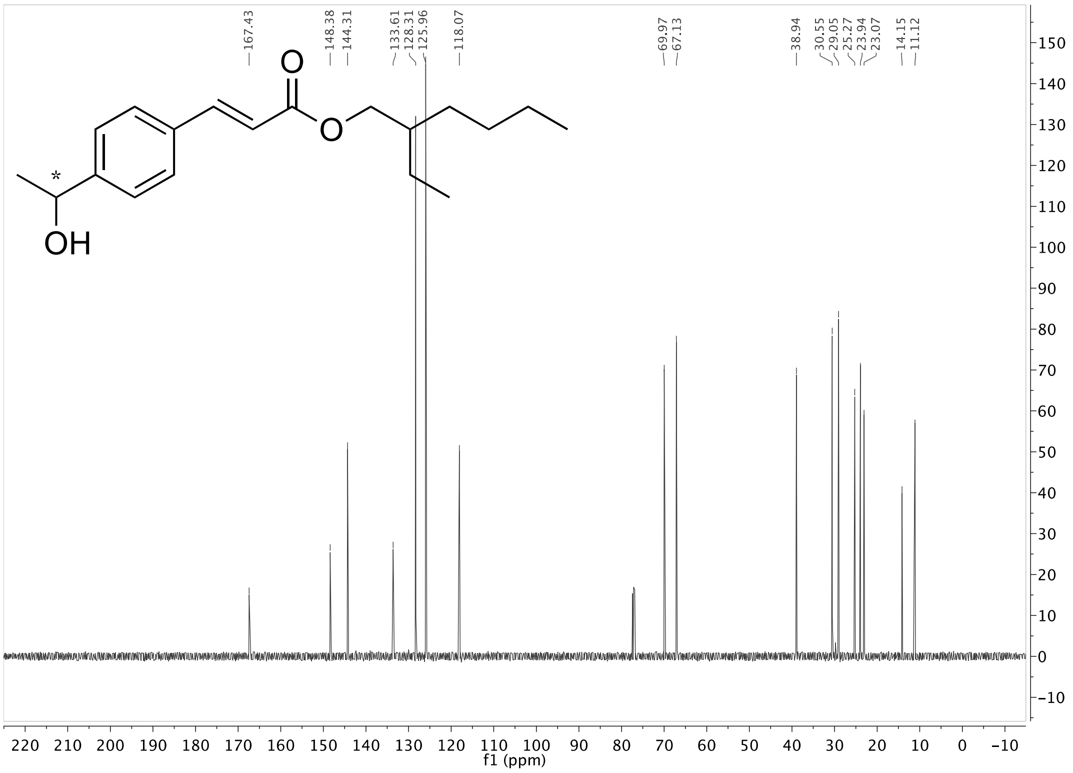

Supplementary figure 30: ^{13}C NMR spectra of 2a

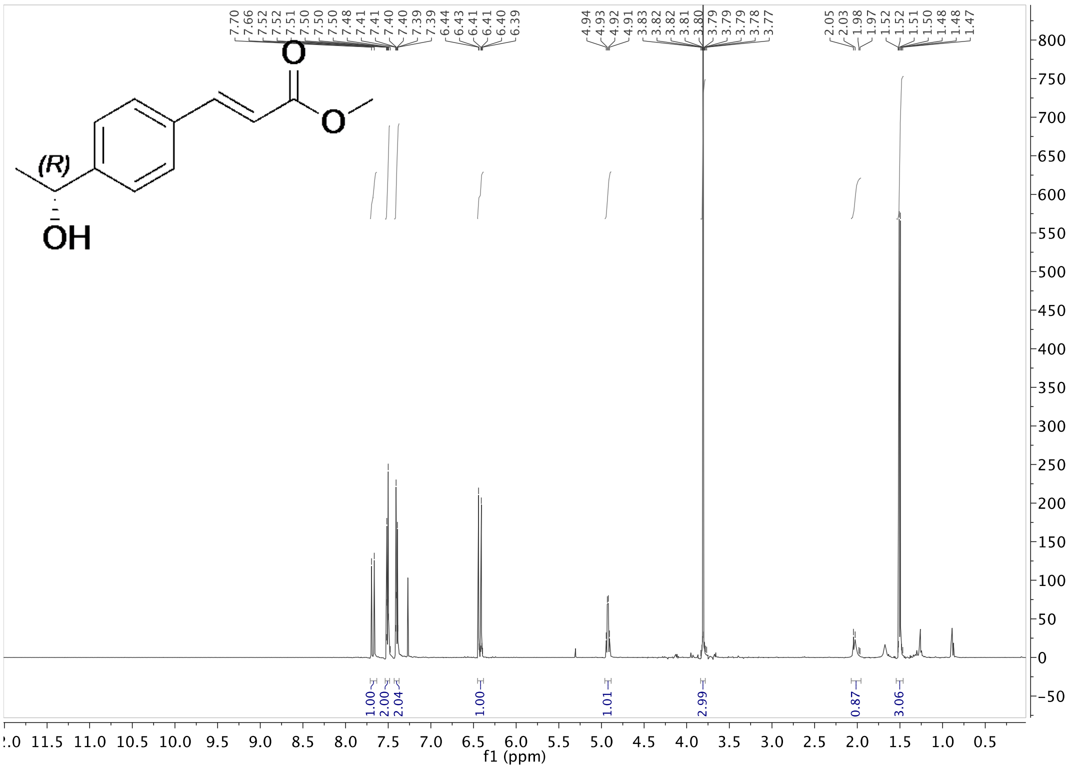

Supplementary figure 31: ^{1}H NMR spectra of 2b

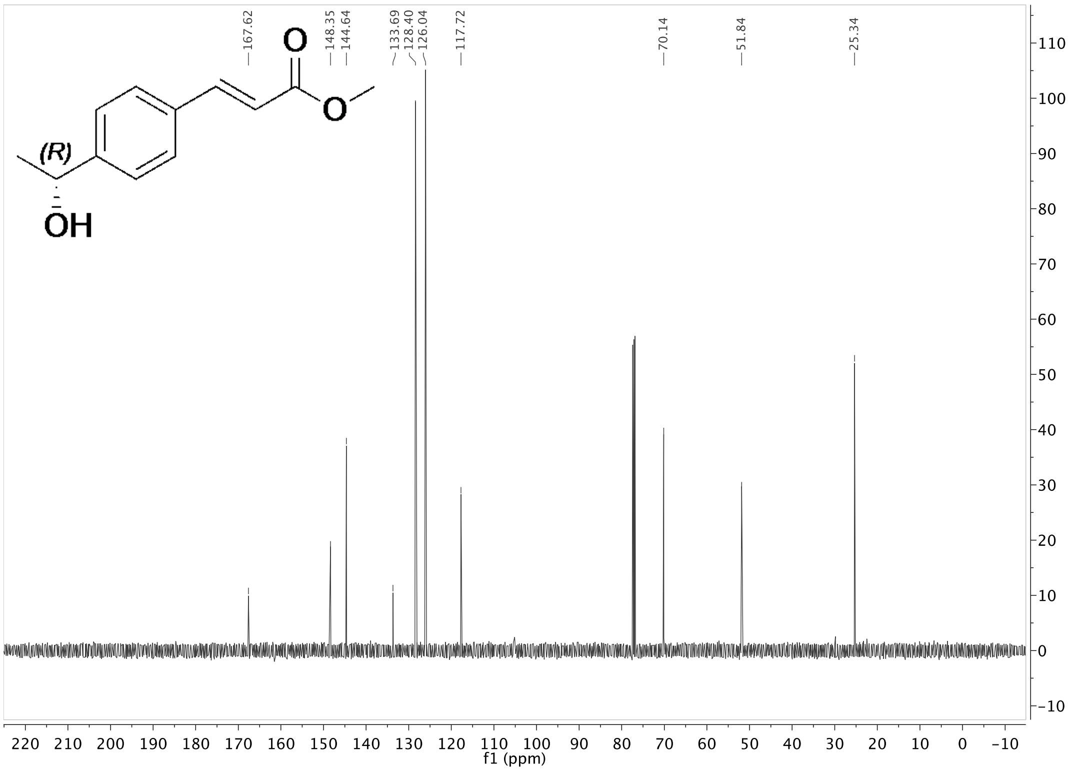

Supplementary figure 32: ^{13}C NMR spectra of 2b

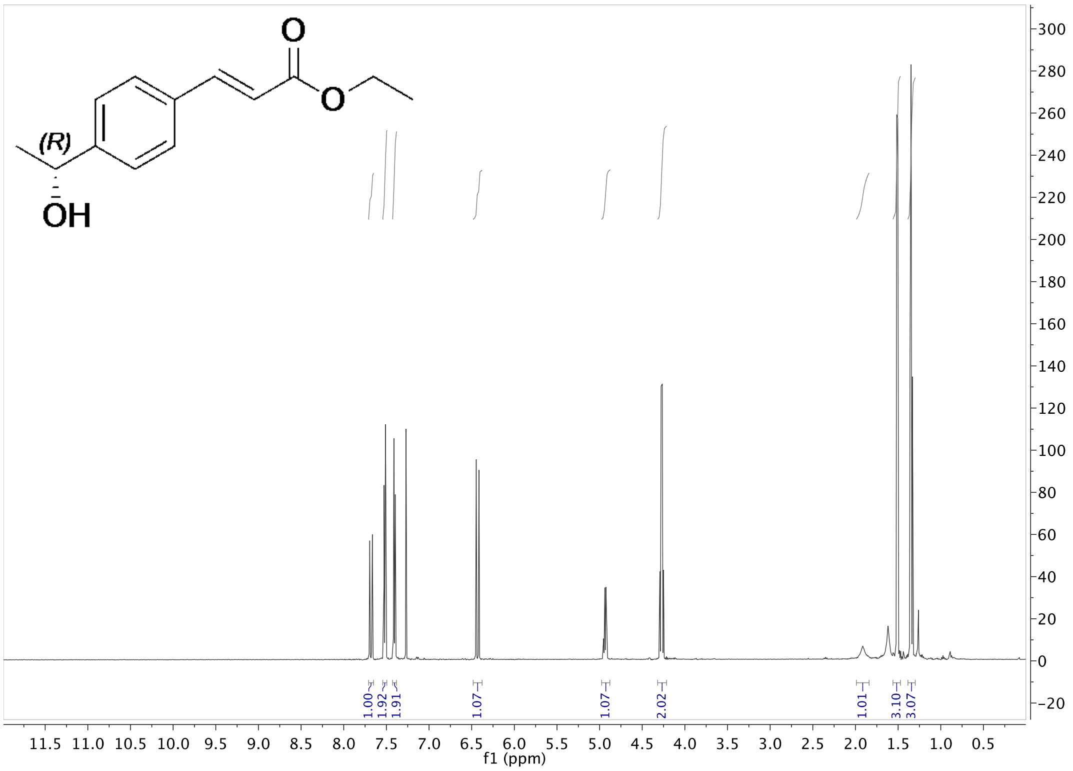

Supplementary figure 33: ^{1}H NMR spectra of 2c

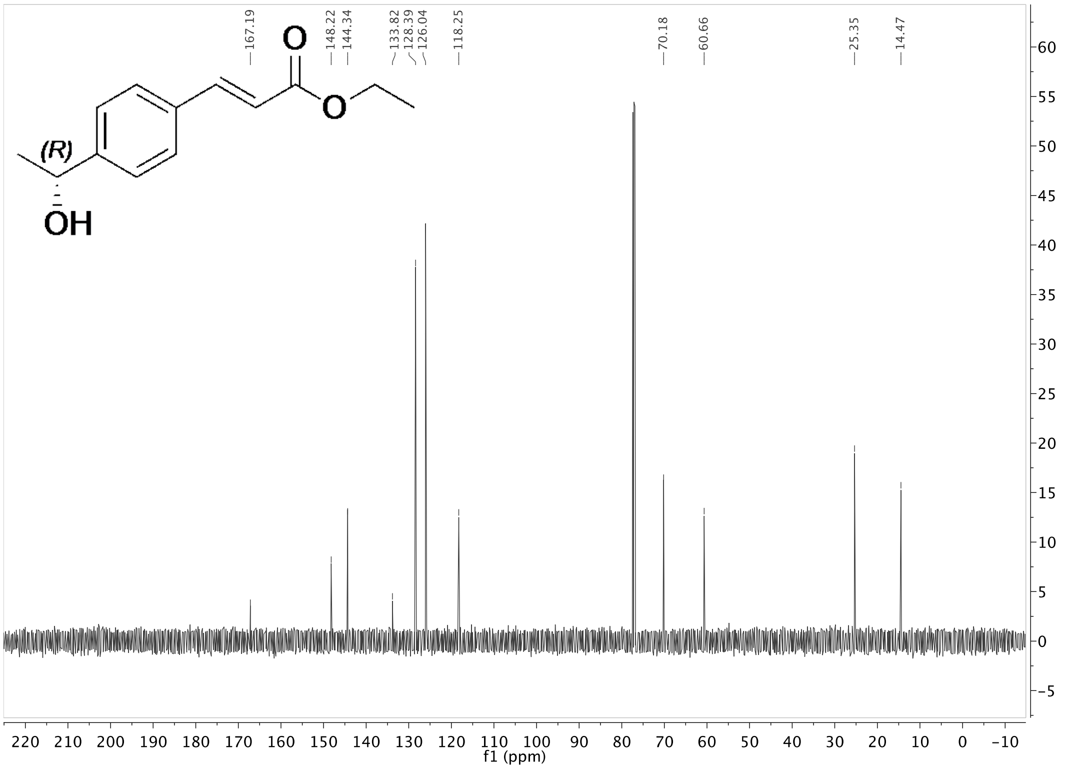

Supplementary figure 34: ^{13}C NMR spectra of 2c

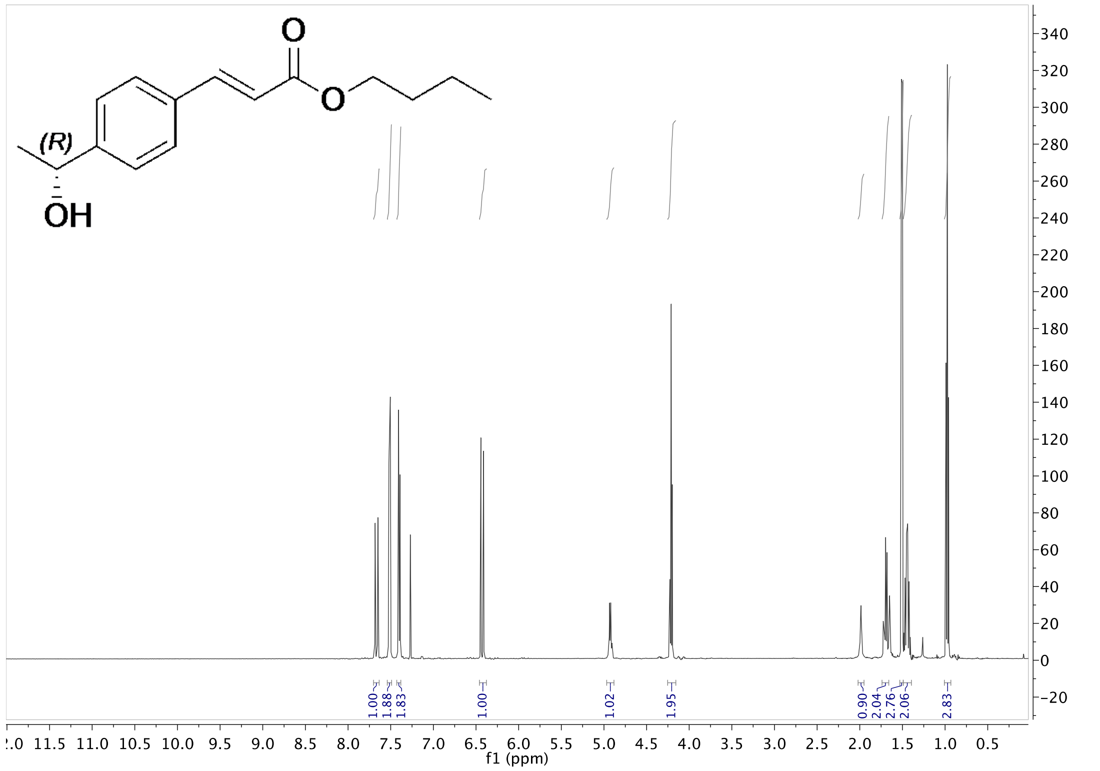

Supplementary figure 35: ^{1}H NMR spectra of 2d

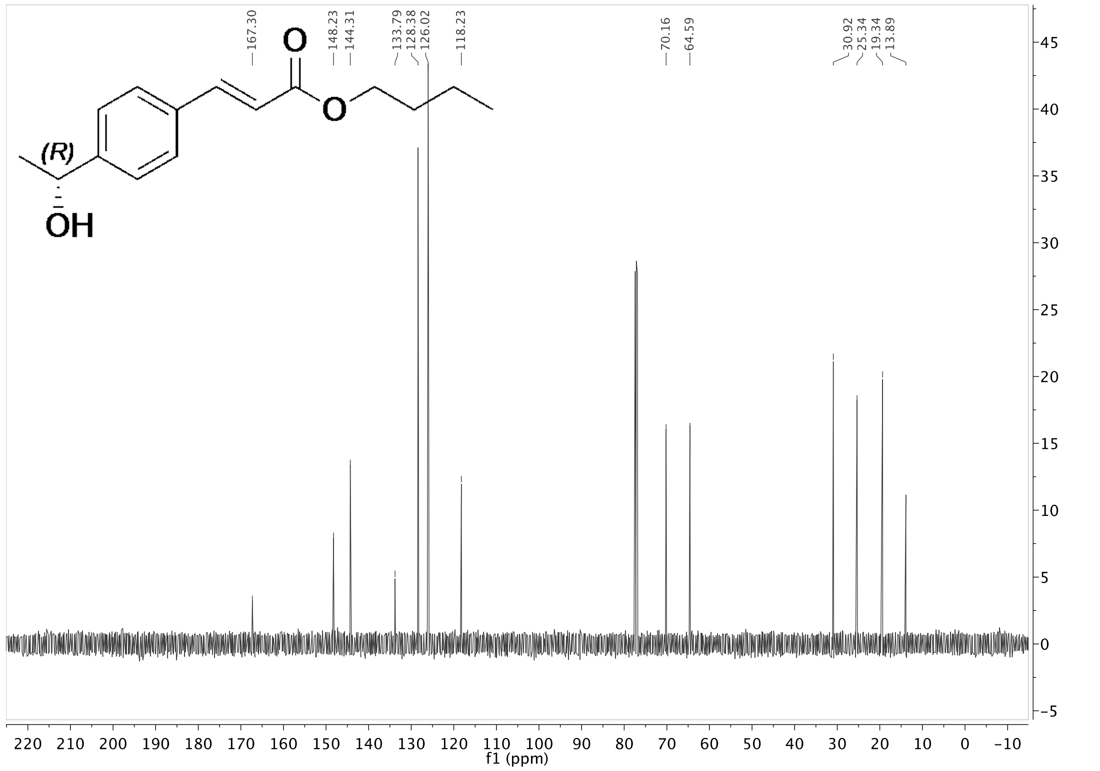

Supplementary figure 36: ^{13}C NMR spectra of 2d

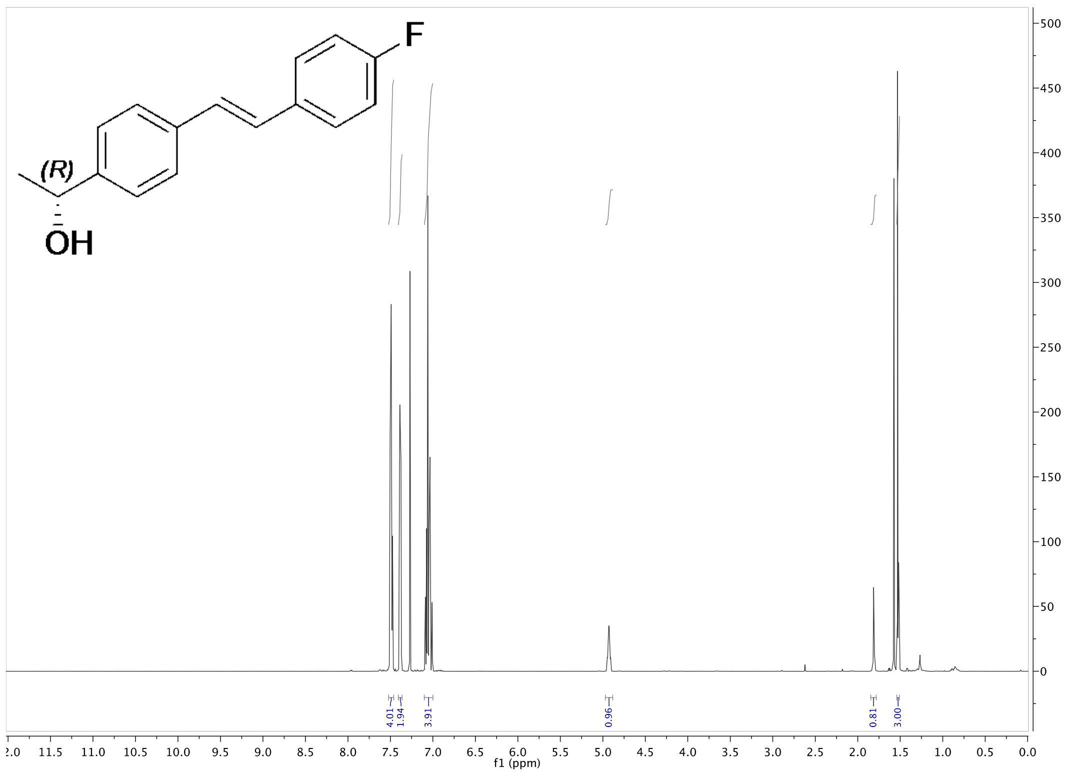

Supplementary figure 37: ^{1}H NMR spectra of 2e

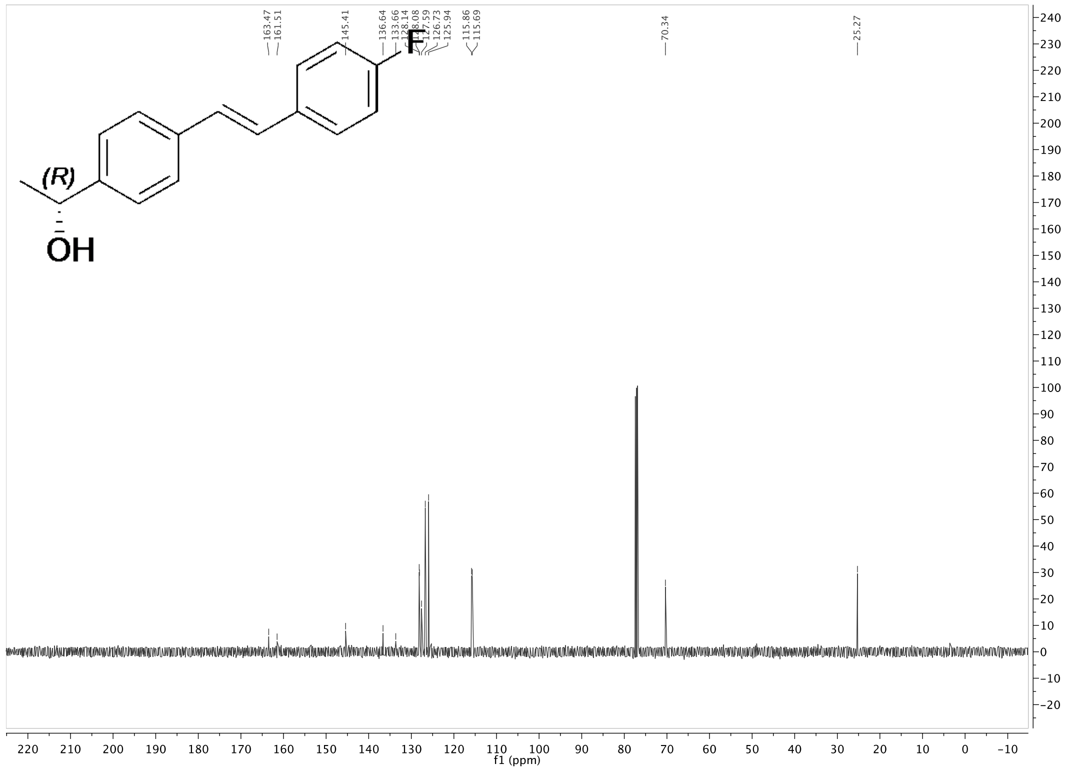

Supplementary figure 38: ^{13}C NMR spectra of 2e

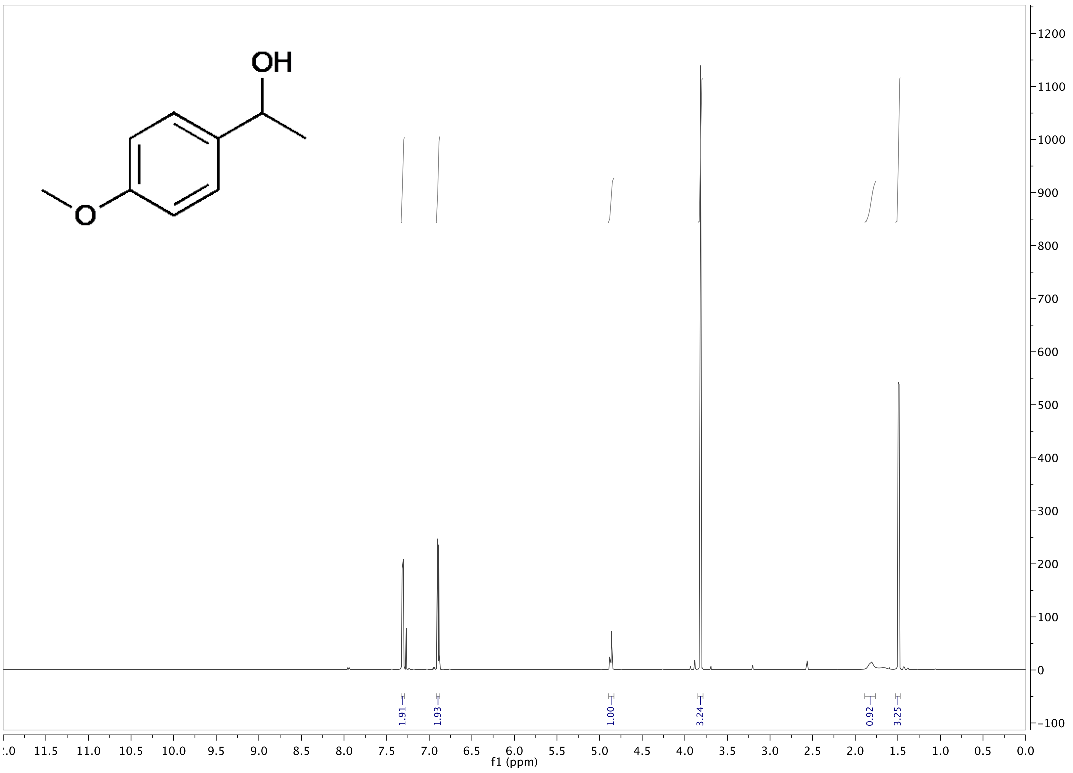

Supplementary figure 39: ^{1}H NMR spectra of 3a

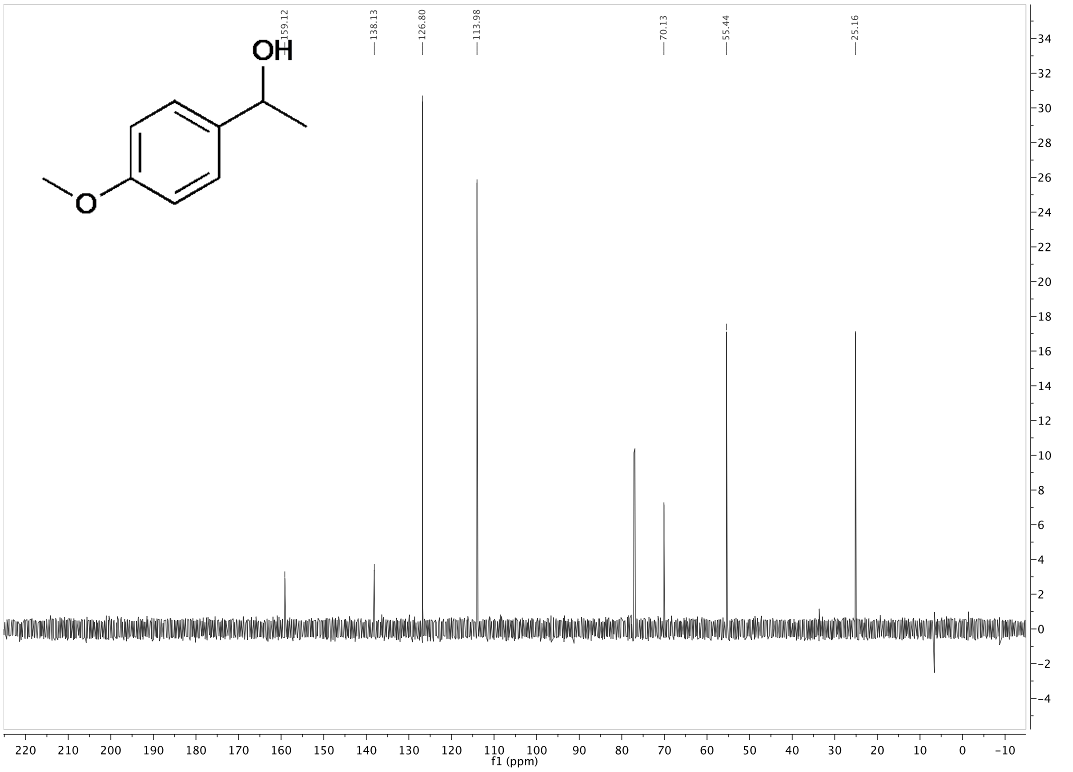

Supplementary figure 40: ^{13}C NMR spectra of 3a

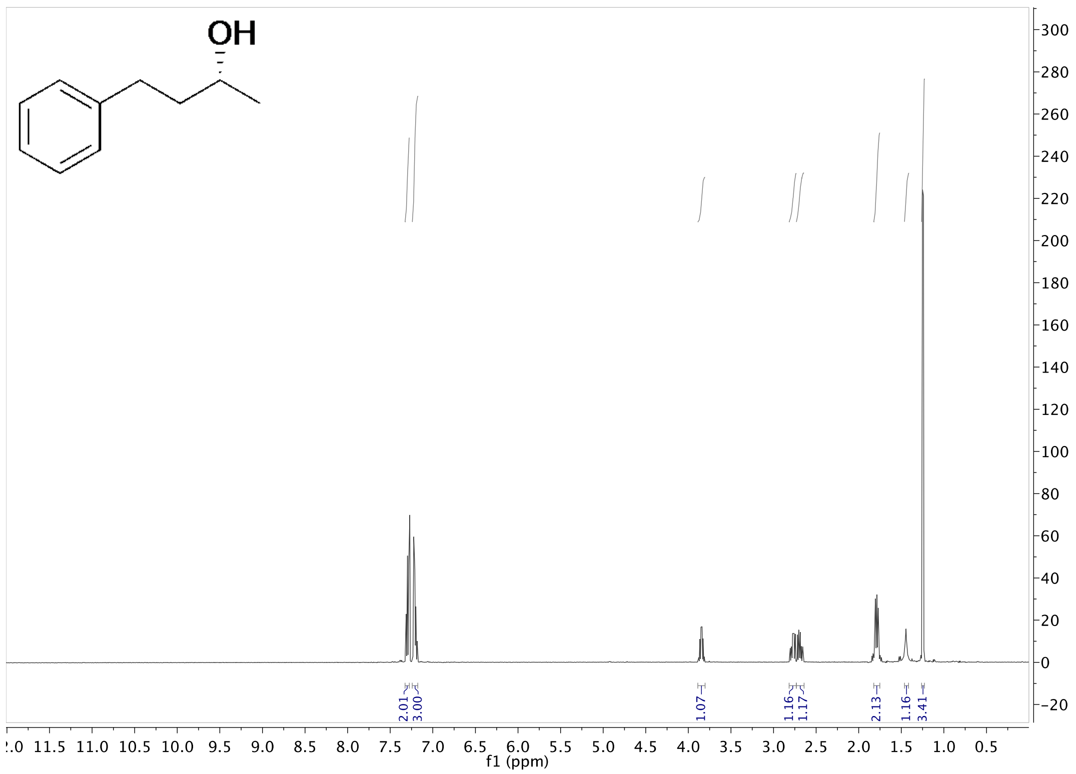

Supplementary figure 41: ^{1}H NMR spectra of 3b/4a

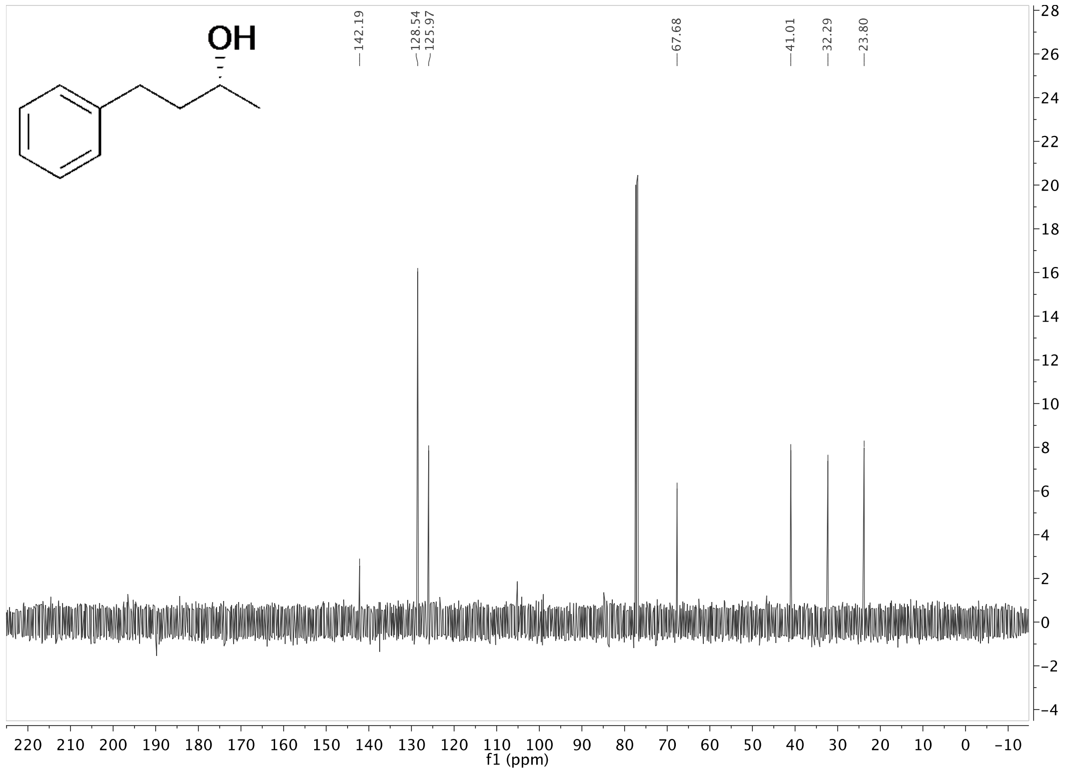

Supplementary figure 42: ^{13}C NMR spectra of 3b/4a

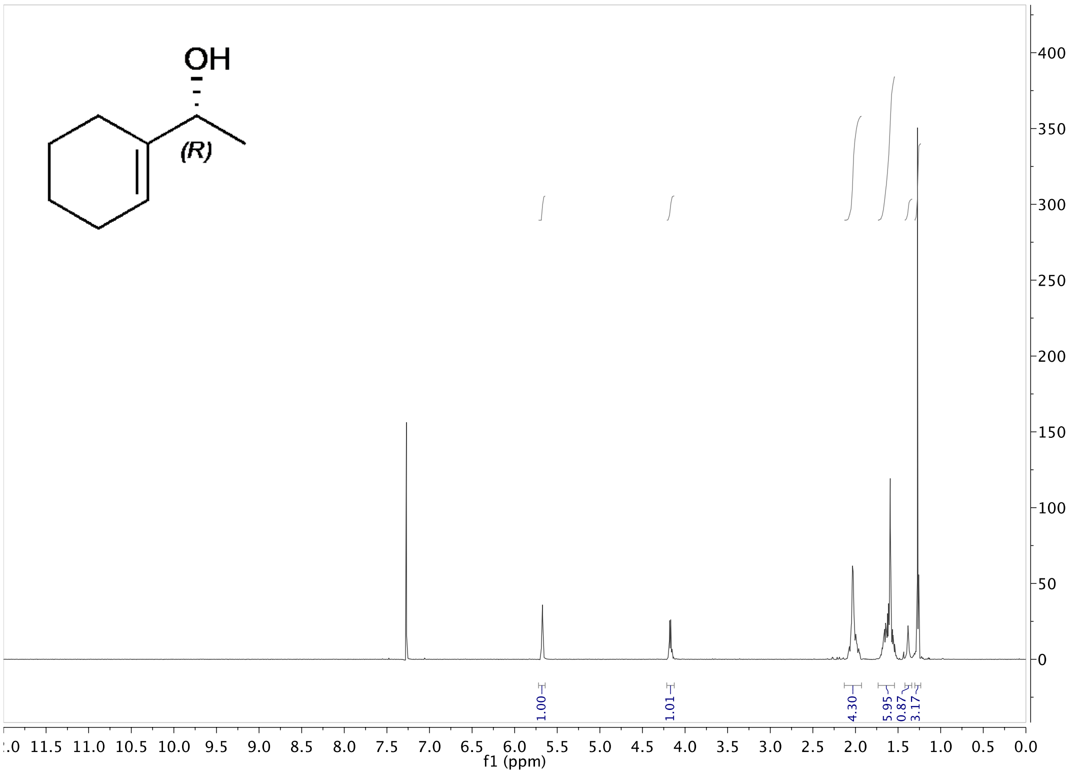

Supplementary figure 43: ^{1}H NMR spectra of 3c

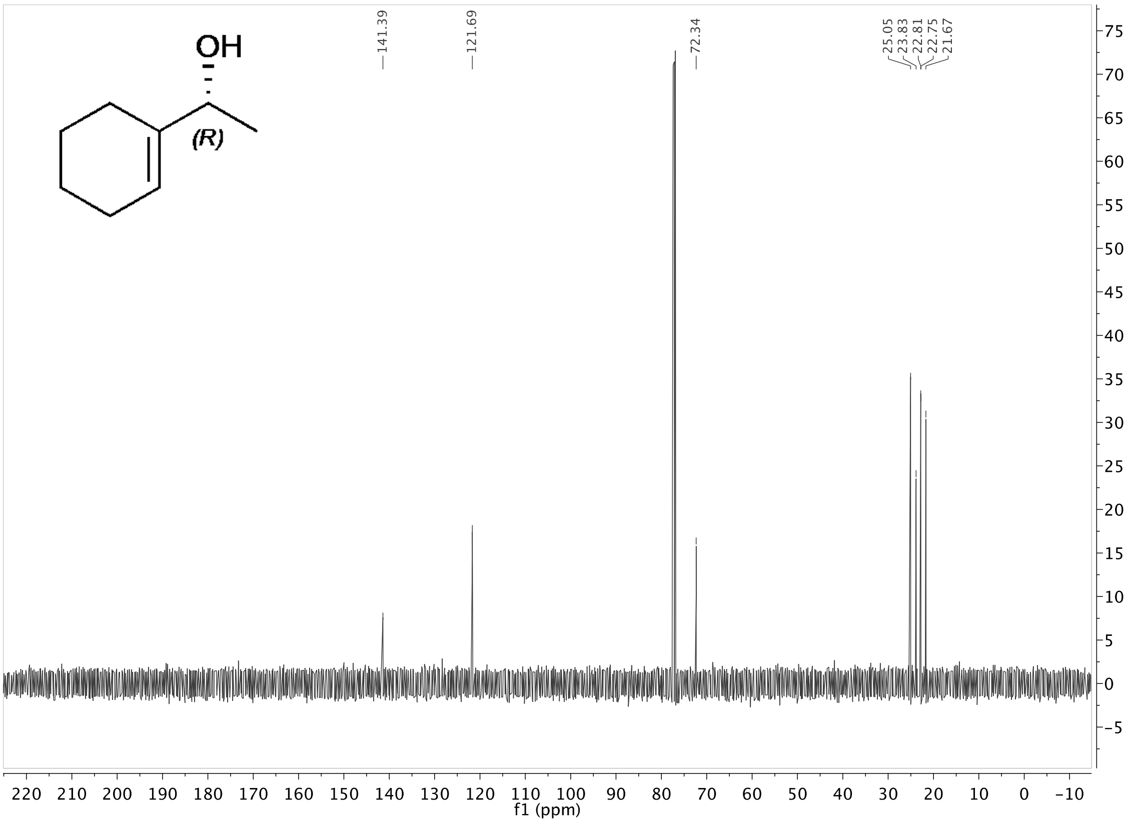

Supplementary figure 44: ^{13}C NMR spectra of 3c

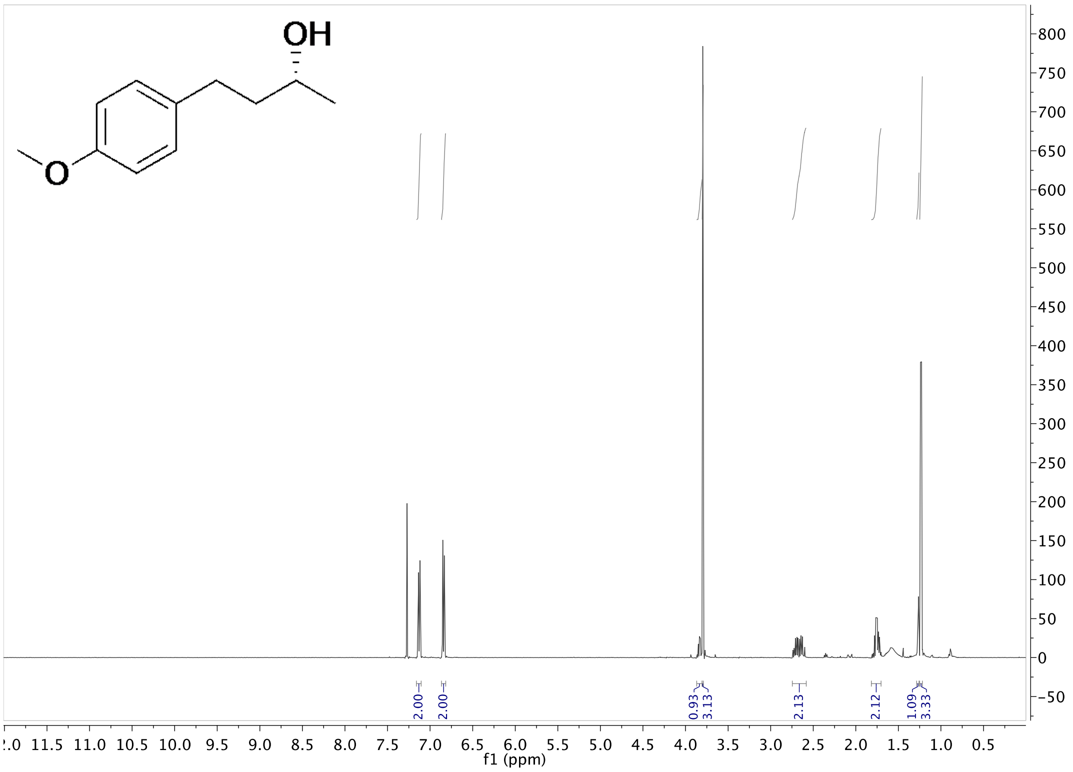

Supplementary figure 45: ^{1}H NMR spectra of 4b

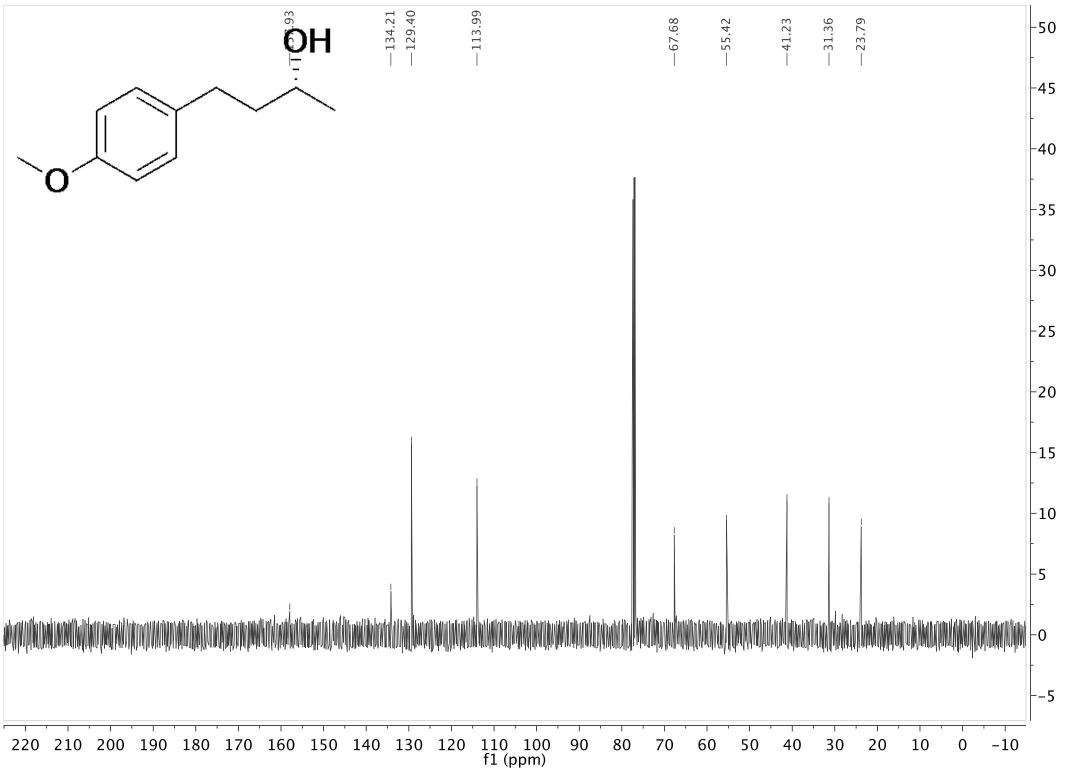

Supplementary figure 46: ^{13}C NMR spectra of 4b

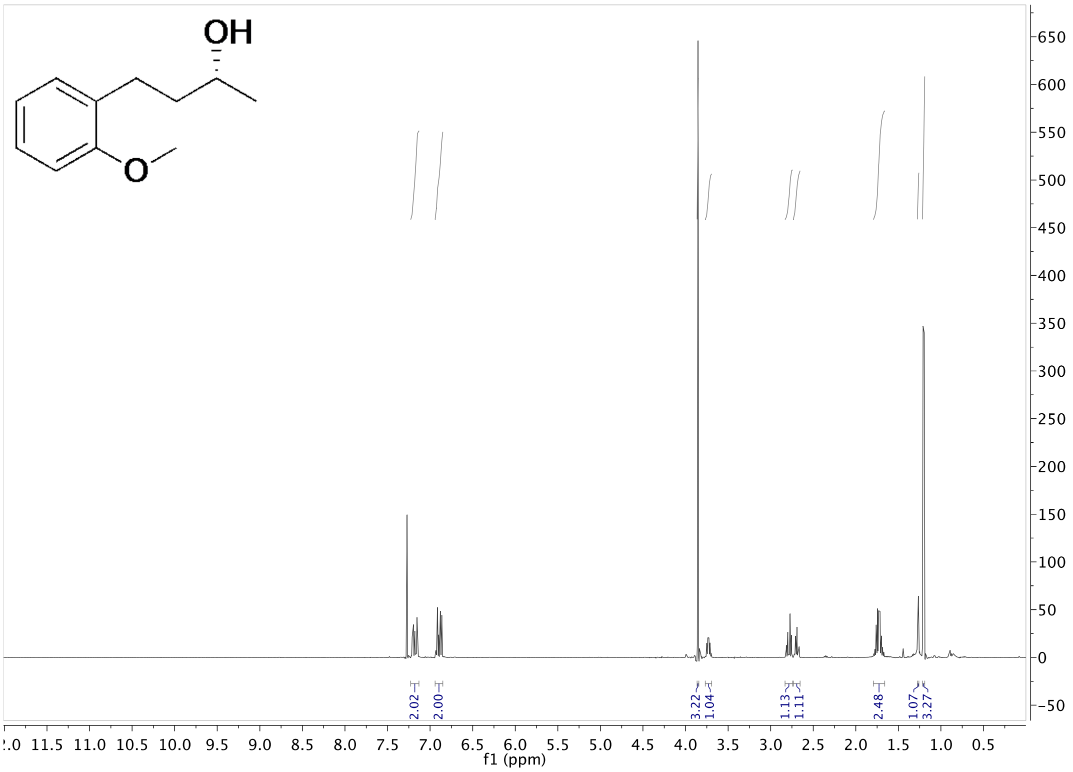

Supplementary figure 47: ^{1}H NMR spectra of 4c

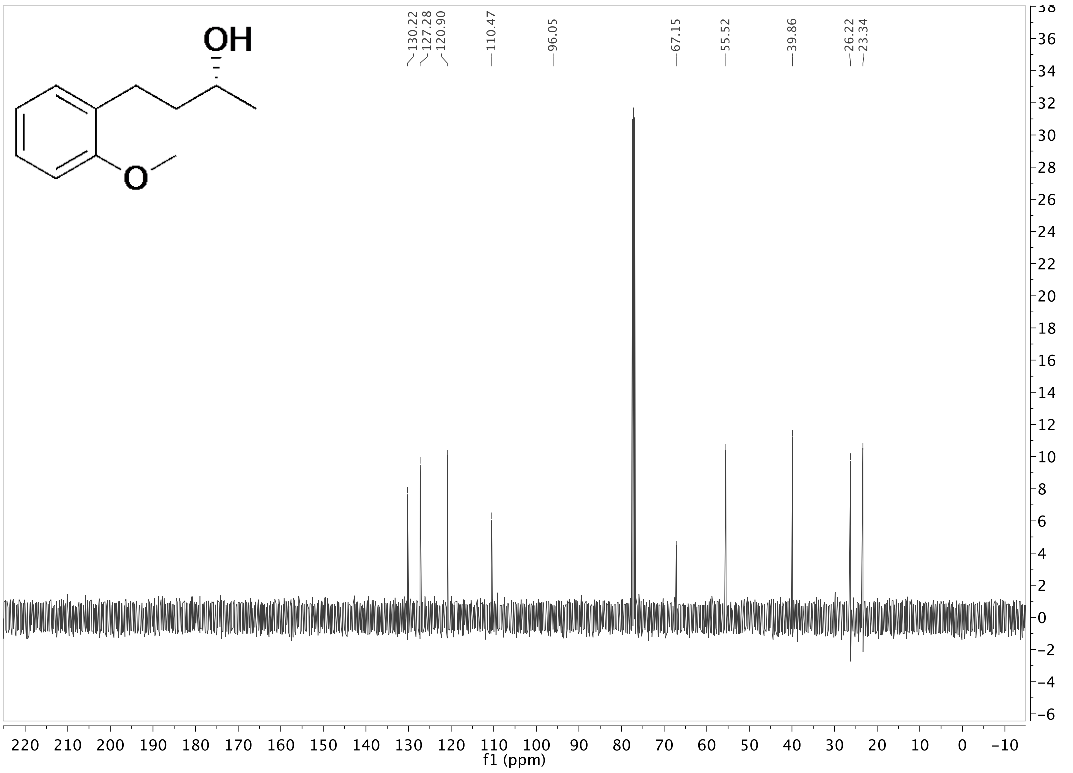

Supplementary figure 48: ^{13}C NMR spectra of 4c

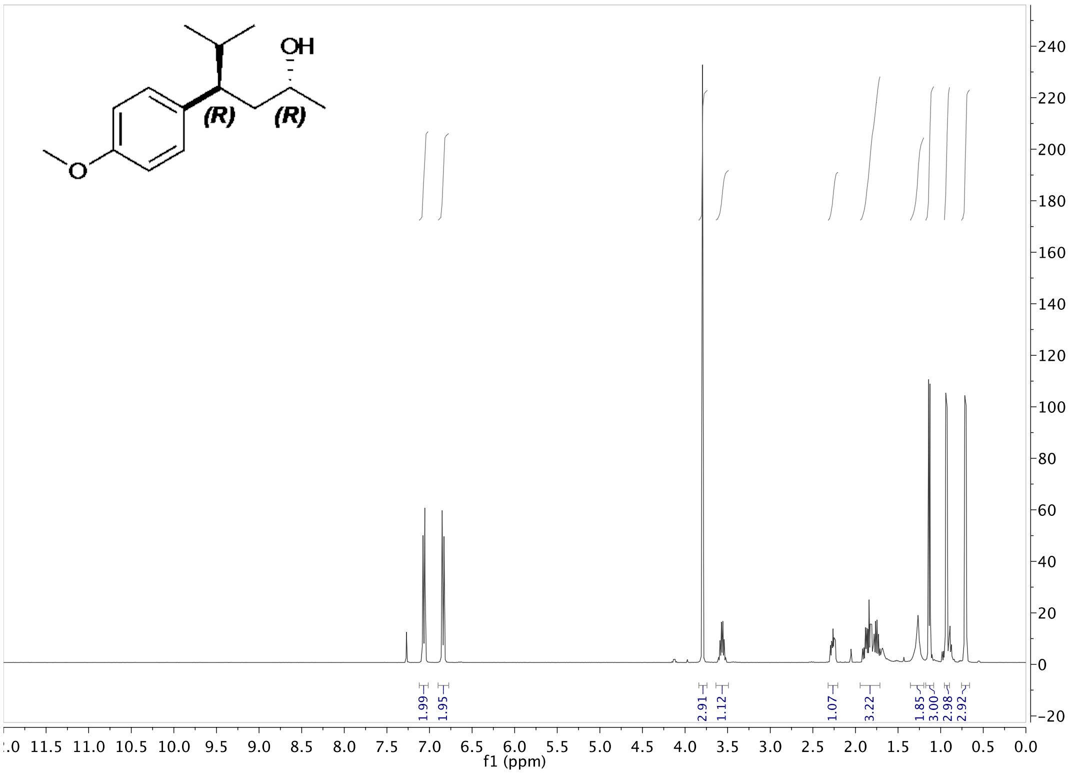

Supplementary figure 49: ^{1}H NMR spectra of 4d

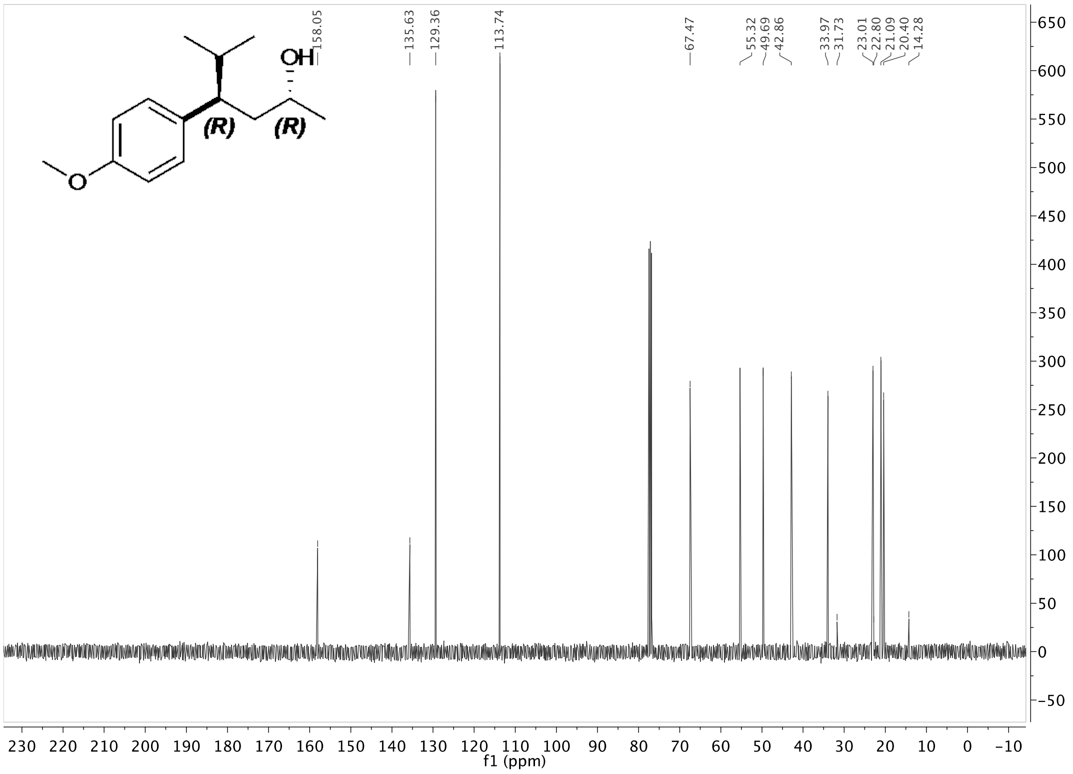

Supplementary figure 50: ^{13}C NMR spectra of 4d

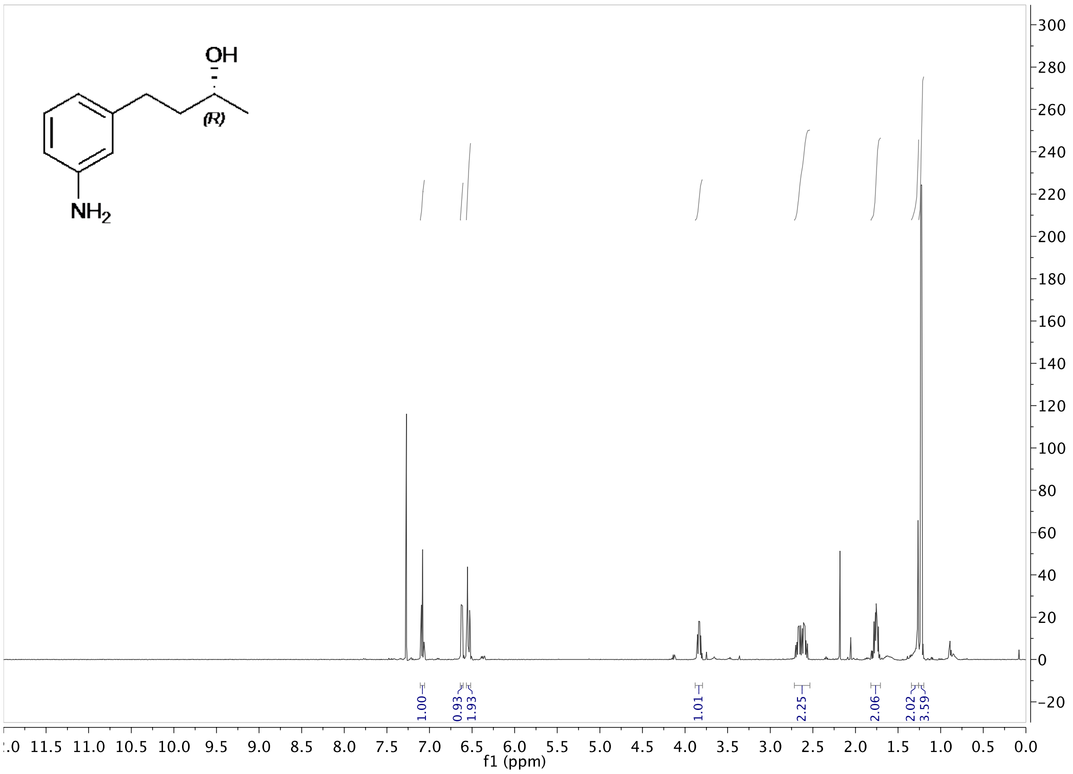

Supplementary figure 51: ^{1}H NMR spectra of 5

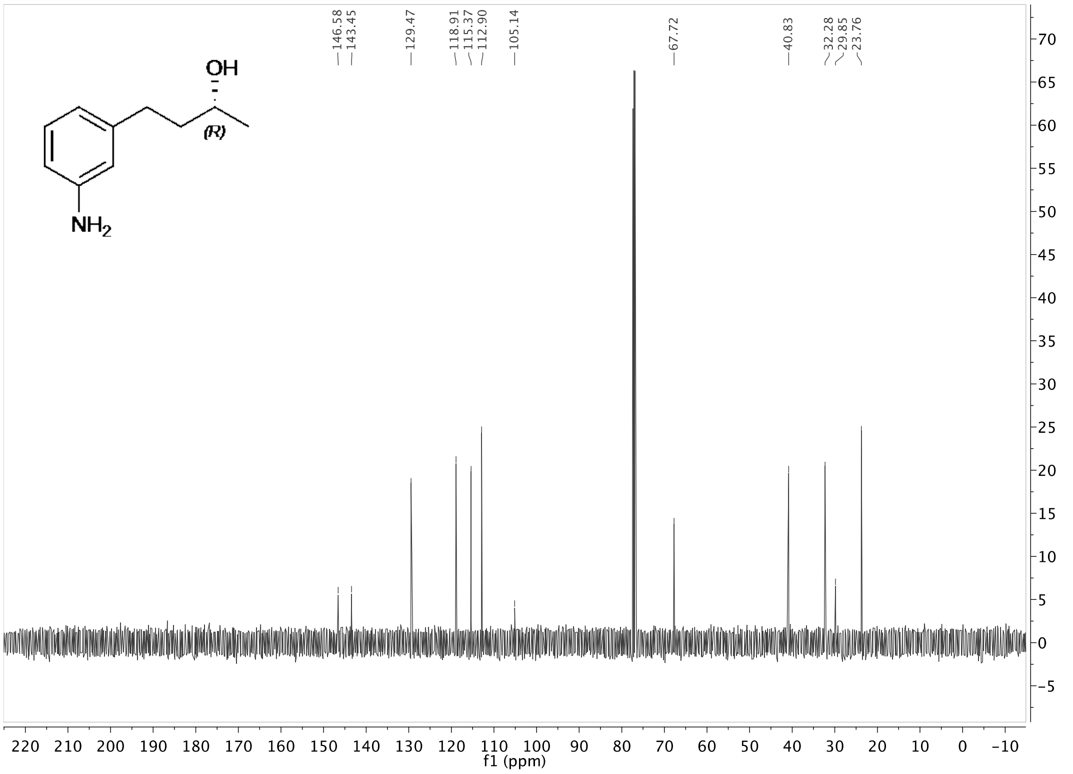

Supplementary figure 52: ^{13}C NMR spectra of 5

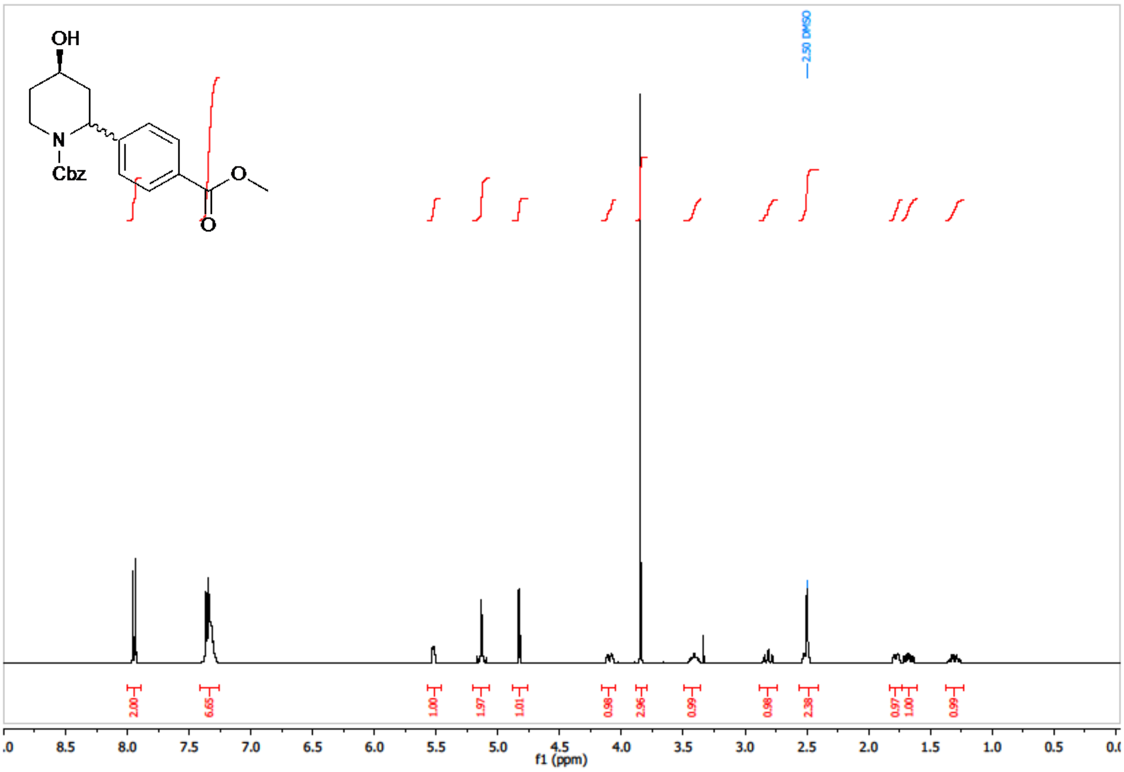

Supplementary figure 53: ^{1}H NMR spectra of 6

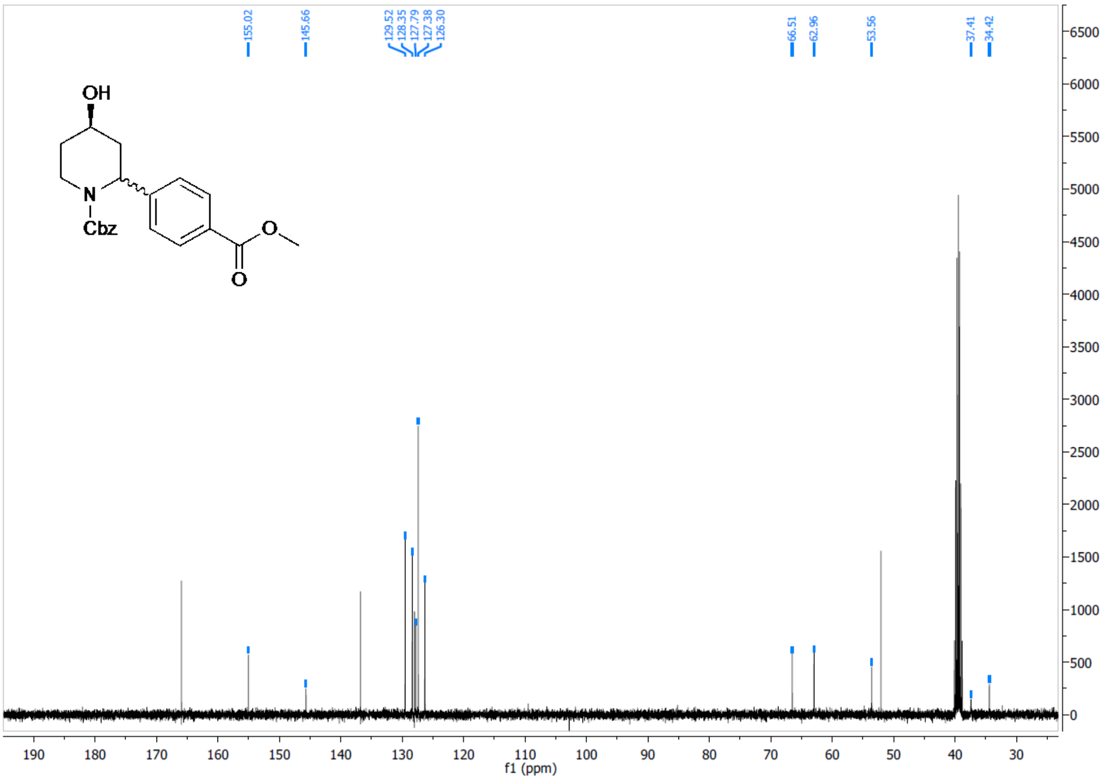

Supplementary figure 54: ^{13}C NMR spectra of 6

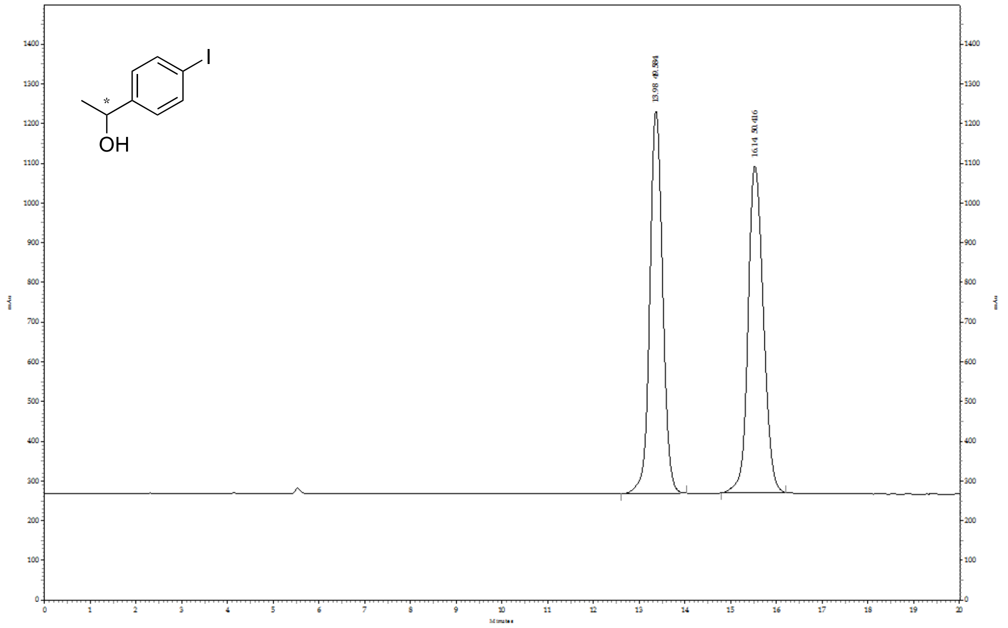

Supplementary figure 55: Chiral HPLC chromatogram of the racemate 1-(4-iodophenyl)ethan-1-ol

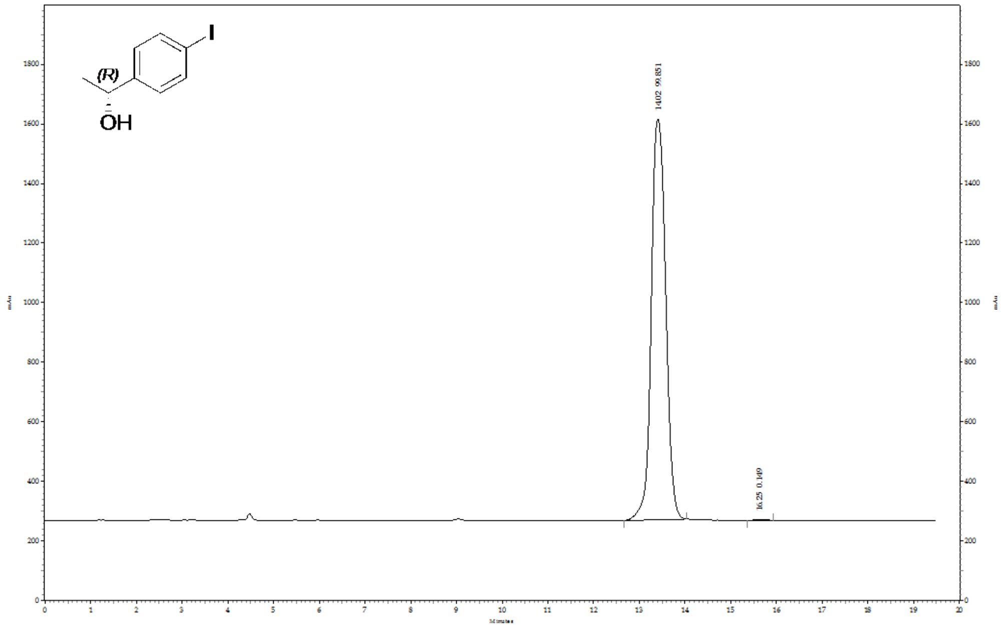

Supplementary figure 56: Chiral HPLC chromatogram of the reaction catalyzed by ADH101 for 1-(4-iodophenyl)ethan-1-ol

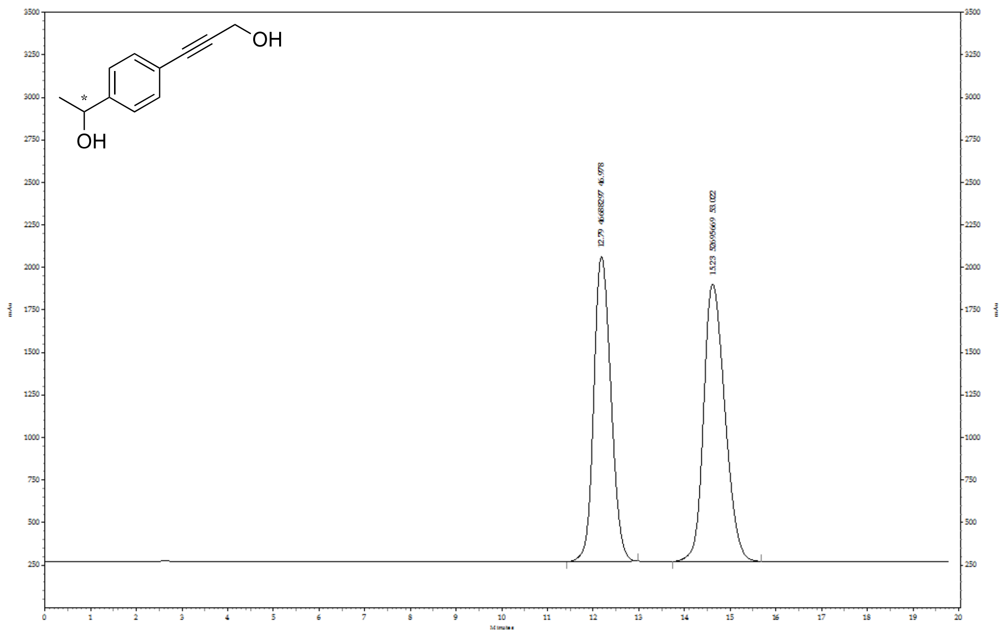

Supplementary figure 57: Chiral HPLC chromatogram of the racemate 1a

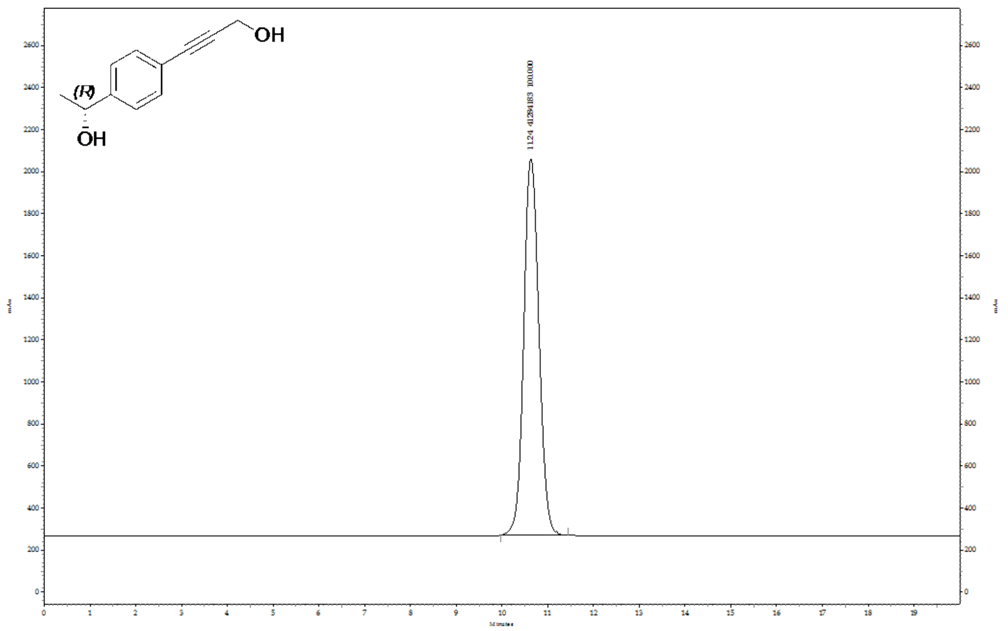

Supplementary figure 58: Chiral HPLC chromatogram of the reaction catalyzed by ADH101 for 1a

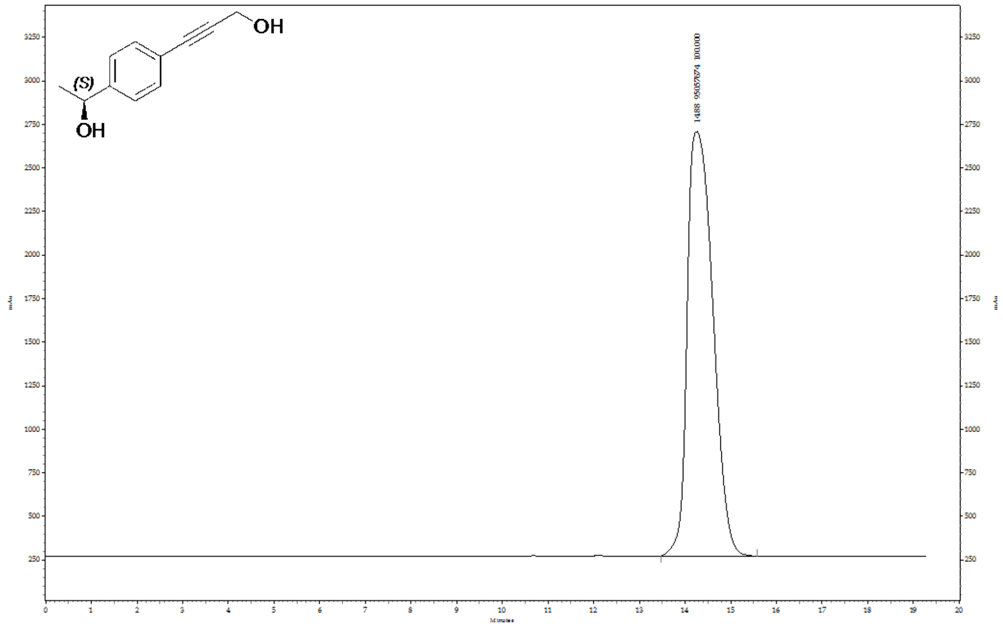

Supplementary figure 59: Chiral HPLC chromatogram of the reaction catalyzed by ADH112 for 1a

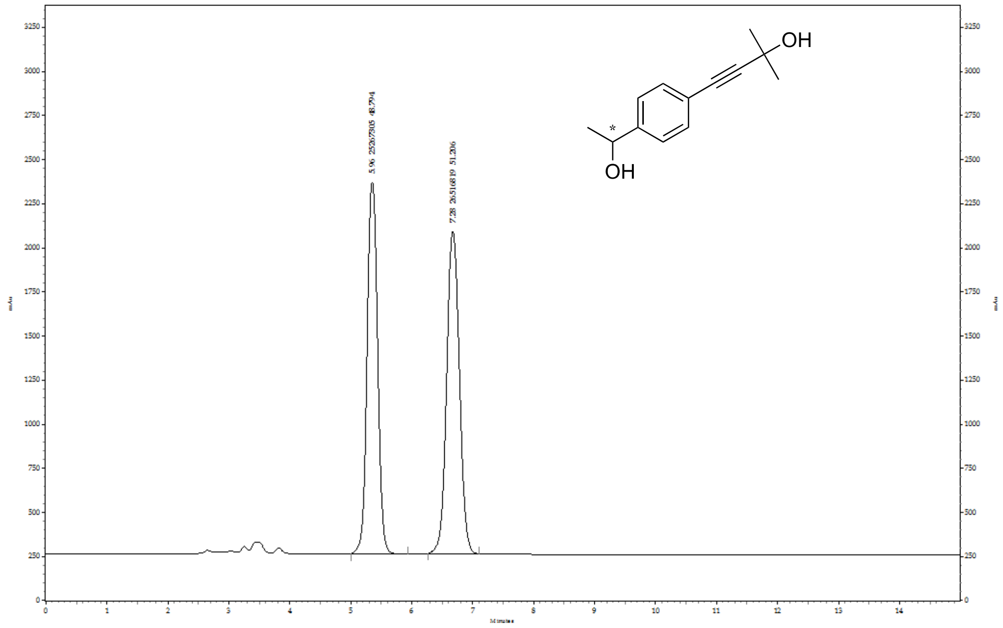

Supplementary figure 60: Chiral HPLC chromatogram of the racemate 1b

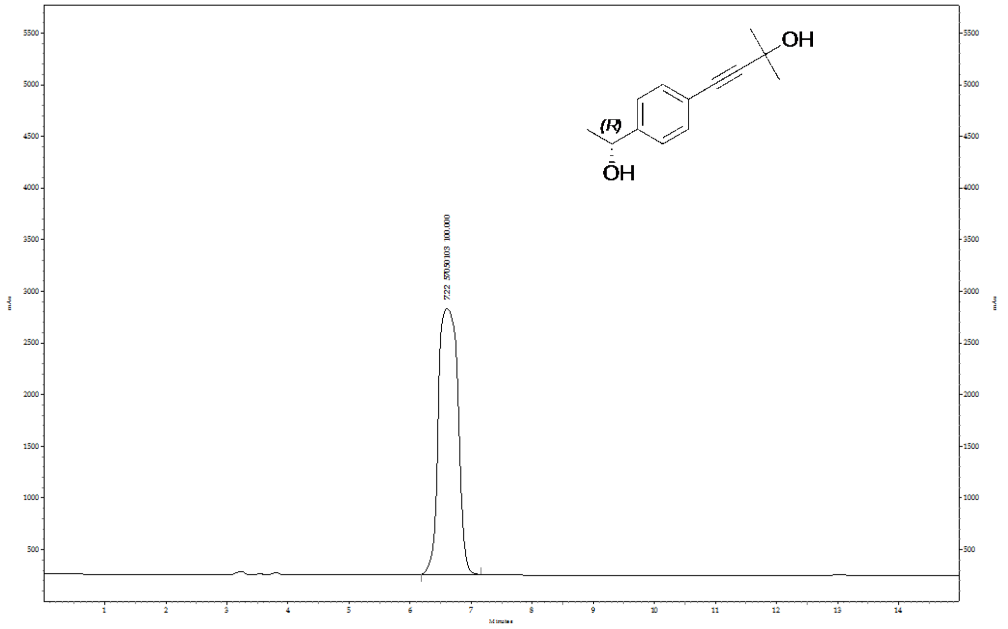

Supplementary figure 61: Chiral HPLC chromatogram of the reaction catalyzed by ADH101 for 1b

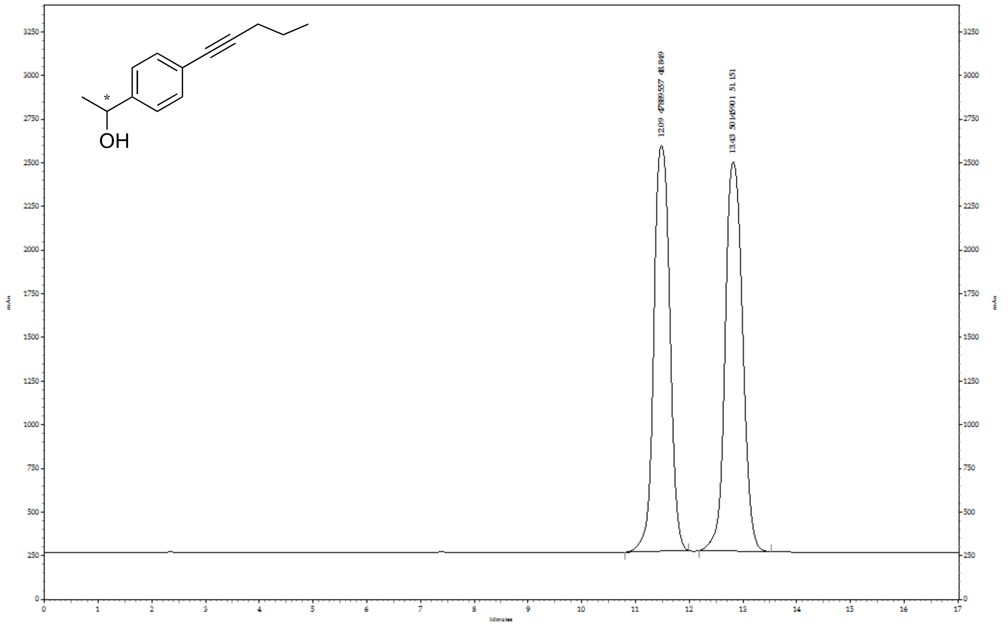

Supplementary figure 62: Chiral HPLC chromatogram of the racemate 1c

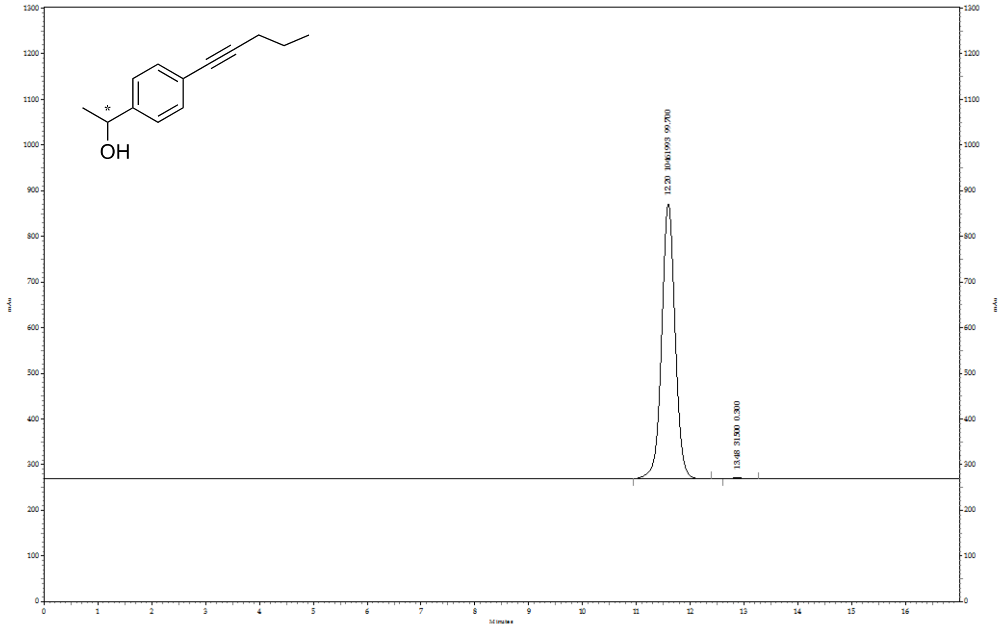

Supplementary figure 63: Chiral HPLC chromatogram of the reaction catalyzed by ADH101 for 1c

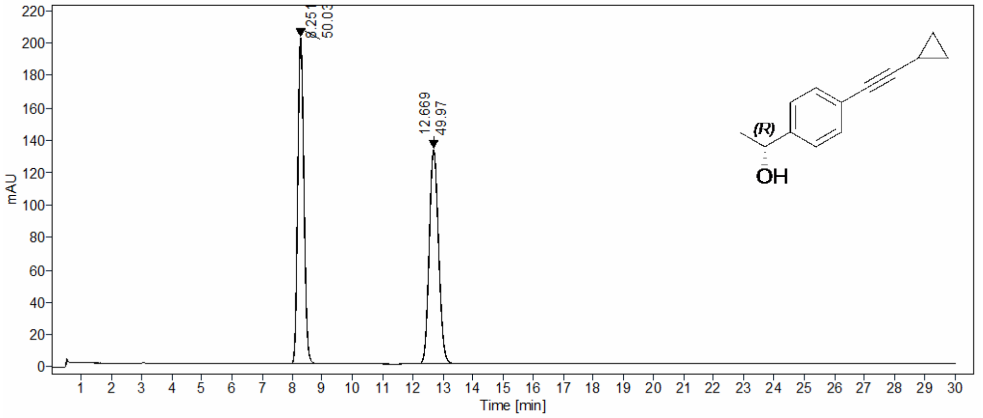

Signal: DAD1A,Sig=245,4 Ref=off

RT [min]	Area	Area%
8.251	2850.2932	50.0332
12.669	2846.5050	49.9668

Supplementary figure 64: Chiral HPLC chromatogram of the racemate 1d

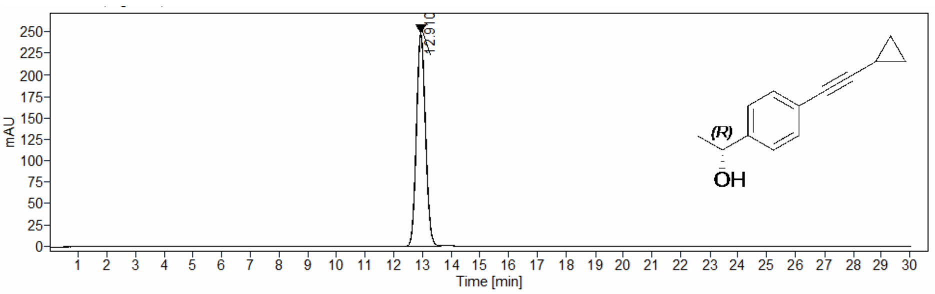

Signal: DAD1A,Sig=245,4 Ref=off

RT [min]	Area	Area%
12.910	5404.8865	100.0000

Supplementary figure 65: Chiral HPLC chromatogram of the reaction catalyzed by ADH101 for 1d

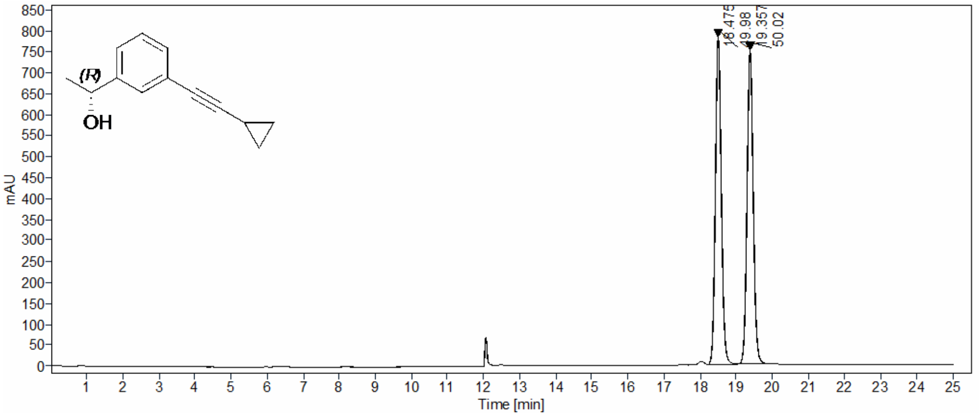

Signal:	DAD1A,Sig=245,4 Ref=off		
	RT [min]	Area	Area%
	18.475	8876.5342	49.9824
	19.357	8882.7724	50.0176

Supplementary figure 66: Chiral HPLC chromatogram of the racemate 1e

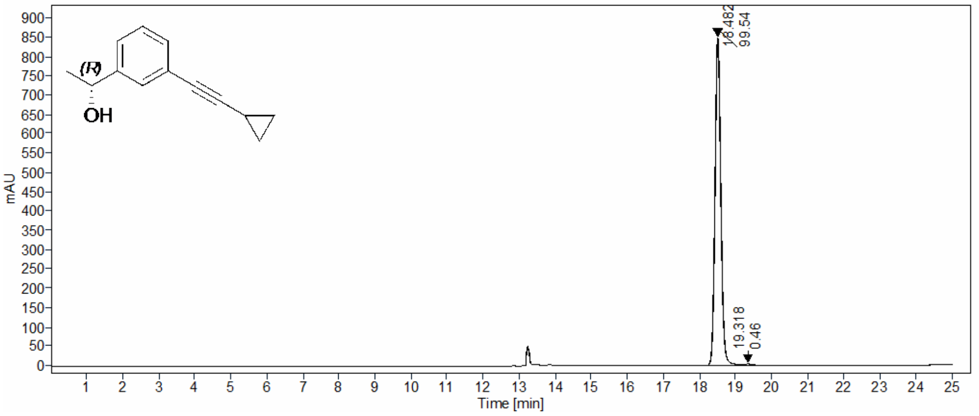

Signal:	DAD1A,Sig=245,4 Ref=off		
	RT [min]	Area	Area%
	18.482	9518.1775	99.5355
	19.318	44.4151	0.4645

Supplementary figure 67: Chiral HPLC chromatogram of the reaction catalyzed by ADH101 for 1e

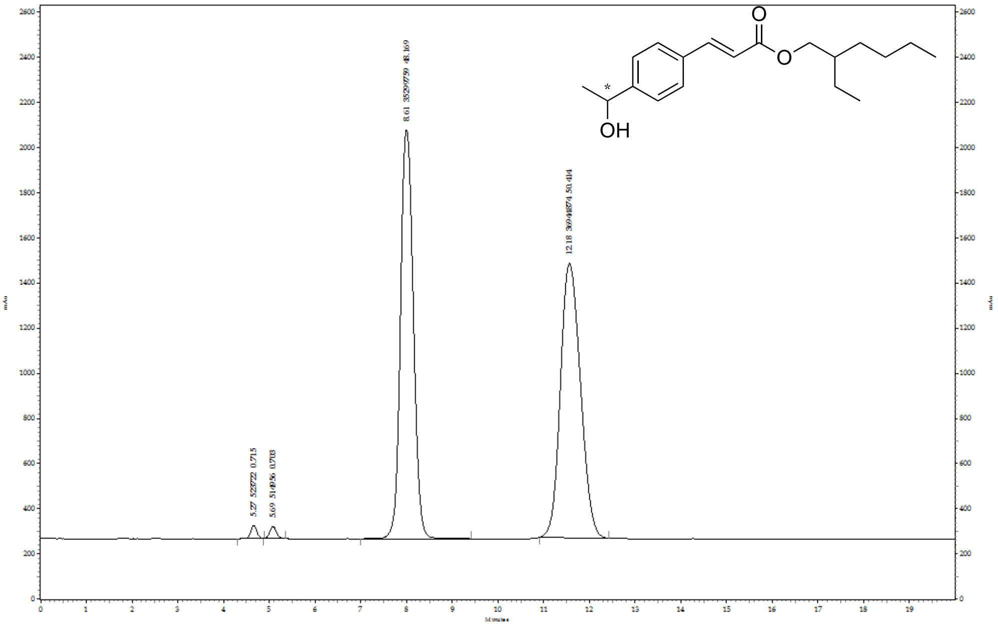

Supplementary figure 68: Chiral HPLC chromatogram of the racemate of 2a

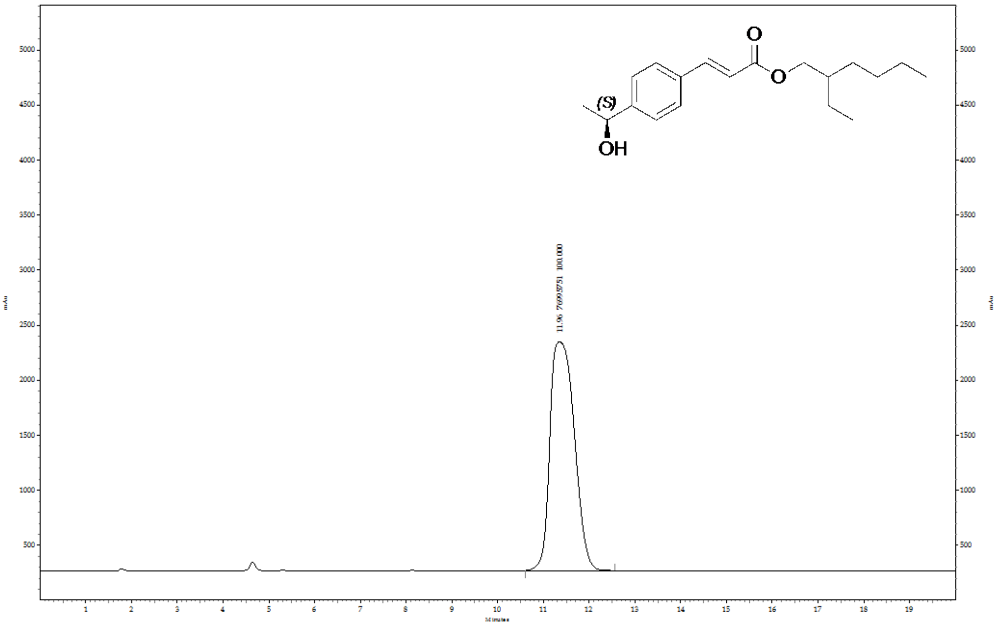

Supplementary figure 69: Chiral HPLC chromatogram of the reaction catalyzed by ADH112 for 2a

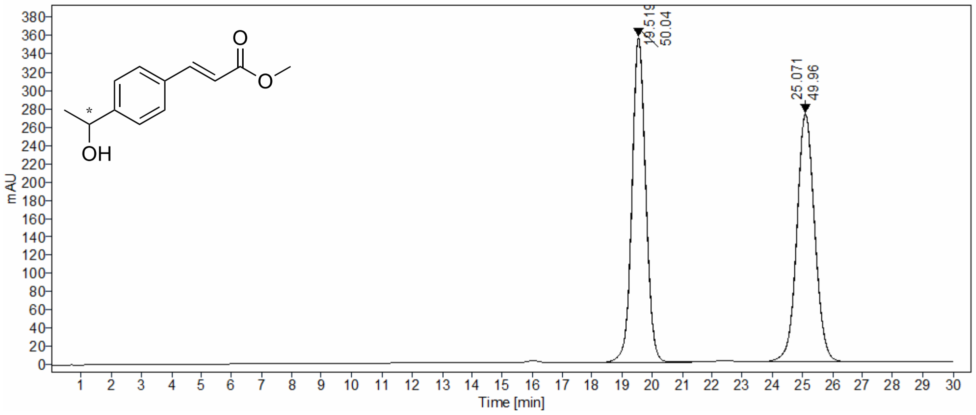

Signal: DAD1A,Sig=275,4 Ref=off

RT [min]	Area	Area%
19.519	11271.3008	50.0371
25.071	11254.5693	49.9629

Supplementary figure 70: Chiral HPLC chromatogram of the racemate of 2b

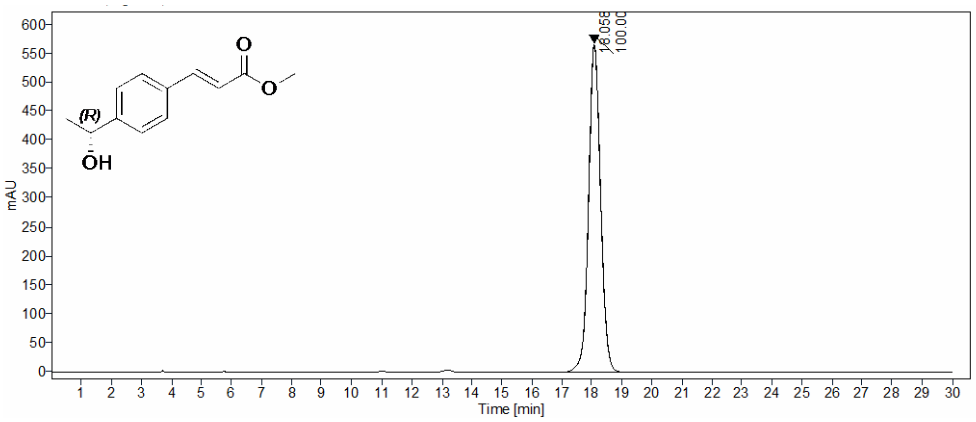

Signal: DAD1A,Sig=275,4 Ref=off

RT [min]	Area	Area%
18.058	15722.4491	100.0000

Supplementary figure 71: Chiral HPLC chromatogram of the reaction catalyzed by ADH101 for 2b

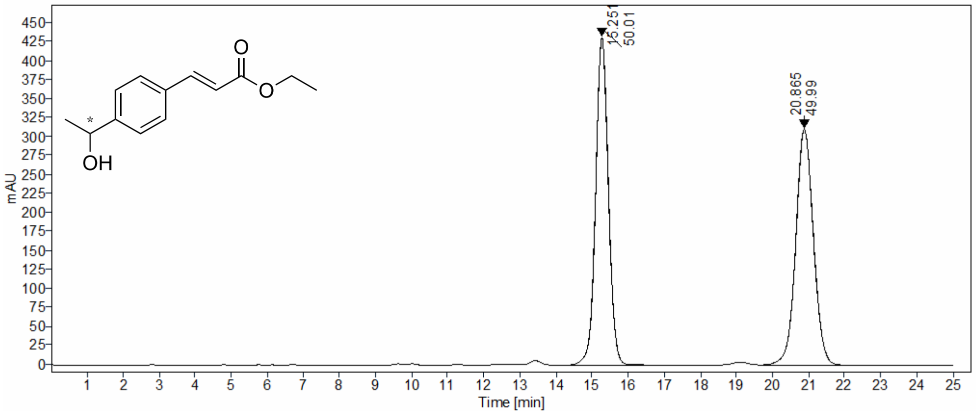

Signal:	DAD1A,Sig=275,4 Ref=off		
	RT [min]	Area	Area%
	15.251	10614.7483	50.0125
	20.865	10609.4553	49.9875

Supplementary figure 72: Chiral HPLC chromatogram of the racemate of 2c

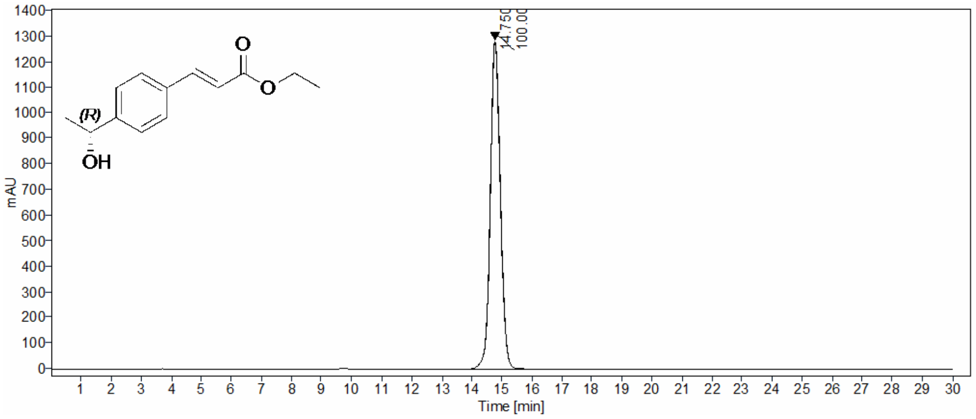

Signal:	DAD1A,Sig=275,4 Ref=off		
	RT [min]	Area	Area%
	14.750	29878.4082	100.0000

Supplementary figure 73: Chiral HPLC chromatogram of the reaction catalyzed by ADH101 for 2c

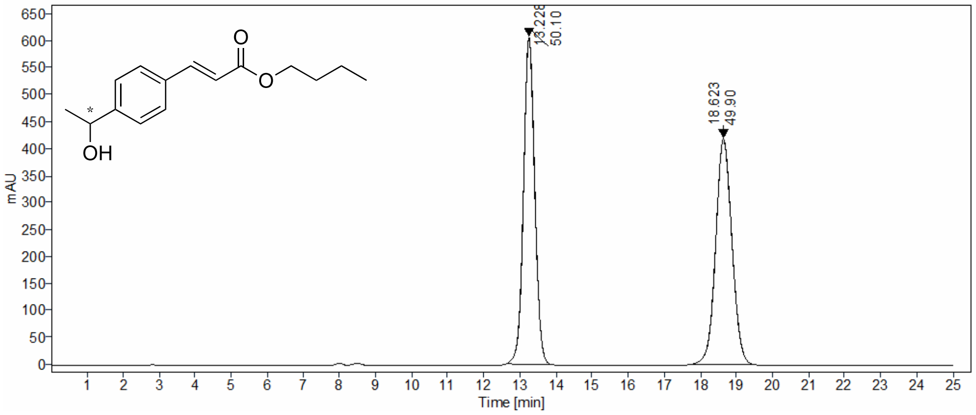

Signal:	DAD1A,Sig=275,4 Ref=off		
	RT [min]	Area	Area%
	13.228	13106.5645	50.0983
	18.623	13055.1367	49.9017

Supplementary figure 74: Chiral HPLC chromatogram of the racemate of 2d

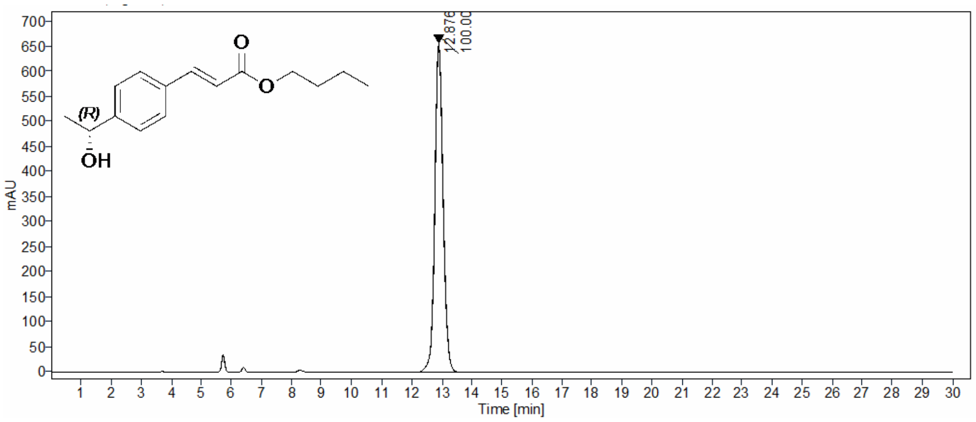

Signal:	DAD1A,Sig=275,4 Ref=off		
	RT [min]	Area	Area%
	12.876	12548.9631	100.0000

Supplementary figure 75: Chiral HPLC chromatogram of the reaction catalyzed by ADH101 for 2d

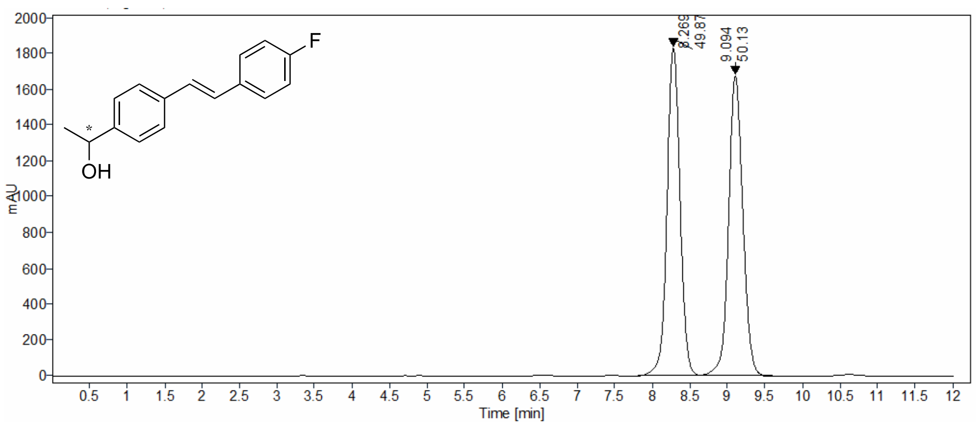

Signal:	DAD1A,Sig=275,4 Ref=off		
	RT [min]	Area	Area%
	8.269	21952.6575	49.8702
	9.094	22066.8901	50.1298

Supplementary figure 76: Chiral HPLC chromatogram of the racemate of 2e

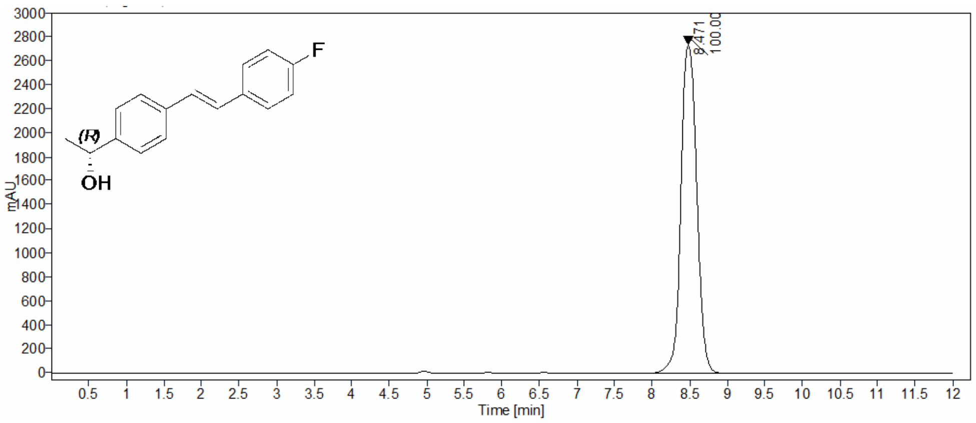

Signal:	DAD1A,Sig=275,4 Ref=off		
	RT [min]	Area	Area%
	8.471	40015.7533	100.0000

Supplementary figure 77: Chiral HPLC chromatogram of the reaction catalyzed by ADH101 for 2e

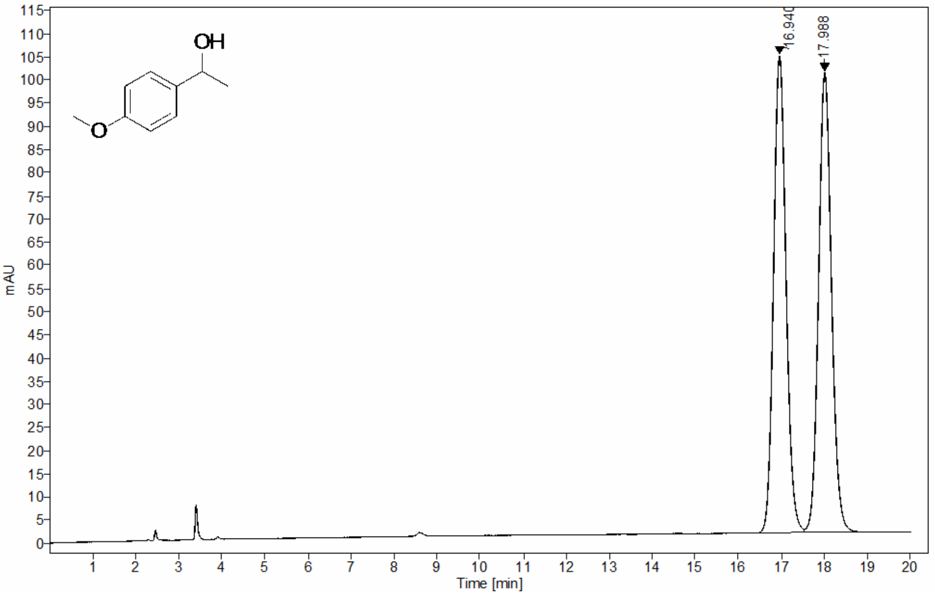

Signal: DAD1A,Sig=230,4 Ref=off

RT [min]	Area	Area%
16.940	2062.6187	49.9028
17.988	2070.6502	50.0972

Supplementary figure 78: Chiral HPLC chromatogram of the racemate 3a

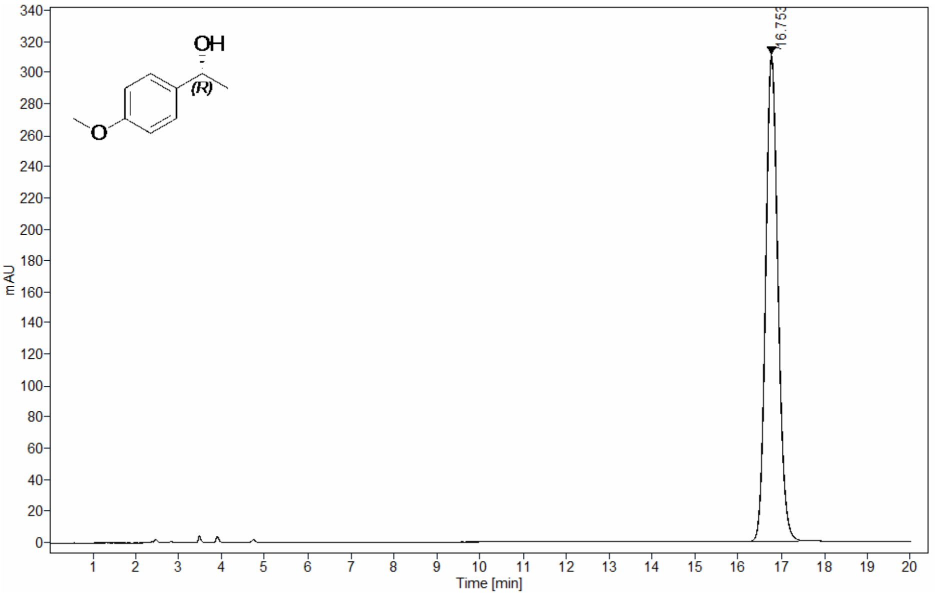

Signal: DAD1A,Sig=230,4 Ref=off

RT [min]	Area	Area%
16.753	6168.0357	100.0000

Supplementary figure 79: Chiral HPLC chromatogram of the reaction catalyzed by ADH101 for 3a

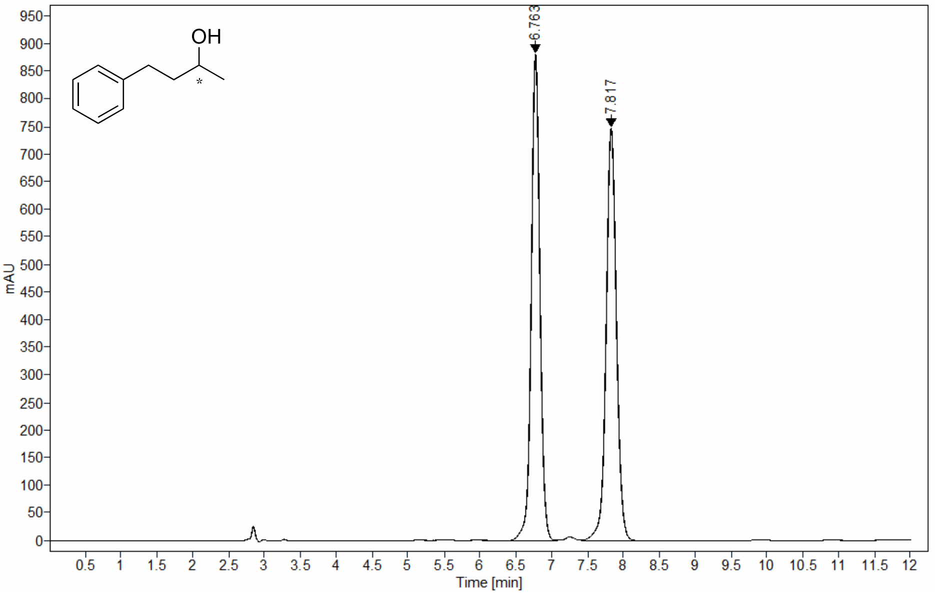

Signal: DAD1A,Sig=210,4 Ref=off

RT [min]	Area	Area%
6.763	7394.2139	49.7560
7.817	7466.7338	50.2440

Supplementary figure 80: Chiral HPLC chromatogram of the racemate 3b

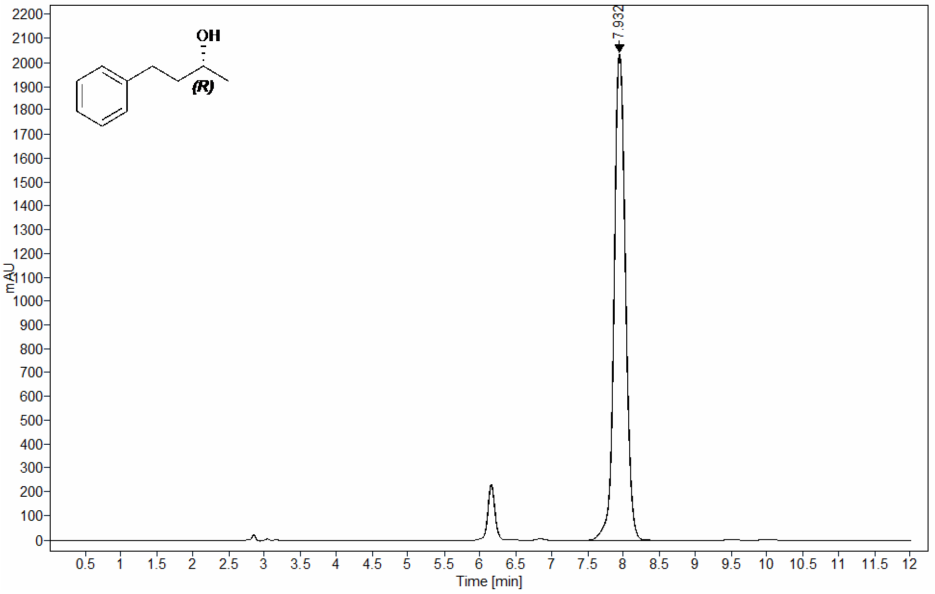

Signal: DAD1A,Sig=210,4 Ref=off

RT [min]	Area	Area%
7.932	22472.4771	100.0000

Supplementary figure 81: Chiral HPLC chromatogram of the reaction catalyzed by ADH101 for 3b

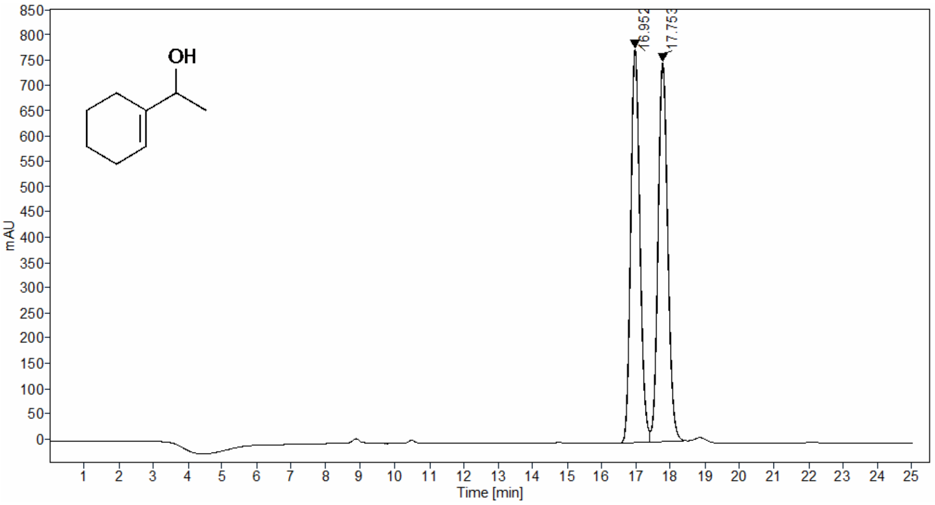

Signal:	DAD1A, Sig=210,4 Ref=off		
	RT [min]	Area	Area%
	16.952	14396.9785	49.5798
	17.753	14640.9917	50.4202

Supplementary figure 82: Chiral HPLC chromatogram of the racemate 3c

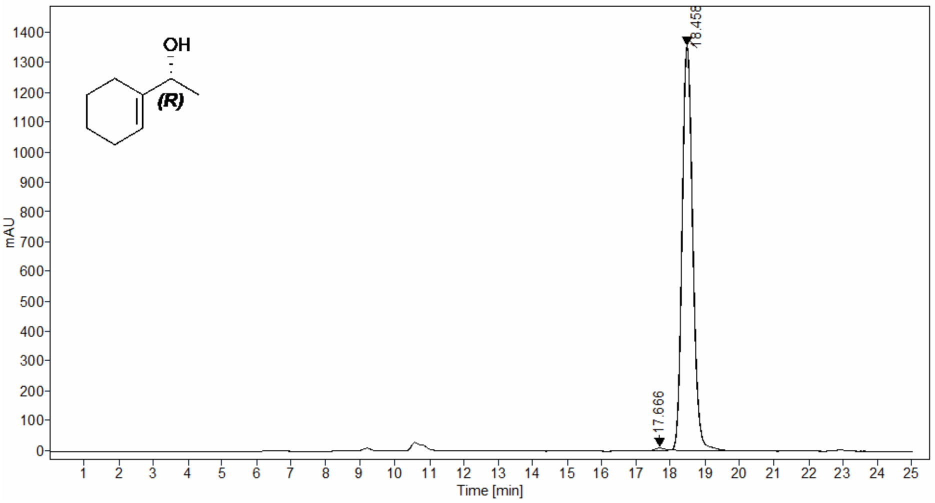

Signal:	DAD1A, Sig=210,4 Ref=off		
	RT [min]	Area	Area%
	17.666	143.8802	0.4935
	18.458	29013.3826	99.5065

Supplementary figure 83: Chiral HPLC chromatogram of the reaction catalyzed by ADH101 for 3c

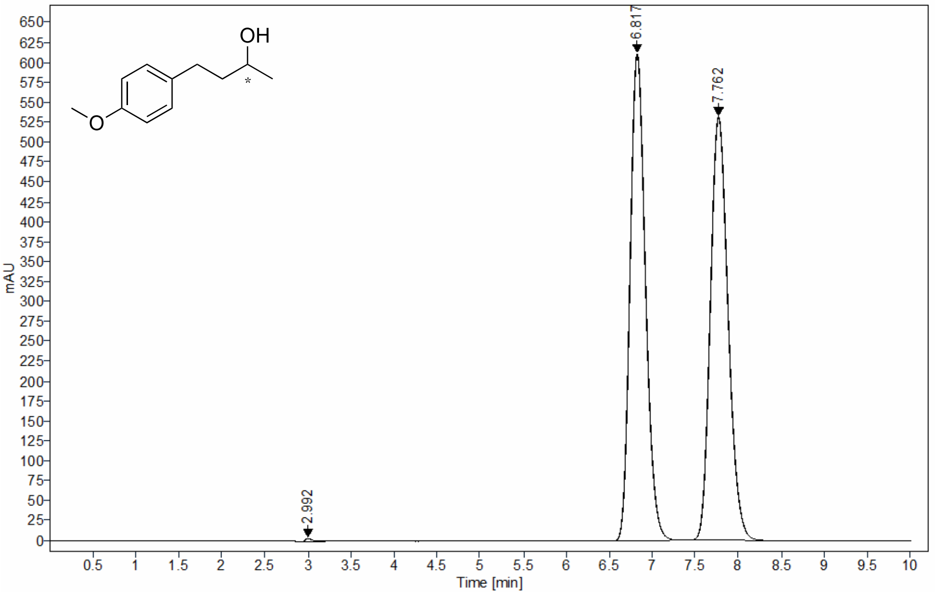

Signal:	DAD1A,Sig=225,4 Ref=off		
	RT [min]	Area	Area%
	2.992	24.7018	0.1626
	6.817	7600.0252	50.0314
	7.762	7565.7943	49.8060

Supplementary figure 84: Chiral HPLC chromatogram of the racemate 4b

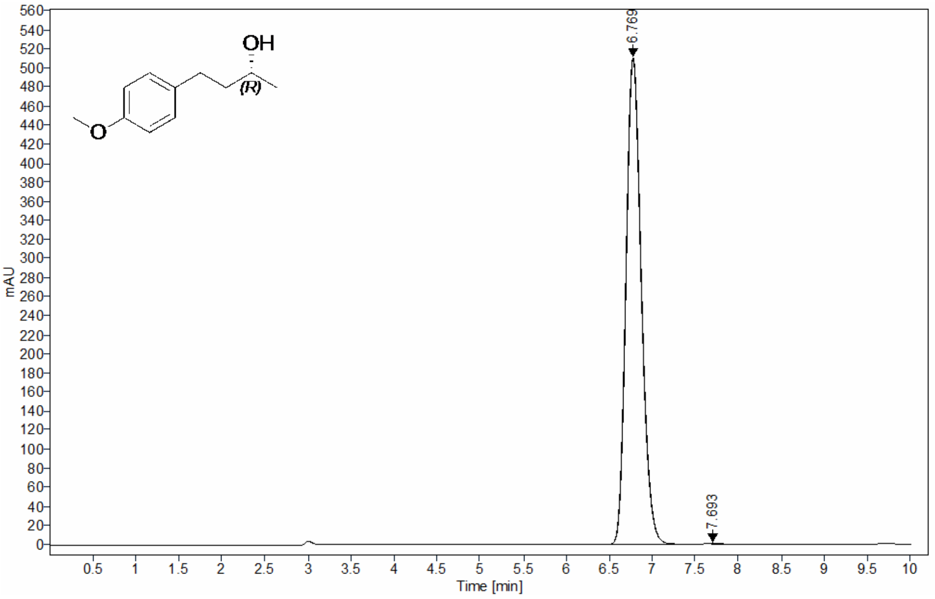

Signal:	DAD1A,Sig=225,4 Ref=off		
	RT [min]	Area	Area%
	6.769	6302.6683	99.9065
	7.693	5.9000	0.0935

Supplementary figure 85: Chiral HPLC chromatogram of the reaction catalyzed by ADH101 for 4b

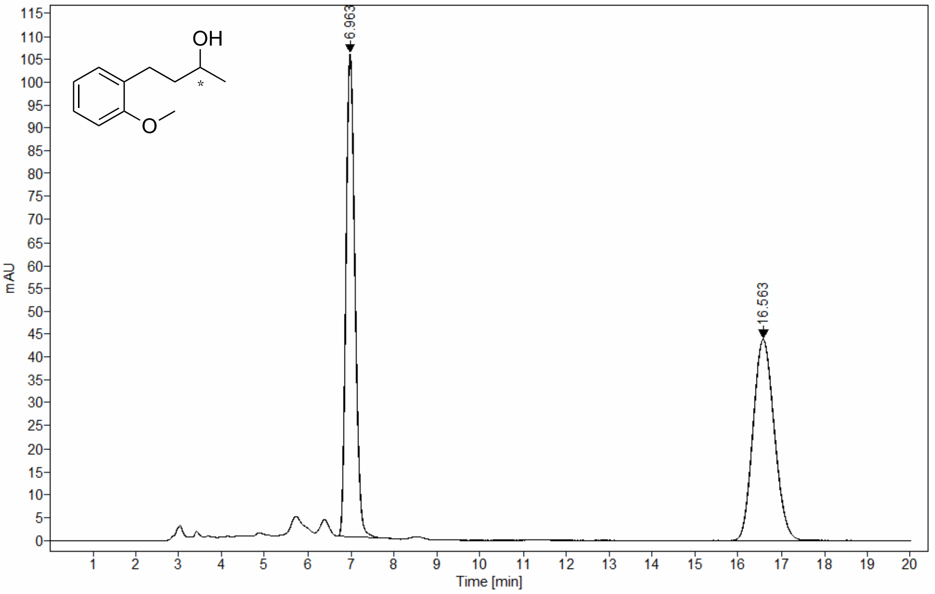

Signal:	DAD1A,Sig=270,4 Ref=off		
	RT [min]	Area	Area%
	6.963	1526.7877	50.4661
	16.563	1498.5838	49.5339

Supplementary figure 86: Chiral HPLC chromatogram of the racemate 4c

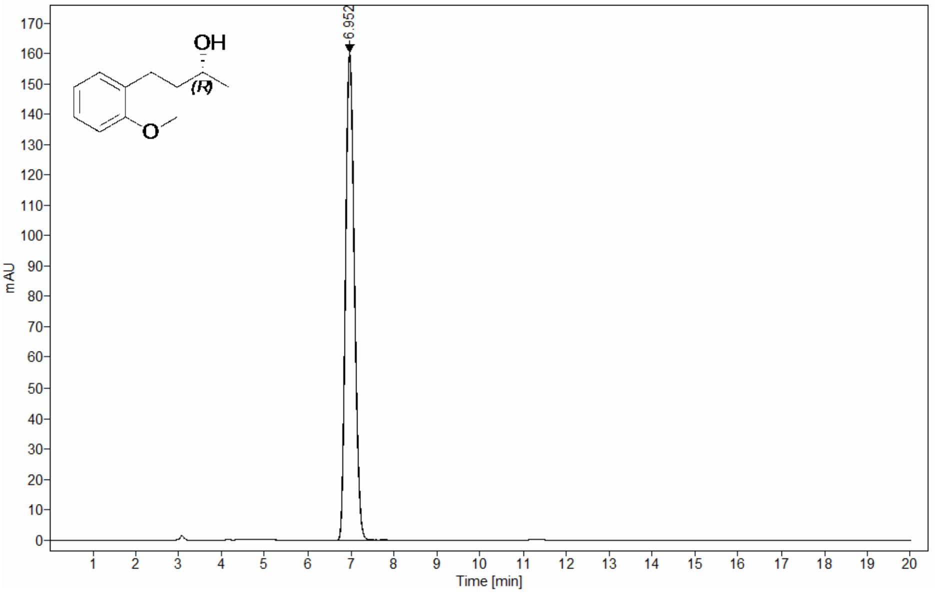

Signal:	DAD1A,Sig=270,4 Ref=off		
	RT [min]	Area	Area%
	6.952	2278.7716	100.0000

Supplementary figure 87: Chiral HPLC chromatogram of the reaction catalyzed by ADH101 for 4c

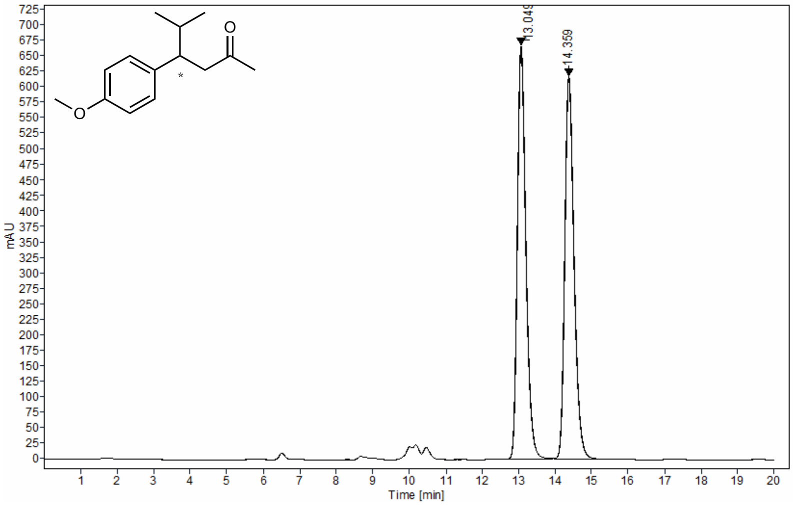

Signal:	DAD1A,Sig=210,4 Ref=off	
RT [min]	Area	Area%
13.049	10795.8985	49.9176
14.359	10831.5571	50.0824

Supplementary figure 88: Chiral HPLC chromatogram of the racemate of 4-(4-methoxyphenyl)-5-methylhexan-2-one

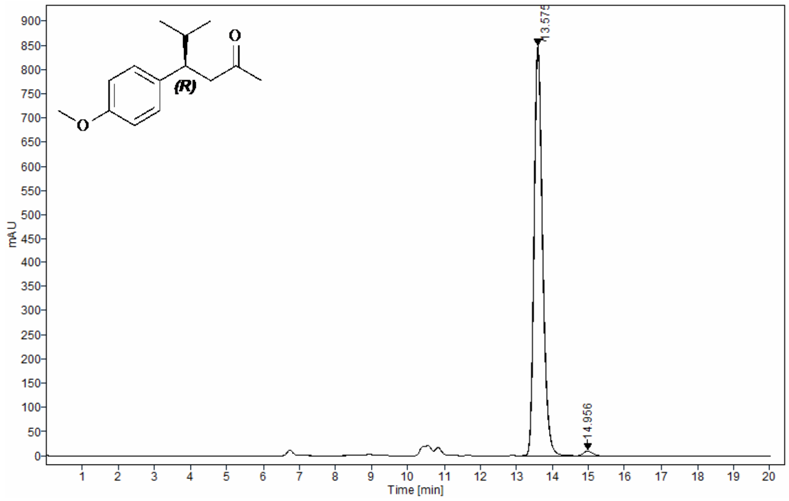

Signal:	DAD1A,Sig=210,4 Ref=off	
RT [min]	Area	Area%
13.575	13876.7652	98.7906
14.956	169.8743	1.2094

Supplementary figure 89: Chiral HPLC chromatogram of the synthesis of 4-(4-methoxyphenyl)-5-methylhexan-2-one catalyzed by (*R*-BINAP)Rh(nbd)BF_4

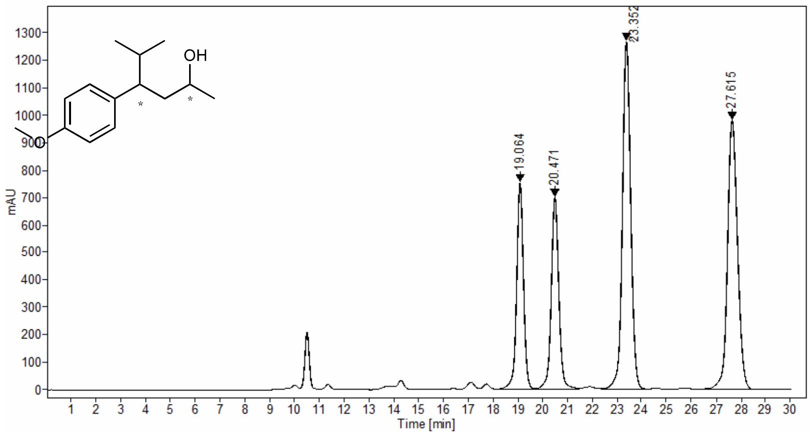

Signal:	DAD1A,Sig=210,4 Ref=off		
	RT [min]	Area	Area%
	19.064	14395.4719	16.7441
	20.471	14732.2702	17.1358
	23.352	29353.2039	34.1422
	27.615	27492.5511	31.9779

Supplementary figure 90: Chiral HPLC chromatogram of the racemate of 4-(4-methoxyphenyl)-5-methylhexan-2-ol

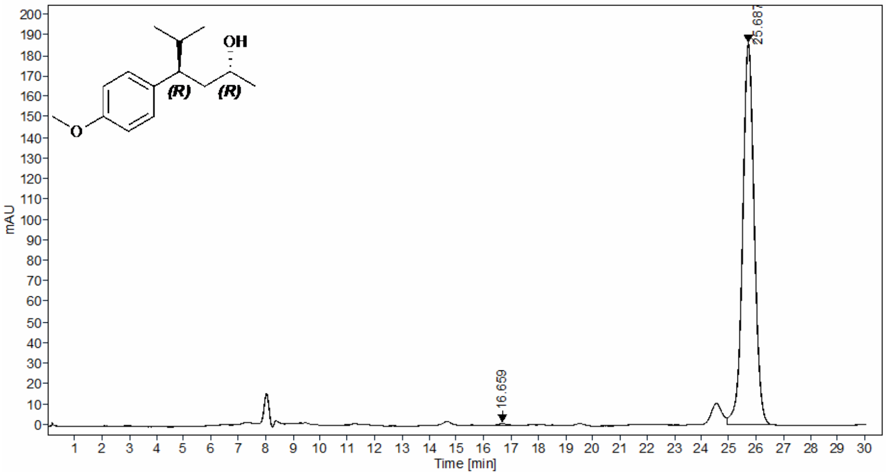

Signal:	DAD1A,Sig=210,4 Ref=off		
	RT [min]	Area	Area%
	16.659	15.3483	0.2796
	25.687	5474.2155	99.7204

Supplementary figure 91: Chiral HPLC chromatogram of the synthesis of 4-(4-methoxyphenyl)-5-methylhexan-2-ol catalyzed by (*R*-BINAP)Rh(nbd)BF_4 and ADH110

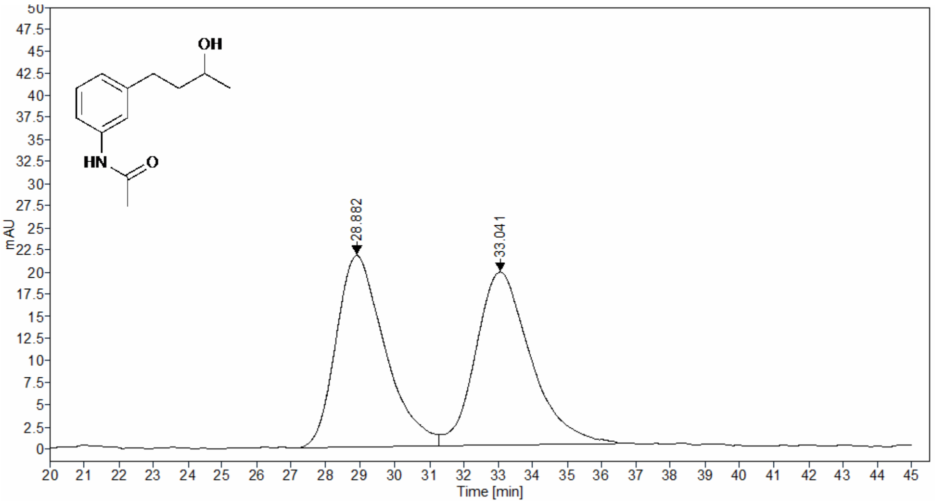

Signal:	DAD1A, Sig=210,4 Ref=off		
	RT [min]	Area	Area%
	28.882	2129.0792	49.6123
	33.041	2162.3568	50.3877

Supplementary figure 92: Chiral HPLC chromatogram of *N*-(3-(3-hydroxybutyl)phenyl)acetamide (racemic)

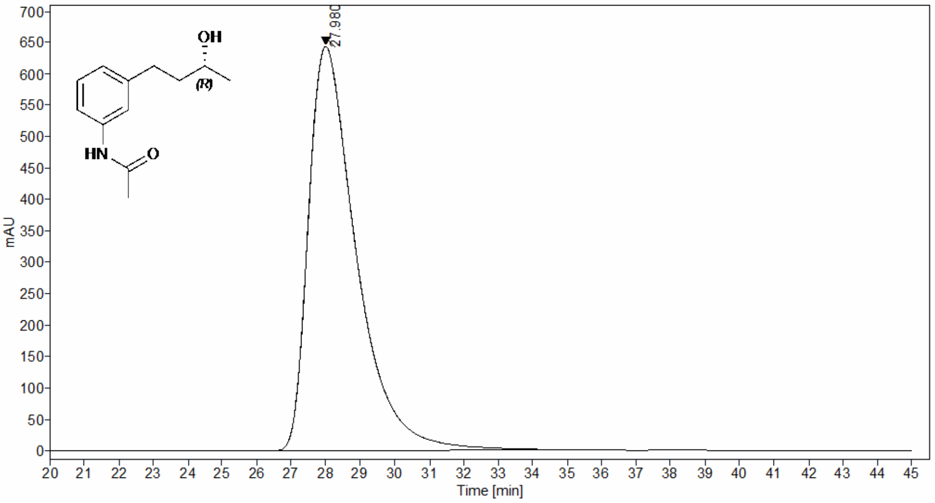

Signal:	DAD1A, Sig=210,4 Ref=off		
	RT [min]	Area	Area%
	27.980	62010.1851	100.0000

Supplementary figure 93: Chiral HPLC chromatogram of *N*-(3-(3-hydroxybutyl)phenyl)acetamide (*R*-enantiomer: catalyzed by ADH101)

Supplementary Tables

Supplementary table 1 : HPLC conditions for compounds 1-2e

No.	Compound	Enzyme	Column	Hexanes /*i*-PrOH	Flow (mL/min)	λ (nm)	t_{R1} (min)	t_{R2} (min)
**I**	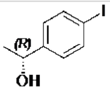	ADH101	Lux 5u Cellulose-2	97/3	1.00	232	13.98	16.14
**1a**	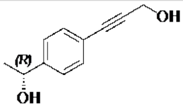	ADH105	Lux 5u Cellulose-2	80/20	1.00	242	12.79	15.23
**1a**	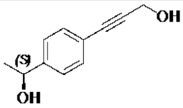	ADH112	Lux 5u Cellulose-2	80/20	1.00	242	12.79	15.23
**1b**	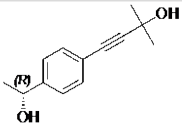	ADH101	Lux 5u Cellulose-2	80/20	1.00	242	5.96	7.28
**1c**	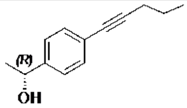	ADH101	Lux 5u Cellulose-2	98/2	1.00	242	12.09	13.43
**1d***	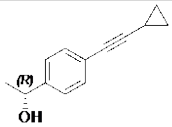	ADH101	Chiralcell ODH	95/5	1.00	245	8.25	12.67
**1e***	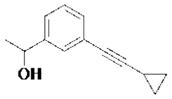	ADH101	Chiralpak AD-H	100/0 → 95/5 over 20 min	1.00	245	18.48	19.36
**2a**	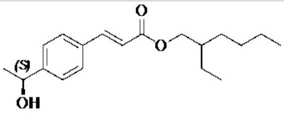	ADH112	Lux 5u Cellulose-2	90/10	1.25	278	8.61	12.18
**2b***	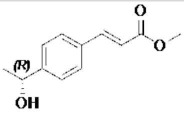	ADH101	Lux 5u Cellulose-2	90/10	1.00	275	19.52	25.07
**2c***	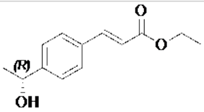	ADH101	Lux 5u Cellulose-2	90/10	1.00	275	15.25	20.87
**2d***	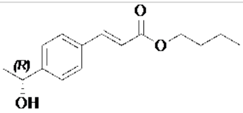	ADH101	Lux 5u Cellulose-2	90/10	1.00	275	13.23	18.62
**2e***	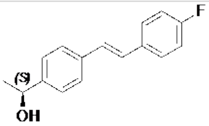	ADH101	Lux 5u Cellulose-2	90/10	1.00	275	8.436	9.337

* Acquired on an Agilent 1220 HLPC

Supplementary table 2: HPLC conditions for compounds 3a-5

**3a***	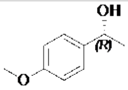	ADH101	Chiralpak AD-H	98/2	1.25	230	18.0	16.9
**3b/4a***	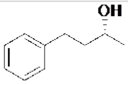	ADH101	Lux 5u Cellulose-2	95/5	1.00	210	6.76	7.82
**3c***	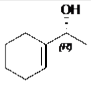	ADH101	Chiralpak AD-H	95/5	1.50	210	16.95	17.75
**4b***	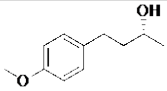	ADH101	Lux 5u Cellulose-2	95/5	1.00	210	6.76	7.82
**4c***	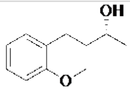	ADH101	Chiralcell ODH	90/10	1.00	205	6.96	16.56
**4d#***	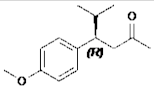	*R*-BINAP	Chiralpak AD-H	98/2	1.00	210	13.05	14.36
**4d***	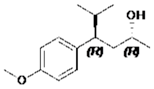	ADH110	Lux 5u Cellulose-2	95/5	1.00	210	19.06<br>20.47	23.35<br>27.62
**5***	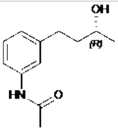	ADH101	Chiralcell ODH	90/10	1.00	210	28.88	33.04

* Acquired on an Agilent 1220 HLPC

Supplementary Methods

Preparation of the buffer solution

Aqueous 1 M stock solutions of potassium phosphate monobasic (**A**) and potassium phosphate dibasic (**B**) were prepared. A pH=7 phosphate buffer solution was then prepared by mixing 38.5 mL of solution **A** with 61.5 mL of solution **B**. The pH was controlled and adjusted, if needed, with a 1 M solution of NaOH or HCl. The buffer solution was diluted with HPLC grade water to 0.2 M. 2 wt % of TPGS-750-M, as a wax, was dissolved and used as media of the reaction. 4 and 6 wt % of TPGS-750-M in the buffer solution have also been prepared. TPGS-750-M is available from Sigma-Aldrich (catalog #733857 (solution) or #763896 (wax)). Potassium phosphate monobasic and dibasic were purchased from Sigma Aldrich.

Conversion monitoring in buffer and TPGS-750-M/buffer

ADH101,[1,2,3,] ADH105[4] ADH110[5] and ADH112[6],[7] are commercially available from the enzyme kit EZK-001 from Johnson Matthey. NAD^+ was purchased from Bioworld and $NADP^+$ from Chem-Impex. Isopropyl alcohol was purchased from VWR. All commercially available reagents were used without further purification.

To evaluate the impact of TGPS-750-M on the conversion of 4 different substrates by alcohol dehydrogenases, comparative monitoring has been performed. To a 1 dr vial were added the ketone (0.2 mmol, 1 equiv), $MgSO_4$ (0.8 mg), NAD^+ (2.6 mg) and $NADP^+$ (2.4 mg). *i*-PrOH (0.4 mL) and a [0.2M] phosphate buffer solution at pH=7 (3.2 mL) (with or without 2 wt % TPGS-750-M) were then added. ADH101 or ADH112 (20 mg) was added. The reaction was stirred at 37 °C for 24 h and monitored by HPLC (4-iodoacetophenone – calibration in **Supplementary figure 1** and **Supplementary figure 2**) or ^1H NMR (3 other examples). (Source data are provided as a Source Data file in Figures 2a-d).

Screening of surfactants

To a 1 dr vial was added 2-ethylbutyl (*E*)-3-(4-acetylphenyl)acrylate (54.9 mg, 0.2 mmol, 1 equiv), $MgSO_4$ (0.8 mg), NAD^+ (2.6 mg) and $NADP^+$ (2.4 mg). *i*-PrOH (0.4 mL) and a solution of 2 wt % of surfactant in a [0.2M] phosphate buffer at pH=7 (3.2 mL) were then added. ADH112 (20 mg) was added. The reaction was shacked at 37 °C (shaker) for 24 h. Samples were taken at different times up to 24 h, dissolved in

EtOAc and washed with distilled water. The organic layers were filtered through a pipette filled with cotton and silica gel, dried over $MgSO_4$ and concentrated in vacuo. The samples were analyzed by ^{1}H NMR to determine the conversion (2.62 ppm (s) → 1.51 ppm (d)) (Source data are provided as a Source Data file in Figure 3).

Incubation study

The stability of ADH101 in the surfactant solution has been evaluated as follow:

To a 1 dr vial was added 1-(4-(3-hydroxyprop-1-yn-1-yl)phenyl)ethan-1-one (case A: 20 mg, case B: 40 mg, case C: 80 mg, 1 equiv), $MgSO_4$ (0.8 mg), NAD^+ (2.6 mg) and $NADP^+$ (2.4 mg). *i*-PrOH (0.4 mL) and a solution of 2 wt % TPGS-750-M in a [0.2M] phosphate buffer at pH=7 (3.2 mL) were then added. ADH101 (20 mg) was added. The reaction was stirred at 37 °C for 24 h. Samples were taken at different times up to 24 h, dissolved in EtOAc and washed with distilled water. The organic layers were filtered through a pipette filled with cotton and silica gel, dried over $MgSO_4$ and concentrated in vacuo. The samples were analyzed by ^{1}H NMR to determine the conversion (2.59 ppm (s) → 1.49 ppm (d)) (Source data are provided as a Source Data file in Figures 4a-c).

Purification and characterization

Thin layer chromatography (TLC) was done using Silica Gel 60 F254 plates (Merck, 0.25 mm thick). Flash chromatography was done in glass columns using Silica Gel 60 (EMD, 40-63 µm). All the compounds prepared in this study have characterized by NMR. ^{1}H and ^{13}C NMR were recorded at 25 °C either on a Varian Unity Inova 400 MHz, a Varian Unity Inova 500 MHz or on a Varian Unity Inova 600 MHz spectrometers in $CDCl_3$ with residual $CHCl_3$ (^{1}H = 7.27 ppm, ^{13}C = 77.16 ppm) as internal standard. Chemical shifts are reported in parts per million (ppm). Data are reported as follows: chemical shift, multiplicity (s = singlet, bs = broad singlet, d = doublet, bd = broad doublet, t = triplet, q = quartet, quin = quintet, m = multiplet), coupling constant (if applicable) and integration. Chiral HPLC data were collected using either a Shimadzu LC-20AT Prominence liquid chromatograph coupled with Shimadzu SPD-M20A Prominence diode array detector or an Agilent 1220 HPLC (marked by a *). HPLC methods for each compound are described in the HPLC section of this document. HRMS data were recorded on a Waters Micromass LCT

TOF ES+ Premier mass spectrometer using ESI ionization. α-values were measured on a Perkin Elmer Polarimeter 341 in a cuvette (l=10cm) at 589 nm (Na lamp). Concentration *c* is given in g/100mL.

Circular Dichroism

The secondary structure of ADH101 has been evaluated by circular dichroism, with and without the surfactant in the buffer solution.

The circular dichroism spectra were obtained using a JASCO J-1500 spectropolarimeter (JASCO corporation, Tokyo, Japan) with a 0.1 mm pathlength, U-shaped quartz cuvette. The following conditions were employed: scanning speed 50 nm/min, band width 1 nm, and 3 accumulations per sample. CD protein spectra were corrected for the corresponding buffer or TPGS-750/buffer signal.

The amount of TPGS-750-M had to be lowered (0.03 wt % TPGS-750-M in a [0.05M] phosphate buffer at pH7) as a strong absorbance was observed in the far UV, affecting the signal of the helix portion of the protein. 4 samples have been prepared at 37 °C:

- sample 1: .2.6 mg of ADH101 in 1 mL of the 0.03 wt% TPGS-750-M/buffer solution
- sample 2: 2.6 mg of ADH101 in 1 mL of the buffer solution
- sample 3: 0.03 wt% TPGS-750-M/buffer solution (for blank)
- sample 4: buffer solution (for blank)

The analysis required lower concentration of enzymes [0.024M] than the reaction [0.056M]. The spectra is presented in **Supplementary figure 3**. (Source data are provided as a Source Data file).

Nuclear Magnetic Resonance

ADH101 ^{1}H NMR spectra has been recording in non-deuterated aqueous solutions (buffer:D_2O, 10:1) (WATERGATE, WATER suppression by Gradient-Tailored Excitation)[8] without and with TPGS-750-M at 37 °C:

- sample 1: 53.2 mg of ADH101 (0.5 mmol) was dissolved in 0.6 mL of buffer + 60 µL of D_2O.
- sample 2: 53.2 mg of ADH101 (0.5 mmol – [0.76 mol/L]) was dissolved in 0.6 mL of 2 wt % TPGS-750-Mbuffer + 60 µL of D_2O.

The analysis required higher concentration of enzymes ([0.76 mol/L] than the reaction [0.056M]. The spectra are presented in **Supplementary figure 4**, **Supplementary figure 5** and **Supplementary figure 6**.

Dynamic Light Scattering

DLS analysis of a solution of 2 wt % TPGS-750-M/buffer, of ADH101 in buffer and a solution of ADH101 in 2 wt % TPGS-750-M/buffer, all in presence of 11% *i*-PrOH, have been performed to evaluated the impact of ADH101 on micelle size. Dynamic light scattering (DLS) was measured on a Malvern Zetasizer Nano ZS. The DLS spectra is presented in **Supplementary figure 7**. (Source data are provided as a Source Data file).

Cryo-TEM images

For Cryo-TEM images, samples were prepared by plunge-freezing in liquid ethane (FEI Vitrobot Mk IV). They were then kept under LN2 before being transferred into a cryo transfer TEM holder (Gatan Single Tilt Cryo-Transfer Holder 626) and loaded into the microscope (FEI Tecnai G2 Sphera). The samples were imaged under low-dose conditions in order to minimize beam damage and images were acquired with a CCD camera (Gatan Ultrscan 1000 2k x 2k) (**Supplementary figure 8**).

Optimization of the stirring method

Optimization of the experimental conditions has been performed on the reduction of 1-(4-(3-hydroxyprop-1-yn-1-yl)phenyl)ethan-1-one. For accessibility reasons, a shaker was not the most convenient mixing method. Reactions were incubated at 37°C with shaking (250 rpm in a G10 Gyrotory Shaker from New Brunswick Scientific Co. Inc) or stirring (magnetic stir bar). To a 1 Dr vial were added 1-(4-(3-hydroxyprop-1-yn-1-yl)phenyl)ethan-1-one (34.8 mg, 0.2 mmol, 1 equiv), $MgSO_4$ (0.8 mg), NAD^+ (2.6 mg) and $NADP^+$ (2.4 mg). *i*-PrOH (0.4 mL) and a solution of 2 wt % TPGS-750-M in a [0.2M] phosphate buffer at pH=7 (3.2 mL) were then added. ADH101 (20 mg) was added. The reaction was either shacked (shaker) or stirred (magnetic bar) at 37 °C for 24 h. Samples were taken at different times up to 24 h, dissolved in EtOAc and washed with distilled water. The organic layers were filtered through a pipette filled with cotton and silica gel, dried over $MgSO_4$ and concentrated in vacuo. The samples

were analyzed by ^{1}H NMR to determine the conversion (2.59 ppm (s) → 1.49 ppm (d)).
Both conversion curves perfectly overlapped. The mixing with a stir bar did not affect the enzyme activity and was selected as mixing method for the rest of the study (**Supplementary figure. 9**). (Source data are provided as a Source Data file).

Optimization of the ratio enzyme/substrate

For a given amount of enzymatic cocktail, the amount of substrate was increased from 0.02 g [0.056M] to 0.31 g [0.5M].
To a 1 dr vial were added 1-(4-(3-hydroxyprop-1-yn-1-yl)phenyl)ethan-1-one, $MgSO_4$ (0.8 mg), NAD^+ (2.6 mg) and $NADP^+$ (2.4 mg). *i*-PrOH (0.4 mL) and a solution of 2 wt % TPGS-750-M in a [0.2M] phosphate buffer at pH=7 (3.2 mL) were then added. ADH101 (20 mg) was added. The reaction was stirred at 37 °C for 24 h. Samples were taken at different times up to 24 h, dissolved in EtOAc and washed with distilled water. The organic layers were filtered through a pipette filled with cotton and silica gel, dried over $MgSO_4$ and concentrated in vacuo. The samples were analyzed by ^{1}H NMR to determine the conversion (2.59 ppm (s) → 1.49 ppm (d)) (**Supplementary figure 10**). (Source data are provided as a Source Data file).

Optimization of the amount of *i*-PrOH

i-PrOH is used as a sacrificial reductant to regenerate NADH/NADPH. Different v/v % of *i*-PrOH have been evaluated, from 3 to 22 v/v %.
To a 1 dr vial were added 1-(4-(3-hydroxyprop-1-yn-1-yl)phenyl)ethan-1-one (34.8 mg, 0.2 mmol, 1 equiv), $MgSO_4$ (0.8 mg), NAD^+ (2.6 mg) and $NADP^+$ (2.4 mg). *i*-PrOH and a solution of 2 wt % TPGS-750-M in a [0.2M] phosphate buffer at pH = 7 were then added. ADH101 (20 mg) was added. The reaction was stirred at 37 °C for 24 h. Samples were taken at different times up to 24 h, dissolved in EtOAc and washed with distilled water. The organic layers were filtered through a pipette filled with cotton and silica gel, dried over $MgSO_4$ and concentrated in vacuo. The samples were analyzed by ^{1}H NMR to determine the conversion (2.59 ppm (s) → 1.49 ppm (d)) (**Supplementary figure 11**). (Source data are provided as a Source Data file).

Optimization of the substrate concentration

For a given amount of enzymatic cocktail and substrate, the volume of the solvent (16.7 v/v % of *i*-PrOH in 2 wt % TPGS-750-M/Buffer) has been reduced from 3.6 to 0.4 mL (from [0.056] to [0.5M]).

To a 1 dr vial were added 1-(4-(3-hydroxyprop-1-yn-1-yl)phenyl)ethan-1-one (34.8 mg, 0.2 mmol, 1 equiv), $MgSO_4$ (0.8 mg), NAD^+ (2.6 mg) and $NADP^+$ (2.4 mg). *i*-PrOH (16.7 v/v %) and a solution of 2 wt % TPGS-750-M in a [0.2M] phosphate buffer at pH=7 were then added. ADH101 (20 mg) was added. The reaction was stirred at 37 °C for 24 h and monitored by NMR ^{1}H (**Supplementary figure 12**). (Source data are provided as a Source Data file).

1-Pot Sonogashira then ADH reduction

The general procedure (condition 1) for the 1-pot Sonogashira followed by ADH reduction is: To a dried 1 dr vial were added, under an argon atmosphere, $Pd(PPh_3)_3Cl_2$ (2.8 mg, 2 mol%), CuI (1.9 mg, 5 mol%), aryl iodide (1 equiv, 0.2 mmol), alkyne (1.5 equiv, 0.3 mmol) and Et_3N (3 equiv, 84 µL). A 2 wt % TPGS-750-M/H_2O (0.4 mL) was added. The reaction was stirred at room temperature under argon until completion. The pH was adjusted to 7 with a solution of HCl 1M. The concentration was adjusted to [0.056M] by adding 2.6 mL of a 2 wt % TPGS-750-M/Buffer solution (phosphate, pH=7). MgSO4 (0.8 mg), NAD+ (2.6 mg), NADP+ (2.4 mg), *i*-PrOH (0.6 mL) and ADH101 (20 mg) were added in succession. The reaction was stirred at 37 °C until completion. The reaction was dissolved in EtOAc. The organic layer was washed with H_2O, dried over $MgSO_4$ and concentrated under vacuum. The product was purified by flash chromatography.

The general procedure (condition 2) for the 1-pot Sonogashira followed by ADH reduction is: In an over-dried, argon-flushed vial, equipped with a stir bar, was added [PdCl(cinnamyl)]2 (2.6 mg) and cBRIDP (10.6 mg) under inert atmosphere (glovebox). 2 mL of dry THF (previously degassed for 1 h) was added. The solution was stirred for 0.5 h under argon.

To a dried 1 Dr vial, under argon, was added 60 µL of the stock solution (1500 ppm). THF was flushed with argon. $K_3PO_4{\cdot}H_2O$ (2 equiv), the ketone (1 equiv) and the alkyne (1.2 equiv) were added to the vial. The vial was capped with a rubber septum.

TPGS-750-M/H_2O (0.4 mL) was added and the reaction was stirred at 45 °C under argon atmosphere for 24 h.

The concentration was adjusted to [0.056M] by adding 2.6 mL of a 2 wt % TPGS-750-M/Buffer solution (phosphate, pH=7). The pH was adjusted to 7 with a solution of HCl 1M. *i*-PrOH (0.6 mL), $MgSO_4$ (0.8 mg), NAD^+ (2.6 mg), $NADP^+$ (2.4 mg) and ADH101 (20 mg) were added in succession. The reaction was stirred at 37 °C until completion.

The reaction was dissolved in EtOAc. The organic layer was washed with H_2O, dried over $MgSO_4$ and concentrated under vacuum. The product was purified by flash chromatography.

1-Pot Heck then ADH reduction

The general procedure for the 1-pot Heck followed by ADH reduction is: To a dried 1 dr vial were added, under an argon atmosphere, $Pd(P(tBu_3))_2$ (Fu catalyst) (2.0 mg, 2 mol%), aryl iodide (1 equiv, 0.2 mmol), alkene (2 equiv, 0.4 mmol) and Et_3N (3 equiv, 84 µL). A 2 wt % TPGS-750-M/H_2O (0.4 mL) was added. The reaction was stirred at 45 °C under argon until completion. The pH was adjusted to 7 with a solution of HCl 1M. The concentration was adjusted to [0.056M] by adding 2.6 mL of a 2 wt % TPGS-750-M/Buffer solution (phosphate, pH = 7). $MgSO_4$ (0.8 mg), NAD^+ (2.6 mg), $NADP^+$ (2.4 mg), *i*-PrOH (0.6 mL) and ADH101 or ADH112 (20 mg) were added in succession. The reaction was stirred at 37 °C until completion. The reaction was dissolved in EtOAc. The organic layer was washed with H_2O, dried over $MgSO_4$ and concentrated under vacuum. The product was purified by flash chromatography.

1-Pot alkyne hydration then ADH reduction

The first step involves the Preparation of the gold pre-catalyst.[9]

(Tetrahydrothiophene)gold(I) chloride was generated from tetrahydrothiophene and auric acid according to a known procedure.2 A 10 mL round- bottom flask equipped with a Teflon-coated magnetic stir bar and septum was charged with HandaPhos (27.3 mg, 0.05 mmol) and (tetrahydrothiophene)gold(I) chloride (16.0 mg, 0.05 mmol). A rubber septum was added to the flask, which was degassed and filled with argon. The flask was covered with aluminum foil to protect it from light. Anhydrous dichloromethane (2 mL) was added via syringe and the reaction was stirred for 2 h.

After, the solvent was removed under vacuum, the product was put under high vacuum overnight to remove trace amounts of solvent and tetrahydrothiophene. A white solid was obtained (35.0 mg, 90%). 1H NMR (400 MHz, CDCl3) δ 7.53 (dd, J = 11.8, 4.3 Hz, 1H), 7.38 (t, J = 8.4 Hz, 1H), 7.01 (s, 2H), 6.91 (ddd, J = 7.4, 4.8, 2.6 Hz, 2H), 6.72 (d, J = 8.4 Hz, 1H), 6.58 (d, J = 8.4 Hz, 1H), 4.95 (dd, J = 10.3, 3.7 Hz, 1H), 3.88 (s, 3H), 3.70 (d, J = 7.6 Hz, 3H), 3.30 – 3.20 (m, 2H), 2.92 (ddt, J = 37.3, 13.8, 6.8 Hz, 3H), 1.53 (s, 2H), 1.25 (dd, J = 9.0, 3.5 Hz, 12H), 1.18 (d, J = 6.8 Hz, 6H), 0.96 (d, J = 17.2 Hz, 9H); 13C NMR (101 MHz, CDCl3) δ 163.23, 157.36, 157.08, 147.55, 147.12, 139.96, 134.26, 130.49, 128.32, 128.23, 125.65, 125.57, 121.24, 113.10, 111.68, 104.79, 103.21, 77.19, 55.84, 55.26, 34.72, 34.43, 34.09, 34.07, 33.94, 29.56, 25.95, 25.88, 24.77, 24.02, 23.93; 31P NMR (162 MHz, CDCl3) δ 59.97.

HandaPhos-gold(I) chloride (0.8 mg, 0.001 mmol) and silver(I) hexafluoroantimonate (0.7 mg, 0.002 mmol) were charged under an argon atmosphere into a 5 mL microwave vial containing a Teflon-coated magnetic stir bar and a rubber septum. The vial was covered with aluminum foil to protect the compounds from light. Anhydrous dichloromethane (1 mL) was added via syringe and the reaction was stirred for 15-20 min prior to use.

The gold pre-catalyst is used to catalyzed the Alkyne hydration step of the following 1-pot, 2-step reaction:
To a dried 1 dr vial was added, under an argon atmosphere, 0.2 mL of the gold pre-catalyst solution (1000 ppm or 0.1 mol %). Dichloromethane was evaporated under Argon. Alkyne (0.2 mmol, 1.0 equiv) was added to the vial, followed by toluene (20 µL), a 3 wt % TPGS-750-M/H_2O solution (0.2 mL, 1.0M), and trifluoroacetic acid (46 mg, 0.4 mmol), 2.0 equiv). The resulting mixture was stirred at rt for 24 h. The pH was adjusted to 7 with a solution of NaOH 1M. The concentration was adjusted to [0.056M] by adding 2.6 mL of a 2 wt v/v % TPGS-750-M/Buffer solution (phosphate, pH = 7) and *i*-PrOH (0.6 mL). $MgSO_4$ (0.8 mg), NAD^+ (2.6 mg), $NADP^+$ (2.4 mg) and ADH101 (20 mg) were added in succession. The reaction was stirred at 37 °C until completion. The reaction was dissolved in EtOAc. The organic layer was washed with H_2O, dried over $MgSO_4$ and concentrated under vacuum. The product was purified by flash chromatography.

1-Pot 1,4-addition then ADH reduction

To an oven-dried 1 dr vial was added, under inert atmosphere (glovebox) $Rh(nbd)_2BF_4$ (11.2 mg) and BINAP (18.7 mg). Dry dichloromethane (1 mL) was added under Argon and the solution was stirred for 1 min.

To a dried 1 dr vial was added, under an argon atmosphere, 0.2 mL of the stock solution (3 mol %). Dichloromethane was evaporated under argon. Boronic acid (0.2 mmol, 1.0 equiv), followed by a 2 wt % TPGS-750-M/H_2O solution (0.4 mL, [0.5 M]) and TEA (84 µL, 0.6 mmol, 3.0 equiv) were added in succession. The reaction was stirred for 15 min until homogeneous. Vinyl ketone was then added (0.2 mmol, 1.0 equiv). The reaction was stirred 12 h at room temperature. The concentration was adjusted to [0.056M] by adding 2.6 mL of a 2 wt % TPGS-750-M/Buffer solution (phosphate, pH = 7) and *i*-PrOH (0.6 mL).

Note: at this stage, the pH of the solution is 7. There is no need to adjust the pH for this reaction.

$MgSO_4$ (0.8 mg), NAD^+ (2.6 mg), $NADP^+$ (2.4 mg) and ADH101 (20 mg) were added in succession. The reaction was stirred at 37 °C until completion. The reaction was dissolved in EtOAc. The organic layer was washed with H_2O, dried over $MgSO_4$ and concentrated under vacuum. The product was purified by flash chromatography.

1-Pot, 3-step reaction

To a 15 mL round-bottom flask was added, under argon, $Rh(nbd)_2BF_4$ (4.9 mg, 3 mol%) and *rac*-BINAP (8.2 mg, 3 mol%) in dichloromethane (1 mL). The solution was stirred for 5 min and the solvent was removed under argon. 3-nitrophenylboronic acid (91.8 mg, 0.44 mmol, 1.0 equiv) was added, followed by a 2 wt % solution of TPGS-750-M/H_2O and TEA (184 µL, 1.32 mmol, 3.0 equiv). After 15 min, methyl vinyl ketone (37 µL, 0.44 mmol, 1.0 equiv) was added and the reaction was stirred at 45 °C for 12 h. After completion of the first step, Carbonyl Iron Powder (CIP – 73.7 mg, 1.3 mmol, 3.0 equiv) and NH_4Cl (117.7 mg, 2.2 mmol, 5.0 equiv) were added. HCl 12M (37 µL, 0.44 mmol, 1.0 equiv) was added and the reaction was stirred under argon overnight. After completion of the second step, the reaction was diluted with a 2 wt % TPGS-750-M/buffer solution (6.2 mL) and *i*-PrOH (1.3 mL). The pH was adjusted to 7 with a 1M HCl solution. $MgSO_4$ (1.8 mg), NAD^+ (5.7 mg), $NADP^+$ (5.3

mg), and ADH101 (44 mg) were added as solids. The reaction was stirred at 37 °C for 2 h. After completion, the pH of the aqueous solution was adjusted to 10 and the product was extracted with EtOAc. The organic layer was dried over $MgSO_4$, filtered and concentrated *in vacuo*. The product was purified by flash chromatography (100:0 to 50:50 Hexanes/EtOAc).

Industrial scale up

To a 2 wt% solution of TPGS-750-M in phosphate buffer (pH=7.05, 10 v) was added glucose (2.0 equiv) in a mechanically stirred reactor equipped with pH controller at 25 °C. The suspension was further stirred for 20 minutes, and to the resulting mixture was sequentially added $NADP^+$ (1.2 wt %), GDH (1.0 wt %) and KRED-EW-124 (5 wt %). The ketone (3.0 kg, 8.2 mol, 1.0 equiv) was added and the pH of the reaction mixture was adjusted to 6.8-7.2 by addition of 1 M aqueous NaOH at 25 °C. The resulting reaction mixture was heated to 40 °C and stirred for 24 h until completion of the reaction as determined by HPLC. As the reaction proceeded, the product precipitated out from the reaction mixture and formed a suspension. The resulting suspension was filtered at 40 °C, and the resulting wet cake was washed with water and dried to give product **6** as an off-white solid (3.5 kg, purity 97%, yield 85%).

Supplementary Notes

Supplementary Note 1: (*R*)-1-(4-iodophenyl)ethan-1-ol

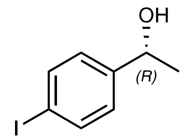

^{1}H NMR (500 MHz, $CDCl_3$) δ 7.71 – 7.65 (m, 2H), 7.17 – 7.11 (m, 2H), 4.87 (qd, *J* = 6.4, 3.5 Hz, 1H), 1.80 (d, *J* = 3.7 Hz, 1H), 1.48 (d, *J* = 6.5 Hz, 3H); **^{13}C NMR** (126 MHz, $CDCl_3$) δ 145.6, 137.7, 127.5, 92.8, 70.0, 25.4; **R enantiomer:** $\boldsymbol{a_D^{20.0}}$= + 19.96 (*c*0.867 in $CHCl_3$) (ADH101); **S enantiomer:** $\boldsymbol{a_D^{20.0}}$= - 30.64 (*c*0.780 in $CHCl_3$) (ADH112); TLC (Hexanes:EtOAc, 1:1 v/v): **Rf** = 0.71.

Supplementary Note 2: (*R*)-1-(4-(trifluoromethyl)phenyl)ethan-1-ol

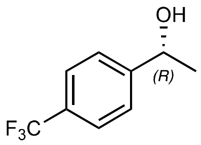

^{1}H NMR (500 MHz, $CDCl_3$) δ 7.61 (d, *J* = 8.1 Hz, 2H), 7.49 (d, *J* = 8.0 Hz, 2H), 4.97 (q, *J* = 6.5 Hz, 1H), 2.00 (t, *J* = 9.9 Hz, 1H), 1.51 (d, *J* = 6.5 Hz, 3H); **^{13}C NMR** (126 MHz, $CDCl_3$) δ 149.8, 129.7, 125.8, 125.6, 125.6, 125.6, 125.5, 125.4, 123.2, 70.0, 25.5; TLC (Hexanes:EtOAc, 1:1 v/v): **Rf** = 0.68.

Supplementary Note 3: 2-ethylbutyl (*S,E*)-3-(4-(1-hydroxyethyl)phenyl)acrylate

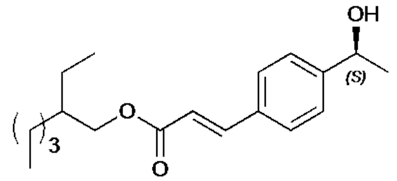

^{1}H NMR (600 MHz, $CDCl_3$) δ = 7.66 (d, J=16.0, 1H), 7.52 (d, J=8.1, 2H), 7.40 (d, J=8.1, 2H), 6.43 (d, J=16.0, 1H), 4.93 (qd, J=6.4, 3.2, 1H), 4.17 – 4.08 (m, 2H), 1.98 (d, J=3.5, 1H), 1.71 – 1.61 (m, 1H), 1.51 (d, J=6.5, 3H), 1.49 – 1.38 (m, 2H), 1.38 – 1.28 (m, 6H), 0.92 (dt, J=10.8, 7.1, 6H); **^{13}C NMR** (126 MHz, $CDCl_3$) δ 167.4, 148.4, 144.3, 133.6, 128.3, 126.0, 118.1, 70.0, 67.1, 38.9, 30.6, 29.0, 25.3, 23.9, 23.1, 14.1, 11.1; $\boldsymbol{a_D^{20.0}}$= - 16.83 (*c*0.873 in $CHCl_3$) (ADH112); TLC (1:1 hexanes:EtOAc, 1:1 v/v): **Rf** = 0.59.

Supplementary Note 4: (*R,E*)-4-phenylbut-3-en-2-ol

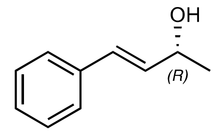

^{1}H NMR (500 MHz, $CDCl_3$) δ 7.42 – 7.37 (m, 2H), 7.33 (t, *J* = 7.6 Hz, 2H), 7.27 – 7.23 (m, 1H), 6.58 (dd, *J* = 15.9, 1.2 Hz, 1H), 6.28 (dd, *J* = 15.9, 6.4 Hz, 1H), 4.50 (pd, *J* = 6.4, 1.2 Hz, 1H), 1.83 – 1.59 (m, 1H), 1.39 (d, *J* = 6.4 Hz, 3H); **^{13}C NMR** (126 MHz, $CDCl_3$) δ 136.8, 133.7, 129.5, 128.7, 127.8, 126.6, 69.1, 69.1, 23.6; TLC (Hexanes:EtOAc, 1:1 v/v): **Rf** = 0.68.

Supplementary Note 5: (*R*)-3-(4-(1-hydroxyethyl)phenyl)prop-2-yn-1-ol (1a)

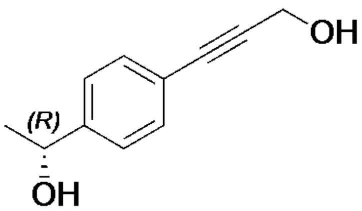

0.2 mmol scale (0.0349 g) – Yield = 99% - ee > 99%
pale yellow powder
^{1}H NMR (500 MHz, $CDCl_3$) δ = 7.45 – 7.39 (m, 2H), 7.38 – 7.30 (m, 2H), 4.91 (qd, *J*=6.5, 2.6, 1H), 4.50 (d, *J*=5.9, 2H), 1.89 (d, *J*=3.3, 1H), 1.80 (t, *J*=6.1, 1H), 1.49 (d, *J*=6.5, 3H); **^{13}C NMR** (101 MHz, $CDCl_3$) δ = 146.3, 132.0, 125.5, 121.7, 87.2, 85.7, 70.2, 51.8, 25.3; $\boldsymbol{a_D^{20.0}}$= + 54.05 (*c*0.740 in $CHCl_3$); TLC (hexanes:EtOAc, 1:1, v/v): **Rf** = 0.45; **HRMS (m/z): [M]$^+$** calcd. for $C_{11}H_{12}O_2$, 176.0837; found, 176.0840.

Supplementary Note 6: (*R*)-4-(4-(1-hydroxyethyl)phenyl)-2-methylbut-3-yn-2-ol (1b)

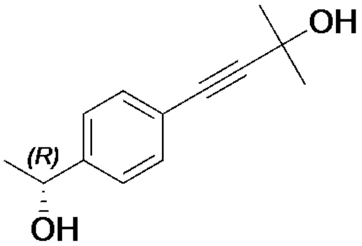

0.2 mmol scale (0.0404g) – Yield = 99% - ee > 99%
yellow powder
^{1}H NMR (400 MHz, $CDCl_3$) δ 7.39 (dt, *J* = 8.3, 2.2 Hz, 2H), 7.30 (dd, *J* = 8.3, 2.5 Hz, 2H), 4.94 – 4.81 (m, 1H), 2.25 (bs, 1H), 2.04 (bs, 1H), 1.71 (bs, 0.5H), 1.62 (s, 6H), 1.47 (d, *J* = 6.4 Hz, 3H); **^{13}C NMR** (101 MHz, $CDCl_3$) δ 146.0, 131.9, 125.4, 121.9, 93.8, 82.1, 70.2, 65.7, 31.6, 25.3; $a_D^{20.0}$= + 54.29 (*c*1.387 in $CHCl_3$); TLC (hexanes:EtOAc, 1:1 v/v): **Rf** = 0.28; **HRMS (m/z): [M-H_2O]$^+$** calcd. for $C_{13}H_{14}O$, 186.1045; found, 186.1042.

Supplementary Note 7: (*R*)-1-(4-(pent-1-yn-1-yl)phenyl)ethan-1-ol (1c)

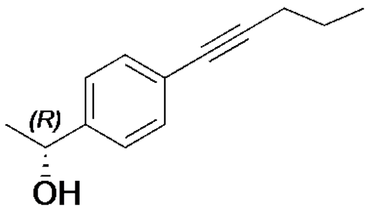

0.2 mmol scale (0.0297g) – Yield = 79% - ee > 99%

brown powder

^{1}H NMR (600 MHz, $CDCl_3$) δ 7.42 – 7.36 (m, 2H), 7.29 (d, *J* = 8.2 Hz, 2H), 4.88 (qt, *J* = 9.1, 4.8 Hz, 1H), 2.39 (td, *J* = 7.1, 1.1 Hz, 2H), 1.84 (d, *J* = 3.3 Hz, 1H), 1.69 – 1.59 (m, 2H), 1.48 (dd, *J* = 6.4, 1.3 Hz, 3H), 1.06 (td, *J* = 7.4, 1.1 Hz, 3H); **^{13}C NMR** (101 MHz, $CDCl_3$) δ 145.2, 137.6, 131.7, 127.5, 125.3, 123.2, 90.3, 80.6, 70.2, 25.3, 25.2, 22.3, 21.5, 13.7; $a_D^{20.0}$= + 20.07 (*c*0.847 in $CHCl_3$); TLC (hexanes:EtOAc 1:1 v/v): **Rf**= 0.55.

Supplementary Note 8: (*R*)-1-(4-(cyclopropylethynyl)phenyl)ethan-1-ol (1d)

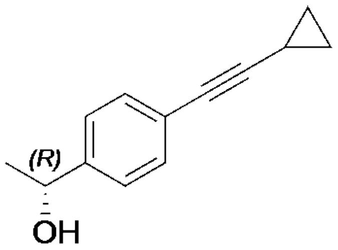

0.2 mmol scale (0.0308g) – Yield = 77% - > 99.9% ee

Off-white powder

^{1}H NMR (500 MHz, $CDCl_3$) δ 7.36 (d, *J* = 8.2 Hz, 2H), 7.26 (d, *J* = 8.1 Hz, 2H), 4.85 (q, *J* = 6.5 Hz, 1H), 2.06 – 1.97 (m, 2H), 1.46 (d, *J* = 6.6 Hz, 3H), 0.93 – 0.84 (m, 2H), 0.84 – 0.77 (m, 2H); **^{13}C NMR** (126 MHz, $CDCl_3$) δ 145.2, 131.8, 125.3, 123.1, 93.5, 75.7, 70.2, 25.2, 8.7, 0.3; $a_D^{20.0}$= + 56.27 (*c*0.773 in $CHCl_3$); TLC (hexanes:EtOAc, 8:1 v/v): **Rf** = 0.31; **HRMS (m/z): $[M]^+$** calcd. for $C_{13}H_{14}O$, 186.1045; found, 186.1045.

Supplementary Note 9: (*R*)-1-(3-(cyclopropylethynyl)phenyl)ethan-1-ol (1e)

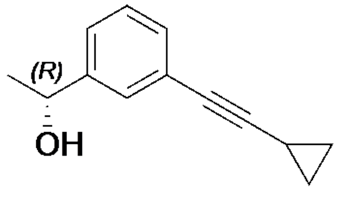

0.2 mmol scale (0.0254g) – Yield = 68% - > 99.5% ee

Pale yellow oil

^{1}H NMR (500 MHz, $CDCl_3$) δ 7.40 (d, *J* = 1.9 Hz, 1H), 7.31 – 7.20 (m, 3H), 4.85 (q, *J* = 6.4 Hz, 1H), 1.92 (s, 1H), 1.48 (d, *J* = 6.5 Hz, 3H), 0.92 – 0.84 (m, 2H), 0.84 – 0.78 (m, 2H); **^{13}C NMR** (126 MHz, $CDCl_3$) δ 146.0, 130.8, 128.8, 128.5, 124.7, 93.6, 75.8, 70.2, 25.2, 8.7, 0.3; $a_D^{20.0}$= + 38.98 (*c*0.947 in $CHCl_3$); TLC (hexanes:EtOAc, 8:1 v/v): **Rf** = 0.38; **HRMS (m/z): $[M]^+$** calcd. for $C_{13}H_{14}O$, 186.1045; found, 186.1043.

Supplementary Note 10: 2-ethylhexyl (*E*)-3-(4-((*S*)-1-hydroxyethyl)phenyl)acrylate (2a)

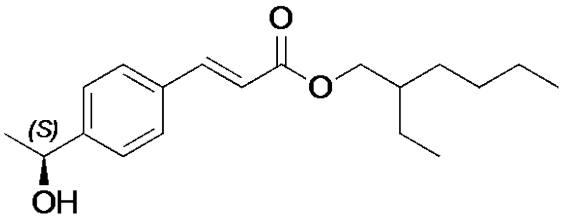

0.2 mmol scale (0.0499g) - Yield = 82% - ee > 99% - *yellow oil*

^{1}H NMR (600 MHz, $CDCl_3$) δ = 7.66 (d, *J*=16.0, 1H), 7.52 (d, *J*=8.1, 2H), 7.40 (d, *J*=8.1, 2H), 6.43 (d, *J*=16.0, 1H), 4.93 (qd, *J*=6.4, 3.2, 1H), 4.17 – 4.08 (m, 2H), 1.98 (d, *J*=3.5, 1H), 1.71 – 1.61 (m, 1H), 1.51 (d, *J*=6.5, 3H), 1.49 – 1.38 (m, 2H), 1.38 – 1.28 (m, 6H), 0.92 (dt, *J*=10.8, 7.1, 6H); **^{13}C NMR** (126 MHz, $CDCl_3$) δ 167.4, 148.4, 144.3, 133.6, 128.3, 126.0, 118.1, 70.0, 67.1, 38.9, 30.6, 29.0, 25.3, 23.9, 23.1, 14.1, 11.1; $\boldsymbol{\alpha_D^{20.0}}$= - 16.83 (*c*0.873 in $CHCl_3$) (ADH112); TLC (hexanes:EtOAc, 1:1 v/v): **Rf** = 0.59; **HRMS (m/z): [M-H_2O]$^+$** calcd. for $C_{19}H_{26}O_2$, 286.1933; found, 286.1934.

Supplementary Note 11: methyl (*R,E*)-3-(4-(1-hydroxyethyl)phenyl)acrylate (2b)

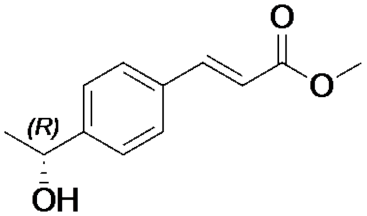

0.2 mmol scale (32.2 mg) – Yield = 78% – >99.9% ee
pale yellow oil

^{1}H NMR (600 MHz, $CDCl_3$) δ 7.68 (d, *J* = 16.0 Hz, 1H), 7.54 – 7.47 (m, 2H), 7.42 – 7.37 (m, 2H), 6.42 (d, *J* = 16.0 Hz, 1H), 4.92 (q, *J* = 6.5 Hz, 1H), 3.81 (s, 3H), 2.04 (bs, 1H), 1.50 (d, *J* = 6.5 Hz, 3H); **^{13}C NMR** (126 MHz, $CDCl_3$) δ 167.6, 148.4, 144.6, 133.7, 128.4, 126.0, 117.7, 70.1, 51.8, 25.3; $\alpha_D^{20.0}$= +23.3 (*c*0.593 in $CHCl_3$); TLC (hexanes:EtOAc, 7:3 v/v): **Rf** = 0.3.

Supplementary Note 12: ethyl (*R,E*)-3-(4-(1-hydroxyethyl)phenyl)acrylate (2c)

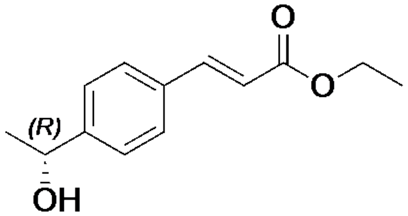

0.2 mmol scale (36.6 mg) – Yield = 83% – >99.9% ee
pale yellow oil

^{1}H NMR (600 MHz, $CDCl_3$) δ 7.68 (d, *J* = 16.0 Hz, 1H), 7.55 – 7.49 (m, 2H), 7.44 – 7.37 (m, 2H), 6.43 (d,

J = 16.0 Hz, 1H), 4.93 (q, *J* = 6.5 Hz, 1H), 4.27 (q, *J* = 7.1 Hz, 2H), 1.89 (bs, 1H), 1.51 (d, *J* = 6.5 Hz, 3H), 1.35 (t, *J* = 7.1 Hz, 3H); ^{13}**C NMR** (126 MHz, $CDCl_3$) δ 167.2, 148.2, 144.3, 133.8, 128.4, 126.0, 118.3, 70.2, 60.7, 25.3, 14.5; $\boldsymbol{a_D^{20.0}}$= +40.2 (*c*1.264 in $CHCl_3$); TLC (hexanes:EtOAc, 7:3 v/v): **Rf** = 0.3; **HRMS (m/z): [M]**$^+$ calcd. for $C_{13}H_{16}O_3$, 220.1099; found, 220.1101.

Supplementary Note 13: butyl (*R,E*)-3-(4-(1-hydroxyethyl)phenyl)acrylate (2d)

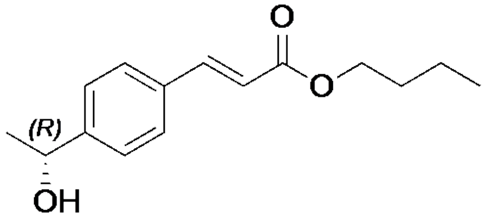

0.2 mmol scale (35.3 mg) – Yield = 71% – >99.9% ee

pale yellow oil

^{1}**H NMR** (600 MHz, $CDCl_3$) δ 7.67 (d, *J* = 16.0 Hz, 1H), 7.55 – 7.48 (m, 2H), 7.43 – 7.37 (m, 2H), 6.43 (d, *J* = 16.0 Hz, 1H), 4.93 (q, *J* = 6.5 Hz, 1H), 4.21 (t, *J* = 6.7 Hz, 2H), 1.99 (bs, 1H), 1.73 – 1.66 (m, 2H), 1.51 (d, *J* = 6.5 Hz, 3H), 1.49 – 1.40 (m, 2H), 0.97 (t, *J* = 7.4 Hz, 3H); ^{13}**C NMR** (126 MHz, $CDCl_3$) δ 167.3, 148.2, 144.3, 133.8, 128.4, 126.0, 118.2, 70.2, 64.6, 30.9, 25.3, 19.3, 13.9; $a_D^{20.0}$= +34.3 (*c*2.136 in $CHCl_3$); TLC (hexanes:EtOAc, 7:3 v/v): **Rf** = 0.32.

Supplementary Note 14: (*S,E*)-1-(4-(4-fluorostyryl)phenyl)ethan-1-ol (2e)

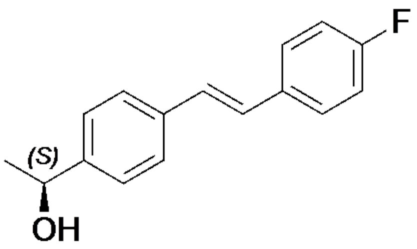

0.2 mmol scale (42.2 mg) – Yield = 87% – >99.9% ee

white powder

^{1}**H NMR** (600 MHz, $CDCl_3$) δ 7.52 – 7.46 (m, 4H), 7.41 – 7.36 (m, 2H), 7.10 – 6.99 (m, 4H), 4.93 (qd, *J* = 6.4, 3.0 Hz, 1H), 1.81 (d, *J* = 3.5 Hz, 1H), 1.53 (d, *J* = 6.5 Hz, 3H); ^{13}**C NMR** (126 MHz, $CDCl_3$) δ 163.5, 161.5, 145.4, 136.6, 133.7, 128.1, 128.1, 127.6, 126.7, 125.9, 115.9, 115.7, 70.3, 25.3; $a_D^{20.0}$= +18+8 (*c*0.529 in $CHCl_3$); TLC (hexanes:EtOAc, 7.5:2.5 v/v): **Rf** = 0.28; **HRMS (m/z): [M-H_2O]**$^+$ calcd. for $C_{16}H_{13}F$, 224.1001; found, 224.1005.

Supplementary Note 15: (*R*)-1-(4-methoxyphenyl)ethan-1-ol (3a)

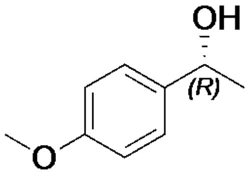

0.2 mmol scale (28.0 mg) – Yield = 92% – >99.9% ee

yellow oil

^{1}H NMR (500 MHz, $CDCl_3$) δ 7.34 – 7.29 (m, 2H), 6.92 – 6.87 (m, 2H), 4.87 (qd, J = 6.5, 1.7 Hz, 1H), 3.81 (d, J = 0.8 Hz, 3H), 1.83 (d, J = 26.1 Hz, 1H), 1.49 (dd, J = 6.4, 1.1 Hz, 3H); **^{13}C NMR** (126 MHz, $CDCl_3$) δ 159.1, 138.1, 126.8, 114.0, 70.1, 55.4, 25.2; $a_D^{20.0}$= +18.0 (*c*0.421 in $CHCl_3$); TLC (hexanes:EtOAc, 1:1 v/v): **Rf** = 0.57.

Supplementary Note 16: (*R*)-4-phenylbutan-2-ol (3b)

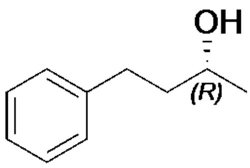

0.2 mmol scale (29.8 mg) – Yield = 99% – >99.9% ee

colorless oil

^{1}H NMR (500 MHz, $CDCl_3$) δ 7.30 (td, J = 7.4, 1.4 Hz, 2H), 7.24 – 7.17 (m, 3H), 3.89 – 3.79 (m, 1H), 2.77 (ddd, J = 13.7, 9.3, 6.3 Hz, 1H), 2.69 (ddd, J = 13.7, 9.2, 7.0 Hz, 1H), 1.86 – 1.72 (m, 2H), 1.45 (s, 1H), 1.25 (d, J = 6.2 Hz, 3H); **^{13}C NMR** (126 MHz, $CDCl_3$) δ 142.2, 128.5, 126.0, 67.7, 41.0, 32.3, 23.8; $a_D^{20.0}$= -16.3 (*c*1.100 in $CHCl_3$); TLC (hexanes:EtOAc, 8:2 v/v): **Rf** = 0.33.

Supplementary Note 17: (*R*)-1-(cyclohex-1-en-1-yl)ethan-1-ol (3c)

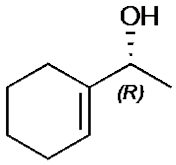

0.2 mmol scale (22.8 mg) – Yield = 90% – >99.0% ee

pale yellow oil

^{1}H NMR (500 MHz, $CDCl_3$) δ 5.68 (tdd, *J* = 3.4, 2.6, 1.5 Hz, 1H), 4.17 (q, *J* = 6.5 Hz, 1H), 2.11 – 1.93 (m, 4H), 1.72 – 1.50 (m, 5H), 1.41 – 1.35 (m, 1H), 1.27 (d, *J* = 6.5 Hz, 3H); **^{13}C NMR** (126 MHz, $CDCl_3$) δ 141.4, 121.7, 72.3, 25.0, 23.8, 22.8, 22.8, 21.7; $\mathbf{a_D^{20.0}}$= +1.9 (c0.667 in CHCl3); TLC (hexanes:EtOAc, 8:2 v/v): **Rf** = 0.38.

Supplementary Note 18: (*R*)-4-phenylbutan-2-ol (4a)

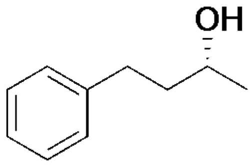

0.2 mmol scale (24.6 mg) – Yield = 82% – >99.8% ee

characterization: cf. **3b**

Supplementary Note 19: (*R*)-4-(4-methoxyphenyl)butan-2-ol (4b)

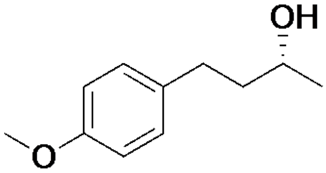

0.2 mmol scale (31.9 mg) – Yield = 88% – >99.9%ee

yellow oil

^{1}H NMR (500 MHz, $CDCl_3$) δ 7.15 – 7.10 (m, 2H), 6.86 – 6.82 (m, 2H), 3.87 – 3.81 (m, 1H), 3.80 (s, 3H), 2.75 – 2.58 (m, 2H), 1.82 – 1.68 (m, 2H), 1.26 (s, 1H), 1.23 (d, J = 6.2 Hz, 3H); **^{13}C NMR** (126 MHz, $CDCl_3$) δ 157.9, 134.2, 129.4, 114.0, 67.7, 55.4, 41.2, 31.4, 23.8; $a_D^{20.0}$= -5.3 (*c*0.414 in $CHCl_3$); TLC (Hexanes:EtOAc, 8:2 v/v): **Rf** = 0.28.

Supplementary Note 20: (*R*)-4-(2-methoxyphenyl)butan-2-ol (4c)

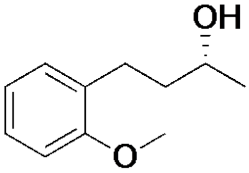

0.2 mmol scale (28.7 mg) – Yield = 80% – >99.9% ee

colorless oil

^{1}H NMR (500 MHz, $CDCl_3$) δ 7.22 – 7.14 (m, 2H), 6.95 – 6.85 (m, 2H), 3.85 (s, 3H), 3.73 (dqd, J = 8.1, 6.2, 4.5 Hz, 1H), 2.79 (dt, J = 13.6, 8.1 Hz, 1H), 2.69 (ddd, J = 13.6, 8.1, 5.4 Hz, 1H), 1.80 – 1.66 (m, 2H), 1.27 (s, 1H), 1.20 (d, J = 6.2 Hz, 3H); **^{13}C NMR** (126 MHz, $CDCl_3$) δ 130.2, 127.3, 120.9, 110.5, 67.2, 55.5, 39.9, 26.2, 23.3; $a_D^{20.0}$= -9.8 (*c*0.579 in $CHCl_3$); TLC (hexanes:EtOAc, 8:2 v/v): **Rf** = 0.28.

Supplementary Note 21: (2*R*,4*R*)-4-(4-methoxyphenyl)-5-methylhexan-2-ol (4d)

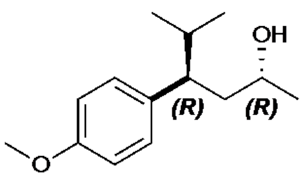

0.2 mmol scale (38.7 mg) – Yield = 87% – >99.9% ee - > 99.4% de (0.6% of (2*R*)-(4*S*)) – *white solid*

^{1}H NMR (500 MHz, $CDCl_3$) δ 7.10 – 7.02 (m, 2H), 6.88 – 6.80 (m, 2H), 3.80 (s, 3H), 3.56 (i-PrOH, J = 6.2 Hz, 1H), 2.26 (ddd, J = 11.4, 7.4, 4.7 Hz, 1H), 1.94 – 1.71 (m, 3H), 1.33 – 1.22 (bs, 1H), 1.13 (d, J = 6.1 Hz, 3H), 0.93 (d, J = 6.7 Hz, 3H), 0.71 (d, J = 6.7 Hz, 3H); **^{13}C NMR** (126 MHz, $CDCl_3$) δ 158.1, 135.6, 129.4, 113.7, 67.5, 55.3, 49.7, 42.9, 34.0, 31.7, 23.0, 22.8, 21.1, 20.4, 14.3; $\boldsymbol{a_D^{20.0}}$= -1.2 (*c*0.310 in $CHCl_3$); TLC (hexanes:EtOAc, 8:2 v/v): **Rf** = 0.17; **HRMS (m/z): $[M]^+$** calcd. for $C_{14}H_{22}O_2$, 222.1620; found, 222.1627.

Supplementary Note 22: Stereogenic center determination (4d)

The 1-4 addition of 4-methoxyphenylboronic acid to 5-methylhex-3-en-2-one, catalyzed by (*R*-BINAP)Rh(nbd)BF_4 in presence of TEA led to **(*R*)**-4-(4-methoxyphenyl)-5-methylhexan-2-one. The configuration of the stereogenic center has been determined by comparing the specific rotation of the compound with literature ($a_D^{20.0}$= +29.0 (*c*1.200 in $CHCl_3$)).[10] The reduction of the resulting ketone was performed in the same pot with ADH110, (*R*)-selective toward acetophenone. By structural extrapolation, the second stereogenic center has been determined as (*R*).

Supplementary Note 23: (*R*)-4-(3-aminophenyl)butan-2-ol (5)

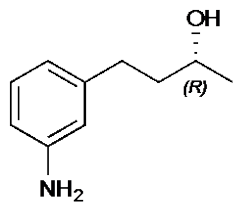

0.44 mmol scale (49.6 mg) – Yield = 75% - > 99.9% ee

yellow oil

^{1}H NMR (500 MHz, $CDCl_3$) δ 7.08 (t, J = 7.6 Hz, 1H), 6.64 – 6.60 (m, 1H), 6.58 – 6.51 (m, 2H), 3.90 – 3.79 (m, 1H), 2.73 – 2.54 (m, 2H), 1.83 – 1.69 (m, 2H), 1.27 (bs, 1H), 1.23 (d, J = 6.2 Hz, 3H).; **^{13}C NMR** (126 MHz, $CDCl_3$) δ 146.6, 143.4, 129.5, 118.9, 115.4, 112.9, 105.1, 67.7, 40.8, 32.3, 29.8, 23.8.; $\boldsymbol{a_D^{20.0}}$= -4.7 (c0.792 in $CHCl_3$); TLC (hexanes:EtOAc, 1:1 v/v): **Rf** = 0.31.

HPLC was performed on *N*-(3-(3-hydroxybutyl)phenyl)acetamide to facilitate the analysis. The acetylation was performed at 0 °C in THF (0.0038 M) in presence of acetic anhydride (1.1 equiv).

Supplementary Note 24: Benzyl-(*R*)-4-hydroxy-2-(4-(methoxycarbonyl)phenyl)piperidine-1-carboxylate (6)

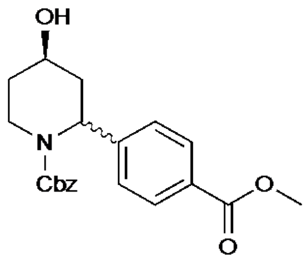

off-white solid (8.2 mol scale (3.5 kg) - purity 97% - Yield 85%

^{1}H NMR (400 MHz, $(CD_3)_2SO$) δ 7.95 (m, 2H), 7.34 (m, 7H), 5.52 (d, J = 4.6 Hz, 1H), 5.13 (d, J = 3.0 Hz, 2H), 4.83 (d, J = 4.5 Hz, 1H), 4.09 (m, 1H), 3.85 (s, 3H), 3.41 (m, 1H), 2.81 (td, J = 13.6, 2.8 Hz, 1H), 2.50 (m, 2H), 1.78 (m, 1H), 1.67 (m, 1H), 1.30 (m, 1H); **^{13}C NMR** (101 MHz, $CDCl_3$) δ 155.0, 145.7, 129.5, 128.4, 127.8, 127.4, 126.3, 66.5,

63.0, 53.6, 37.4, 34.3; **HRMS (m/z): [M]$^+$** calcd. for $C_{21}H_{23}NO_5$, 369.1576; found, 369.1601.

Supplementary references

1. Niefind, K., Müller, J., Riebel, B., Hummel, W. & Schomburg, D. The crystal structure of R-specific alcohol dehydrogenase from Lactobacillus brevis suggests the structural basis of its metal dependency. *J. Mol. Biol.* **327**, 317–328 (2003).
2. Schlieben, N. H. *et al.* Atomic resolution structures of R-specific alcohol dehydrogenase from Lactobacillus brevis provide the structural bases of its substrate and cosubstrate specificity. *J. Mol. Biol.* **349**, 801–813 (2005).
3. Leuchs, S. & Greiner, L. Alcohol Dehydrogenase from Lactobacillus brevis: A Versatile Robust Catalyst for Enantioselective Transformation. *Chem. Biochem. Eng. Q.* **25**, 267–281 (2011).
4. Abokitse, K. & Hummel, W. Cloning, sequence analysis, and heterologous expression of the gene encoding a (S)-specific alcohol dehydrogenase from Rhodococcus erythropolis DSM 43297. *Appl. Microbiol. Biotechnol.* **62**, 380–386 (2003).
5. Weckbecker, A. & Hummel, W. Cloning, expression, and characterization of an (R)-specific alcohol dehydrogenase from Lactobacillus kefir. *Biocatal. Transform.* **24**, 380–389 (2006).
6. Karabec, M. *et al.* Structural insights into substrate specificity and solvent tolerance in alcohol dehydrogenase ADH-‘A’ from Rhodococcus ruber DSM 44541. *Chem. Commun.* **46**, 6312–6316 (2010).
7. Kosjek, B. *et al.* Purification and characterization of a chemotolerant alcohol dehydrogenase applicable to coupled redox reactions. *Biotechnol. Bioeng.* **86**, 55–62 (2004).
8. Piotto, M., Saudek, V. & Sklenar, V. Gradient-tailored excitation for single-quantum NMR spectroscopy of aqueous solutions. *J. Biomol. NMR* **2**, 661–665 (1992).

9. Klumphu, P. *et al.* Micellar catalysis-enabled sustainable ppm Au-catalyzed reactions in water at room temperature. *Chem. Sci.* **8**, 6354–6358 (2017).

10. Liu, C.-C., Janmanchi, D., Chen, C.-C. & Wu, H.-L. Expanding the C1-Symmetric Bicyclo[2.2.1]heptadiene Ligand Family: Highly Enantioselective Synthesis of Cyclic β-Aryl-Substituted Carbonyl Compounds. *Eur. J. Org. Chem.* **2012**, 2503–2507 (2012).
